# Discovery of a triple-site inhibitor targeting bacterial methionyl-tRNA synthetase through combined drug repurposing screening and generative AI-assisted optimization

**DOI:** 10.1093/nar/gkag488

**Published:** 2026-05-19

**Authors:** Jingtian Su, Anjie Qiao, Weifeng Huang, Jingyi Xu, Feihu Lu, Hao Zhang, Qirui Deng, Jialin Zou, Zhen Wang, Jinping Lei, Huihao Zhou

**Affiliations:** State Key Laboratory of Anti-Infective Drug Discovery and Development, School of Pharmaceutical Sciences, Sun Yat-sen University, Guangzhou 510006, China; Guangdong Provincial Key Laboratory of Chiral Molecule and Drug Discovery, School of Pharmaceutical Sciences, Sun Yat-sen University, Guangzhou 510006, China; School of Computer Science and Engineering, Sun Yat-sen University, Guangzhou 510006, China; State Key Laboratory of Anti-Infective Drug Discovery and Development, School of Pharmaceutical Sciences, Sun Yat-sen University, Guangzhou 510006, China; Guangdong Provincial Key Laboratory of Chiral Molecule and Drug Discovery, School of Pharmaceutical Sciences, Sun Yat-sen University, Guangzhou 510006, China; State Key Laboratory of Anti-Infective Drug Discovery and Development, School of Pharmaceutical Sciences, Sun Yat-sen University, Guangzhou 510006, China; Guangdong Provincial Key Laboratory of Chiral Molecule and Drug Discovery, School of Pharmaceutical Sciences, Sun Yat-sen University, Guangzhou 510006, China; State Key Laboratory of Anti-Infective Drug Discovery and Development, School of Pharmaceutical Sciences, Sun Yat-sen University, Guangzhou 510006, China; Guangdong Provincial Key Laboratory of Chiral Molecule and Drug Discovery, School of Pharmaceutical Sciences, Sun Yat-sen University, Guangzhou 510006, China; State Key Laboratory of Anti-Infective Drug Discovery and Development, School of Pharmaceutical Sciences, Sun Yat-sen University, Guangzhou 510006, China; Guangdong Provincial Key Laboratory of Chiral Molecule and Drug Discovery, School of Pharmaceutical Sciences, Sun Yat-sen University, Guangzhou 510006, China; State Key Laboratory of Anti-Infective Drug Discovery and Development, School of Pharmaceutical Sciences, Sun Yat-sen University, Guangzhou 510006, China; Guangdong Provincial Key Laboratory of Chiral Molecule and Drug Discovery, School of Pharmaceutical Sciences, Sun Yat-sen University, Guangzhou 510006, China; School of Computer Science and Engineering, Sun Yat-sen University, Guangzhou 510006, China; School of Computer Science and Engineering, Sun Yat-sen University, Guangzhou 510006, China; State Key Laboratory of Anti-Infective Drug Discovery and Development, School of Pharmaceutical Sciences, Sun Yat-sen University, Guangzhou 510006, China; Guangdong Provincial Key Laboratory of Chiral Molecule and Drug Discovery, School of Pharmaceutical Sciences, Sun Yat-sen University, Guangzhou 510006, China; State Key Laboratory of Anti-Infective Drug Discovery and Development, School of Pharmaceutical Sciences, Sun Yat-sen University, Guangzhou 510006, China; Guangdong Provincial Key Laboratory of Chiral Molecule and Drug Discovery, School of Pharmaceutical Sciences, Sun Yat-sen University, Guangzhou 510006, China

## Abstract

Methionyl-tRNA synthetase (MetRS) plays an critical role in protein translation by catalyzing the attachment of l-methionine (l-Met) to its cognate tRNA^Met^ and has long been recognized as a valuable target for antimicrobial drug development. In this study, a drug repurposing screen of a kinase inhibitor library identified AZD8186, a clinically investigated PI3Kβ modulator, as a promising inhibitor of *Staphylococcus aureus* MetRS (*Sa*MetRS). The binding mode of AZD8186 to *Sa*MetRS was elucidated through co-crystallography, and subsequent knowledge-directed ligand optimization resulted in enhanced inhibitory activity and improved synthetic accessibility. Furthermore, we developed a novel conservation-aware and interaction-guided 3D generative AI model, designated DiffDeCIG, to facilitate structure-based drug design. DiffDeCIG modified inhibitors to establish additional interactions preferentially with conserved residues within the active pocket of *Sa*MetRS. The optimal compound, **MRS-9**, potentially competed with all three substrates of MetRS (ATP, l-Met and tRNA^Met^), and demonstrated over a 300-fold increase in inhibitory activity relative to AZD8186. Importantly, **MRS-9** selectively inhibited type 1 MetRS enzymes, while minimally affecting the tested type 2 MetRSs, including the human MetRS, thereby reducing potential adverse effects. This study reveals a novel triple-site inhibitory mechanism targeting MetRS and highlights an integrated strategy that combines knowledge-directed and AI-guided approaches in drug design.

## Graphical Abstract

**Graphical Abstract:**
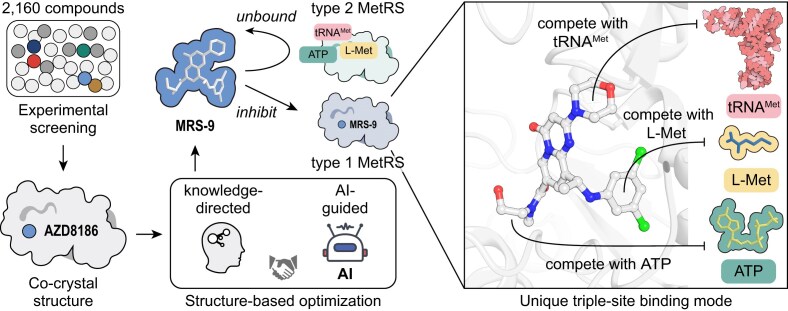

## Introduction

The rapidly increasing antimicrobial resistance (AMR) has become a critical global health challenge [1], and the development of novel antibiotics is urgently needed [2]. Aminoacyl-tRNA synthetases (aaRSs) employ conserved mechanisms to ligate specific amino acids to their cognate tRNAs, thereby generating aminoacyl-tRNAs as indispensable substrates for ribosome-mediated protein translation [3]. Due to their essential functions in protein biosynthesis, members of the aaRS family have long been considered ideal antimicrobial drug targets [4]. Methionyl-tRNA synthetase (MetRS) is the aaRS member responsible for specifically ligating l-methionine (l-Met) to both initiator and elongator tRNA^Met^ to decode AUG codons during translation [5]. Numerous MetRS inhibitors have been developed to combat Gram-positive bacteria [6, 7] and protozoan parasites [8–10], including two diaryldiamine compounds, REP8839 [11] and CRS3123 [12], which entered clinical trials. These diaryldiamine-based inhibitors simultaneously target both the l-Met binding site and a type 1 MetRS-specific auxiliary site, resulting in significant greater inhibitory potency against type 1 MetRS from bacteria and parasites than against type 2 MetRS in host human cells. However, these inhibitors, such as REP8839, often face pharmacokinetic challenges, including high plasma protein binding and low oral bioavailability. In addition, some intermediate product-mimicking inhibitors, such as 5′-*O*-[*N*-(l-methionyl)-sulfamoyl] adenosine (MetSA), bind MetRS with high affinity by simultaneously targeting the ATP and l-Met binding sites, but their application as potential antimicrobials is limited by a lack of species selectivity [13]. To date, there is an urgent need to develop new MetRS inhibitors with novel scaffolds and binding modes.

Drug repurposing screening has emerged as an attractive strategy, as it leverages de-risked compounds and offers the potential for reduced development costs and accelerated timelines [14]. Successful efforts have already identified promising antibiotics against multidrug-resistant *Acinetobacter baumannii* and *Borrelia burgdorferi* [15, 16]. Increasing attention has also been given to kinase inhibitors acting on non-kinase targets [17], both as candidate therapeutics against antibiotic-resistant pathogens [18] and, in more specific cases, as inhibitors of *Plasmodium falciparum* lysyl-tRNA synthetase [19]. Notably, both kinases and MetRS utilize ATP as a substrate, and type I/II kinase inhibitors typically bind to the ATP pocket. Therefore, screening a kinase-focused library may represent an efficient strategy to rapidly identify novel scaffolds of MetRS inhibitors, which can subsequently serve as leads for further optimization.

Recently, artificial intelligence (AI)-based generative models have been widely applied to drug design and optimization, as they can facilitate to explore vast chemical spaces and generate compounds beyond the medicinal chemists’ experience or knowledge [20–22]. Notably, 3D target-aware diffusion models have emerged as powerful tools for structure-based lead optimization [23–26], and some models with explicit binding interaction guidance are capable of generating molecules that form specific interactions with amino acid residues inside protein pocket [27, 28]. Given that the conserved residues are often functionally and/or structurally critical, targeting these residues is likely more effective in disrupting the activity of the protein target and minimizing the possibility of mutation-induced drug resistance. We expect that, by integrating evolution conservation information of protein sequences into interaction-guided 3D generative diffusion models, the new model will improve the efficiency of generating more active and less resistance-prone compounds toward their targets. This advancement is critically important for the design of anti-tumor and antimicrobial drugs.

**Figure 1.**
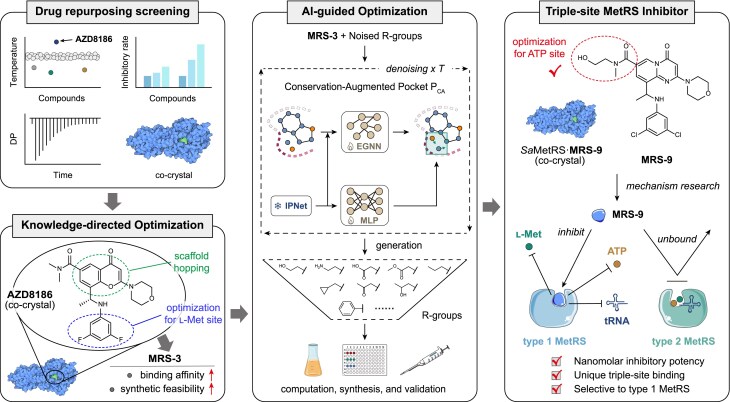
Overview of this work. Drug repurposing screening of a kinase inhibitor library, followed by biochemical and co-crystal structural analyses, identified a promising hit compound, AZD8186, against bacterial MetRS. Structure-based lead optimization directed by medicinal chemistry knowledge enhanced compounds’ inhibitory activity and synthetic accessibility. Subsequently, a novel AI model was developed to facilitate structure-based drug design. The resulting lead compound, **MRS-9**, competitively inhibited all three MetRS substrates with high selectivity for type 1 MetRSs from Gram-positive bacteria.

In this study, we initially identified AZD8186, a clinically investigated PI3K inhibitor, as a bacterial MetRS inhibitor through thermal shift assay (TSA)-based screening. Its co-crystal structure with MetRS from *S. aureus* (*Sa*MetRS) was determined, through which compound **MRS-3** with improved activity was successfully developed via knowledge-directed optimization. Furthermore, a novel conservation-aware diffusion model was developed to facilitate the growth of **MRS-3** to form interactions with the evolutionarily conserved residues in the active site cavity of *Sa*MetRS, resulting in identification of compound **MRS-9**, a nanomolar-potency inhibitor against *Sa*MetRS. Structural and biochemical analyses indicated that **MRS-9** is a novel triple-site inhibitor of MetRS with high selectivity toward type 1 MetRS in Gram-positive bacteria (Fig. 1).

## Materials and methods

### Protein and tRNA preparation

The full-length and C-terminal truncated (res. 1–520) *Sa*MetRS (UniprotKB ID: A0A0D6FXK4) and full-length MetRS from *Escherichia coli* (*Ec*MetRS; UniProtKB ID: P00959) were prepared as described [29]. Briefly, the *E. coli* BL21(DE3) cells transformed with the pET15b plasmid carrying the target gene were grown at 37°C in Luria-Bertani (LB) broth containing 100 μg/ml ampicillin, and protein expression was induced by adding 0.15 mM isopropyl-β-d-thiogalactoside (IPTG) at 20°C for 20 h. Harvested cells were lysed by sonication, and the target protein was initially purified from the supernatant using Ni-NTA column (Qiagen), followed by further purification using gel-filtration chromatography. The purified protein was assessed by SDS–PAGE, concentrated to 30 mg/ml, and stored at –80°C.

The coding DNA sequences of MetRS from *Salmonella enterica* (*Se*MetRS; UniprotKB ID: V7IMS7), MetRS from *Enterococcus faecalis* (*Ef*MetRS; UniprotKB ID: Q837B3), and N-terminal truncated human cytoplasmic MetRS (*Hc*MetRS; res. 221–900, UniProtKB ID: P56192) were cloned into pET28a(+), pET15b, and pET20b(+) plasmids, respectively. Proteins were expressed in BL21(DE3) and purified by Ni-NTA column (Qiagen) followed by gel-filtration chromatography (HiLoad 16/600 Superdex 200 pg or Superdex 200 Increase 10/300 GL, GE Healthcare). The detailed methods were described previously [30].

Total tRNA was extracted from *E. coli* cells overexpressing tRNA^Met^ to assess the tRNA aminoacylation acitity of MetRS. Specifically, the gene encoding *E. coli* tRNA^Met^ (5′-GGCTACGTAGCTCAGTTGGTTAGAGCACATCACTCATAATGATGGGGTCACAGGTTCGAATCCCGTCGTAGCCACCA-3′) was inserted into the pET20b(+) vector between the T7 promoter and terminator using In-Fusion cloning. The plasmid was then transformed into *E. coli* BL21(DE3) cells for overexpression. Transformed BL21(DE3) cells were cultured in LB medium containing 100 μg/ml ampicillin at 37°C until OD_600_ reached 0.6∼0.8. Then, tRNA^Met^ overexpression was induced by adding 1 mM IPTG, followed by cultured at 30°C for 16 h. Total tRNA in *E. coli* cells was extracted from the cell pellets using RNAiso Plus (TaKaRa) and chloroform, then precipitated from the aqueous phase with isopropanol. The resulting tRNA pellet was collected by centrifugation, washed with 75% ethanol, and dissolved in a buffer containing 20 mM Tris pH 8.0. The sample was incubated at 37°C for 2 h to facilitate deacylation. For purification, the tRNA solution was adjusted to a buffer containing 20 mM Tris pH 8.0, 10 mM MgCl_2_, and 100 mM NaCl, and subsequently loaded onto a HiTrap Q XL column (GE Healthcare). The column was eluted with a NaCl gradient, and the peak fractions eluting between 0.54 and 0.73 M NaCl were collected. Finally, the purified tRNA was concentrated to approximately 50 mg/ml and stored in a buffer containing 20 mM Tris pH 8.0, 100 mM KCl, and 10 mM MgCl_2_.

### Fluorescence-based thermal shift assay (TSA)

The stability of a protein during thermal denaturation could be assessed by its melting temperature (*T*_m_). Ligand binding often affects the thermal denaturation process of a protein, which is reflected by the shift the *T*_m_ (Δ*T*_m_), and larger Δ*T*_m_ values usually indicate higher binding affinity [31]. Reaction mixtures (20 μl final volume) containing 100 mM Tris–HCl pH 8.0, 150 mM NaCl, 2 μg of full-length *Sa*MetRS, 4 × SYPRO orange fluorescence dye (Sigma–Aldrich), and different compounds were prepared in 96-well PCR plates. After incubation at 25°C for 10 min, thermal denaturation was performed using a StepOnePlus Real-Time PCR system (Life Technologies) with 1°C/min ramping from 25°C to 95°C. Fluorescence intensity was recorded every 0.33 s, and the *T*_m_ values were calculated by fitting the melting curves using StepOne^™^ software v2.3. Δ*T*_m_ was defined as: Δ*T*_m_ = *T*_m_ (compound) − *T*_m_ (apo). For competition assays, 2 mM ATP was included in reactions with calculation adjusted to: Δ*T*_m_ = *T*_m_ (compound + ATP) − *T*_m_ (ATP). The *T*_m_ values were calculated from three independent measurements, and the average curves were used.

### Pre-transfer editing assay

A tRNA-independent pre-transfer editing assay was employed to evaluate the inhibitory activity of compounds against MetRS proteins. As an analog of l-Met, l-norleucine (l-Norleu) can be misactivated by MetRS with ATP, forming a noncognate intermediate, norleucyl adenylate, which is rapidly hydrolyzed prior to the transfer of the norleucyl moiety to tRNA^Met^. This process, known as pre-transfer editing, results in the release of l-Norleu and pyrophosphate (PPi) [29, 30]. Pyrophosphatase (PPiase) subsequently converts PPi to phosphate, which can be quantified using a malachite green assay. The amount of PPi produced in this assay reflects on the turnover rate of the reaction cycle involving l-Norleu misactivation and norleucyl adenylate hydrolysis, both occuring within the active site cavity of MetRS [32, 33]. Therefore, the inhibitory effects of compounds observed in this assay partially indicate their capacity to inhibit amino acid activation, the first step of the aminoacylation reaction catalyzed by MetRS. The 80 μl reaction was prepared by mixing 160 nM of MetRS from different species, 50 μg/ml PPiase, 1 mM DTT, and 20 mM l-Norleu in the reaction buffer (30 mM HEPES pH 7.5, 150 mM NaCl, 30 mM KCl, 40 mM MgCl_2_, and 1 mM DTT) were incubated at room temperature. Reactions were initiated by adding ATP (50 μM final) and stopped at specific time points by adding 20 μl of malachite green reagent (2.45 M sulfuric acid, 0.1% w/v malachite green, 1.5% w/v ammonium molybdate tetrahydrate, and 0.2% v/v Tween 20). Absorbance measurements at 620 nm were recorded using a Synergy H1 microplate reader (BioTek).

The inhibitory rate of a compound was calculated as (*A*_1_ – *A*_C_)/(*A*_1_ – *A*_0_) × 100%, wherein the absorbance *A*_1_ represents the positive control (no compound), *A*_0_ is negative control (no MetRS), and *A*_C_ indicates the tested compound. To determine the IC_50_ values of compounds against *Sa*MetRS, *Ef*MetRS, *Ec*MetRS, *Hc*MetRS, and *Se*MetRS, the similar pre-transfer editing assays used 2 mM l-Norleu and 30 nM MetRS, stopping reactions after 2.5 h. Dose–response relationships were established using GraphPad Prism 9 software with the log(inhibitor)-response model. Each reaction was performed in triplicate, and the results are presented as mean ± standard deviation (SD) (*n* = 3).

### ATP consumption assay

ATP consumption assay has been developed as a radioisotope-free method to evaluate the tRNA aminoacylation activity of aaRSs, including MetRS [7, 34–38]. The assay was conducted following previously reported protocols with certain modifications [7, 34, 35]. Specifically, the assay was performed in 384-well plates at room temperature. Compounds were pre-incubated for 15 min with 10 nM *Sa*MetRS in an assay buffer containing 25 mM HEPES-KOH pH 7.9, 10 mM MgCl_2_, 50 mM KCl, 0.2 mM spermine, 0.1 mg/ml bovine serum albumin, 2.5 mM dithiothreitol, 0.1 U/ml pyrophosphatase, and 2% dimethyl sulfoxide (DMSO). The reaction was initiated by adding final concentrations of 100 nM ATP, 100 μM l-Met, and 25 ng/ml total tRNA extracted from *E. coli* cells overexpressing tRNA^Met^. After 120 min, the reaction was stopped by adding an equal volume (5 μl) of Kinase-Glo reagent (Promega). The inhibitory rate of each compound was calculated using (*L*_C_ – *L*_0_)/(*L*_1_ – *L*_0_) × 100%, where *L*_1_ represents the luminescence of negative control (absence of MetRS), *L*_0_ corresponds to the positive control (absence of compound), and *L*_C_ indicates the luminescence of in the presence of the tested compound. Dose–response curves were generated using GraphPad Prism 9 software employing the log(inhibitor)-response model. Each reaction was performed in triplicate, and the results are presented as mean ± SD (*n* = 3). To evaluate species selectivity, the same assay was performed using MetRS enzymes from various species: including *Ef*MetRS, *Ec*MetRS, *Hc*MetRS, and *Se*MetRS, under identical assay conditions except for the enzyme source.

### Isothermal titration calorimetry (ITC) assay

The binding capability of AZD8186, EX229, and Fingolimod to *Sa*MetRS was assessed using a MicroCal PEAQ-ITC microcalorimeter (Malvern Panalytical). In brief, the purified *Sa*MetRS protein was dialyzed against the ITC buffer (20 mM HEPES, pH 7.5, 150 mM NaCl, and 5% glycerol) overnight, and the tested compounds were dissolved using the same ITC buffer. Then, *Sa*MetRS (20 μM) was loaded into the sample cell, and each compound (200 μM) was loaded into the syringe. Titrations were carried out at 25°C, with an initial injection of 0.2 μl followed by 18 injections of 2 μl each, at 150 s intervals. The dissociation constant (*K*_d_) was determined by fitting the calorimetric data to a one-site binding model using the MicroCal PEAQ-ITC analysis software. All titrations were repeated at least twice.

### Surface plasmon resonance (SPR)

Small molecule binding kinetics were quantified using a Biacore 8K SPR system (Cytiva) with CM5 sensor chips. The full-length *Sa*MetRS was immobilized on flow cell 2 via amine coupling (EDC/NHS activation), achieving ∼15 000 response units (RU), while flow cell 1 served as a reference. All experiments were performed at 25°C in PBS running buffer (pH 7.4) at 30 μl/min flow rate. Compound solutions (serially diluted in PBS containing 5% DMSO) were injected across both flow cells for 100 s association and 150 s dissociation phases. Sensorgrams were processed with double-referencing solvent correction (buffer and reference channel subtraction). Equilibrium dissociation constants (*K*_d_) were calculated through steady-state dose–response analysis using Biacore Insight Evaluation Software. The binding measurement for each compound was repeated at least twice.

### Crystallography

The sitting-drop vapor-diffusion method was employed to crystallize the C-terminal truncated *Sa*MetRS with compounds. *Sa*MetRS (20 mg/ml) was incubated with 0.3 mM of AZD8186, **MRS-3**, or **MRS-9** on ice for 30 min. Crystallization drops were prepared by mixing 1 μl of protein and 1 μl of reservoir solution, and then equilibrated against 80 μl of reservoir solution at 16°C for 2–5 days. The reservoir solution for crystallizing the *Sa*MetRS·AZD8186 complex contains 0.1 M sodium cacodylate pH 6.5, and 22% w/v PEG 3350, and the reservoir solution for crystallizing the *Sa*MetRS·**MRS-3** and *Sa*MetRS·**MRS-9** complexes is consisting of 0.05 M lithium acetate, 0.1 M HEPES pH 7.5, and 25% w/v PEG 3350. The crystals were cryoprotected in reservoir solution supplemented with 20% (v/v) ethylene glycol and then flash frozen in liquid nitrogen.

The crystals of *Sa*MetRS·AZD8186 and *Sa*MetRS·**MRS-3** complexes were diffracted on a Rigaku Oxford Diffraction Xcalibur Nova single crystal diffractometer with a wavelength of 1.5418 Å at 100 K, and the diffraction data were integrated and scaled using CrysAlisPro software (Agilent Technologies UK Ltd.). The X-ray diffraction data of the *Sa*MetRS·**MRS-9** complex were collected at the BL19U1 beamline of the Shanghai Synchrotron Radiation Facility (SSRF), the National Facility for Protein Science in Shanghai (NFPS), and processed with XDS [39] and Aimless [40]. Molecular replacement was performed using MOLREP [41] with the structure of *Sa*MetRS (PDB code: 7WPJ) as the search model. Iterative refinements were conducted using WinCoot [42] and Refmac5 [43]. The stereochemical quality of the final models were validated using MolProbity [44]. The statistics of data collection and structure refinements are provided in Supplementary Table S1. The atomic coordinates and structure factors have been deposited in the Protein Data Bank (PDB) under the accession codes of 9V9D, 9V9F, and 9V9M.

### Development of the conservation-aware model

The development of the DiffDeCIG model, including dataset construction, preliminary, conservation-aware condition, interaction-prior guidance, forward diffusion process, and reverse denoising process is described in detail in the Supplementary Information.

### Molecular docking

The co-crystal structure of the *Sa*MetRS·AZD8186 complex was used as the template for molecular docking. The protein structure was processed using the Protein Preparation Workflow in Schrödinger Suite 13.1, which involved removal water molecules, completion missing residues, assignment of protonation and charge states, and energy minimization. A docking grid was then generated, and ligands were prepared using the LigPrep and Glide modules. Molecular docking was carried out using Glide XP mode. Specifically, for the DiffDeCIG generated compounds, the Refine module was employed to obtain more compatible binding conformations and calculate docking scores.

### Chemical synthesis

Detailed synthetic procedures for all compounds are provided in the Supplementary Information.

### Antibacterial susceptibility assays

The minimum inhibitory concentrations (MICs) of the tested compounds were determined against *S. aureus* (ATCC25923) and *E. coli* (ATCC25922) using the broth microdilution method in Mueller–Hinton broth (MHB), according to CLSI (Clinical and Laboratory Standards Institute) guidelines. Briefly, bacterial suspensions were adjusted to approximately 5 × 10^5^ CFU/ml in MHB and inoculated into 96-well plates containing 2-fold serial dilutions of compounds. The 96-well plates were incubated at 37°C for 16–20 h. MIC values were defined as the lowest compound concentration at which no visible bacterial growth was observed by visual inspection, and further confirmed by measuring optical density at 600 nm (OD_600_). The MIC values of all tested compounds were determined in triplicate.

For time–growth assays, bacteria were cultured in microplates under static conditions similar to those used for MIC determination, with compound concentrations fixed at 64 µg/ml. *S. aureus* cultures were inoculated into MHB supplemented with test compounds and incubated at 37°C in a static incubator. The OD_600_ values was recorded every 2 h from 0 to 24 h using a microplate reader to monitor bacterial growth. The time–growth assays were performed in triplicate, and the results are presented as mean ± SD (*n* = 3).

### Data analysis and figure preparation

All data were analyzed using GraphPad Prism 9.0 software and are expressed as the mean ± SD (*n* = 3), unless otherwise specified. All protein structure figures were prepared using PyMOL (https://www.pymol.org/).

## Results

### Experimental screening repurposes a kinase inhibitor to bacterial MetRS

**Figure 2.**
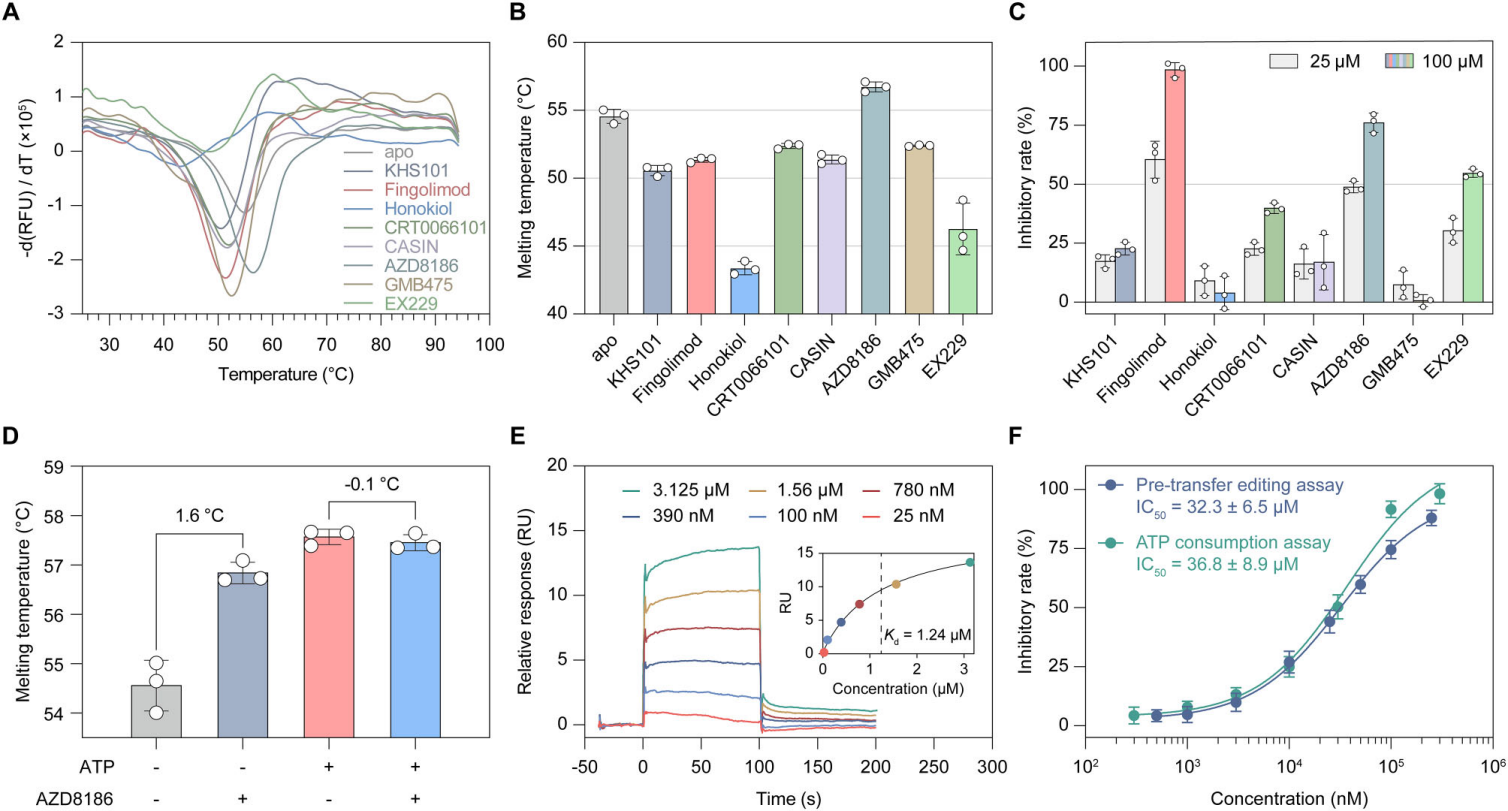
Activity characterization of hit compounds identified by TSA-based screening. (**A**) Representative melting curves of *Sa*MetRS with or without different hit compounds. (**B**) *T*_m_ values of *Sa*MetRS with or without hit compounds derived from the melting curves. Results are presented as mean ± SD (*n* = 3). (**C**) Inhibitory rates of hit compounds against *Sa*MetRS were assessed at two concentrations using pre-transfer editing assay. For GMB475, due to limited solubility, the inhibitory rate at the high concerntration was determined using 50 μM compound dissolved in 5% DMSO. Each concentration was tested in triplicate, and the data are presented as mean ± SD (*n* = 3). (**D**) Thermal stabilization of *Sa*MetRS by AZD8186 with or without 2 mM ATP, indicating competition between AZD8186 and ATP for binding to *Sa*MetRS. Each reaction was conducted in triplicate, and the results are expressed as mean ± SD (*n* = 3). (**E**) SPR relative response (RU) curves of *Sa*MetRS in the presence of various concentrations of AZD8186 and the corresponding fitting curve (inset). (**F**) Inhibition curves of *Sa*MetRS activity by AZD8186, as determined by the pre-transfer editing assay and ATP consumption assay. Each concentration was tested in triplicate, and the data are presented as mean ± SD (*n* = 3).

A library of 2160 kinase inhibitors (TargetMol, Cat No. L1600) was utilized as a focused set for identifying new scaffold inhibitors against bacterial type 1 MetRS, since both kinases and MetRS use ATP as a substrate. Compounds that altered the melting temperature (*T*_m_) of apo *Sa*MetRS by >1°C were considered positive hits, resulting in the identification of eight hit compounds (Fig. 2A and B, and Supplementary Table S2). While seven compounds decreased the *T*_m_ value by >1°C, AZD8186 was the only compound that induced a significant positive *T*_m_ shift of *Sa*MetRS (Δ*T*_m_ = 2.2°C). These eight hit compounds were tested enzyme inhibition activity using a pre-transfer editing assay, and three of them, including AZD8186, demonstrated dose-dependent inhibition against *Sa*MetRS and reached >50% inhibition at 100 μM (Fig. 2C). Isothermal titration calorimetry (ITC) confirmed that, of the three compounds exhibiting considerable inhibitory activity, only AZD8186 exhibited measurable binding enthalpy changes, with a *K*_d_ value of 6.2 ± 1.5 μM for *Sa*MetRS (Supplementary Fig. S1A–C). Notably, our previous studies, along with that of others, have revealed that the substrate ATP often plays a positive role in facilitating the binding of ligands to type 1 MetRS, which could increase the hit rates in compound screening and enhance the binding affinity of various diaryldiamine inhibitors [29, 30, 45]. Consequently, the ITC experiments for these three compounds were then conducted in the presence of 5 mM ATP. However, ATP-facilitated binding was not observed for any of the three compounds (Supplementary Fig. S1D–F). In contrast, the binding of AZD8186 to *Sa*MetRS was impaired by the high concentration of ATP (Supplementary Fig. S1D), which is different from the ATP-assisted binding of the diaryldiamine inhibitors. A TSA assay was conducted using the *Sa*MetRS saturated with 2 mM ATP. While 2 mM ATP alone increased the *T*_m_ of *Sa*MetRS by over 3.0°C, the addition of 100 μM of AZD8186 did not further enhance the *T*_m_ of ATP-saturated *Sa*MetRS (Fig. 2D), indicating that AZD8186 and ATP cannot co-bind to *Sa*MetRS. AZD8186 is a selective inhibitor of PI3Kβ that has entered a Phase II clinical trial for the treatment of advanced solid tumors [46, 47]. A close analogue of AZD8186 has been shown to bind to the ATP pocket of PI3Kβ (PDB code: 4URK). These findings suggested that AZD8186 may utilize a novel inhibitory mechanism, likely involving the ATP binding site, to inhibit *Sa*MetRS.

Consequently, AZD8186 was selected for further evaluation and optimization. The binding affinity of AZD8186 to *Sa*MetRS was further confirmed using surface plasmon resonance (SPR), giving a *K*_d_ value of 1.24 μM (Fig. 2E). The IC_50_ value of AZD8186 against *Sa*MetRS was determined to be 32.3 ± 6.5 μM and 36.8 ± 8.9 μM by the pre-transfer editing assay and ATP consumption assay, respectively (Fig. 2F). These IC_50_ values obtained from these two assays are well consistent to each other. The observed discrepancy between the IC_50_ and *K*_d_ values may be attributed to competition between AZD8186 and the high concentrations of substrate l-Met (100 μM) and the non-cognate l-Norleu (2 mM) present in the enzymatic reaction mixtures.

### The inhibitory mechanism of AZD8186 against MetRS

**Figure 3.**
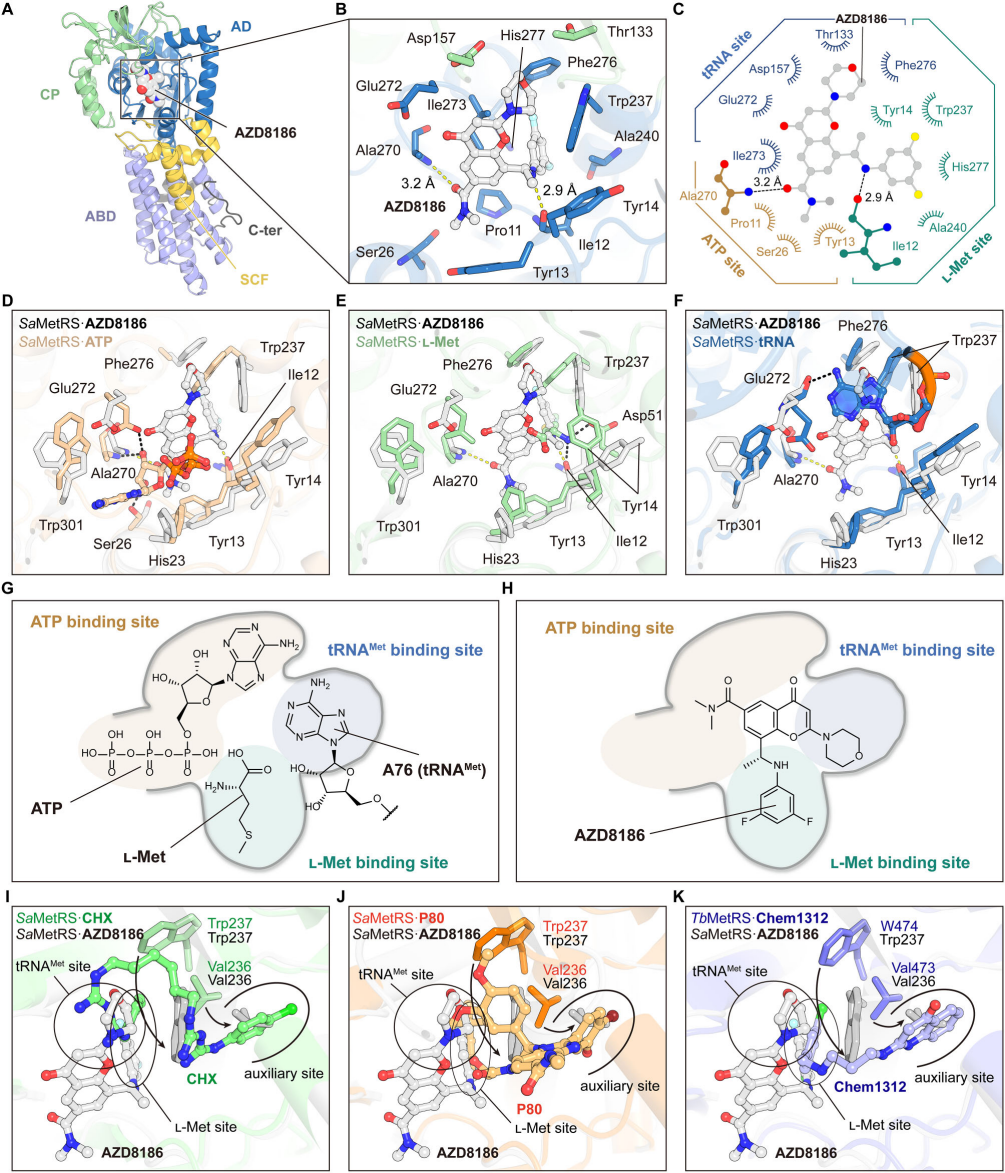
The binding mode of AZD8186 within the active site cavity of *Sa*MetRS. (**A**) The overall structure of *Sa*MetRS in complex with AZD8186. (**B**) Detailed interactions between AZD8186 and *Sa*MetRS. (**C**) A 2D schematic depicting the interactions of AZD8186 with *Sa*MetRS. (**D**) Structural comparison of the AZD8186-bound and the ATP-bound (PDB code: 7WPL) *Sa*MetRS structures within the active site cavity. (**E**) Structural comparison of the AZD8186-bound and the l-Met-bound (PDB code: 7WPK) *Sa*MetRS structures. (**F**) Structural comparison of the AZD8186-bound and the tRNA^Met^-bound (modeled by Alphafold 3)* Sa*MetRS structures. (**G**) A schematic representation of the MetRS active pocket for the binding of three substrates. (**H**) A schematic representation showing that AZD8186 binds to all three substrate binding sites. (**I–K**) Distinct to MetRS bound with auxiliary site inhibitors chlorhexidine (CHX, PDB code: 8XM4) (**I**), P80 (PDB code: 7WPI) (**J**), and Chem1312 (PDB code: 4EG5) (**K**), *Sa*MetRS bound with AZD8186 displays a closed auxiliary site. This closure results from a unique conformational rearrangement of Val236 and Trp237 and may be specific to the triple-site inhibitory mechanism of AZD8186.

The binding mode of AZD8186 was elucidated through a co-crystal structure of *Sa*MetRS in complex with AZD8186 at 2.5 Å resolution (Supplementary Fig. S2A and Supplementary Table S1). AZD8186 binds within the active site cavity of *Sa*MetRS (Fig. 3A), wherein its carbonyl group and secondary amine form hydrogen bonds (H-bonds) with Ala270 and Ile12, respectively (Fig. 3B and C). The benzopyranone core and the *N,N*-dimethyl group of AZD8186 engage in hydrophobic interactions with Ile273 and Tyr13, respectively. Additionally, the dichlorophenyl moiety of AZD8186 forms extensive hydrophobic contacts with Ile12, Ala270, Phe276, and His277 (Fig. 3B and C). Structural comparisons of *Sa*MetRS in the AZD8186-bound state with its apo form (PDB code: 7WPJ), ATP-bound form (PDB code: 7WPL), and l-Met-bound form (PDB code: 7WPK) revealed that the knuckle structure within the CP domain of *Sa*MetRS in the AZD8186-bound state closely resembles that observed in the l-Met-bound state but differs from those in the apo and ATP-bound states, resulting in a more closed conformation of the active site cavity (Supplementary Fig. S3). This conformational change of the CP domain is associated with amino acid activation and the positioning of the 3′ CCA end of tRNA^Met^ within the active site cavity, which displays significant differences between type 1 and type 2 MetRS [29, 48]. While the 3′ CCA end of tRNA^Met^ is disorder in the only available MetRS·tRNA^Met^ complex structure (PDB code: 2CT8) [49], we employed AlphaFold 3 to construct and assess a reasonable structural model of the full-length *Sa*MetRS·tRNA^Met^ complex (Supplementary Fig. S4) [50]. Comparison between the *Sa*MetRS·AZD8186 and *Sa*MetRS·tRNA^Met^ complexes indicated that the closed conformation of the knuckle structure in the *Sa*MetRS·AZD8186 complex is shifted downward by an approximately 10 Å relative to its position in the tRNA^Met^-bound *Sa*MetRS, which likely prevents the 3′ CCA end of tRNA^Met^ from accessing the active site cavity and undergoing aminoacylation (Supplementary Fig. S4).

Structural comparison between the *Sa*MetRS·AZD8186 complex and the *Sa*MetRS·ATP complex reveals that AZD8186 occupies the ATP ribose-binding site and forms an H-bond with Ala270 (Fig. 3D), thereby explaining its competition with ATP (Fig. 2F). Meanwhile, AZD8186 fully occupies the l-Met binding site (Fig. 3E). Structural alignment of the *Sa*MetRS·AZD8186 complex with the predicted *Sa*MetRS·tRNA^Met^ complex suggests that AZD8186 likely occupies the A76 adenine-binding site of *Sa*MetRS (Fig. 3F). Consequently, AZD8186 likely partially overlaps with all three substrate when binding within the active site cavity of *Sa*MetRS, potentially representing a novel triple-site inhibitory mechanism against MetRS (Fig. 3G and H). Notably, the chemical moieties through which AZD8186 occupies these three substrate-binding sites, as well as their detailed interactions with the active site residues, differ from those of the natural substrates (Fig. 3D–F). Moreover, the binding of AZD8186 induces a unique conformation of Trp276 and results in closure of the auxiliary site, distinguishing AZD8186 from the amino acid-auxiliary dual-site inhibitory mechanism of typical diaryldiamine inhibitors (Fig. 3I–K). Therefore, AZD8186 constitutes a novel MetRS inhibitor with a unique binding mode, warranting further optimization to enhance its activity.

### Structure-guided lead optimization employing medicinal chemistry knowledge

**Figure 4.**
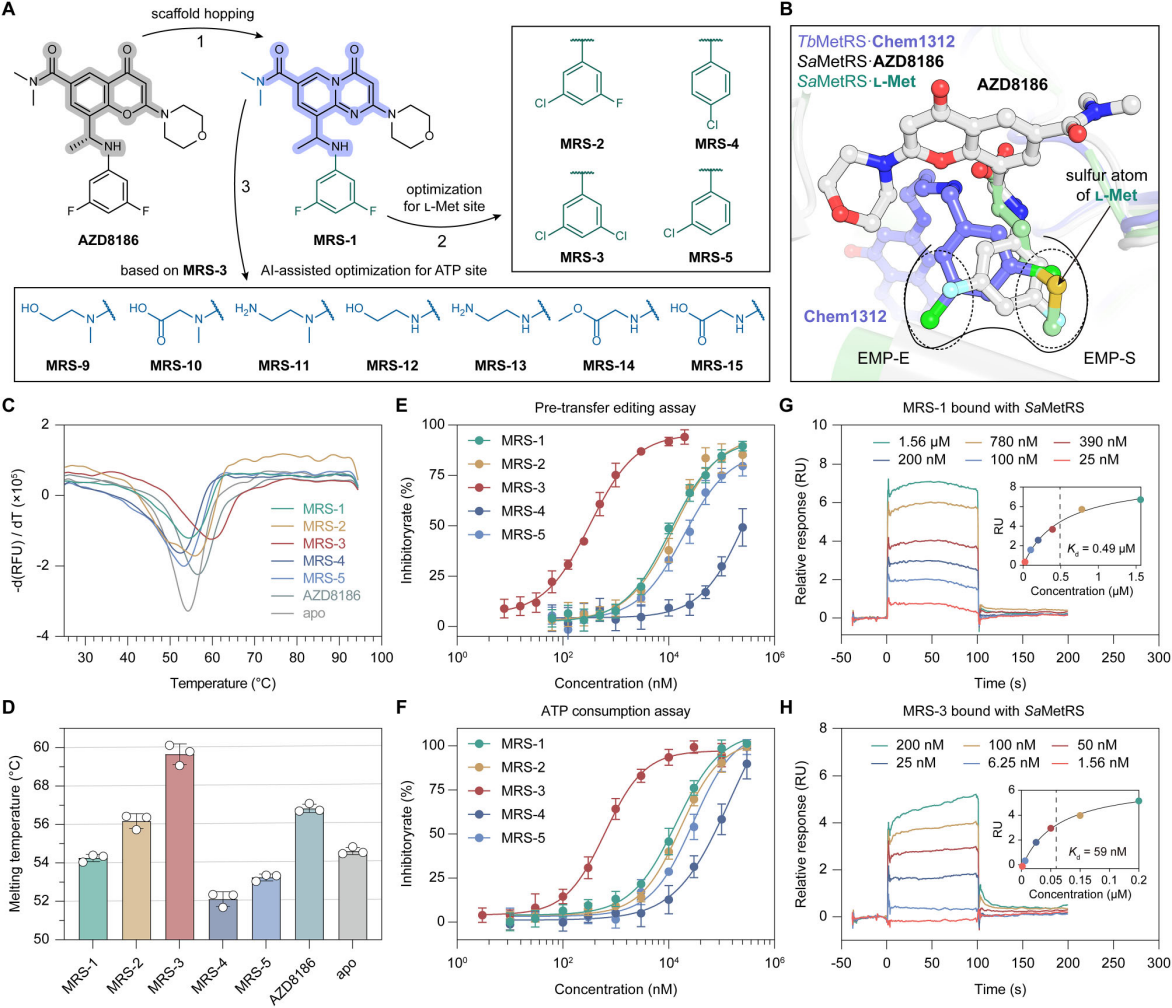
Structures and activity data of compounds derived from knowledge-based optimization. (**A**) Chemical structures of compounds designed through knowledge-directed and AI-guided optimization. (**B**) Structural comparison of AZD8186, Chem1312, and l-Met regarding their occupancy at the amino acid binding site. (**C**) Representative melting curves of *Sa*MetRS in the presence or absence of compounds **MRS-1** to **MRS-5**. (**D**) The *T*_m_ values of *Sa*MetRS in the presence or absence of compounds **MRS-1** to **MRS-5**, derived from the melting curves. The results are presented as mean ± SD (*n* = 3). (**E**) IC_50_ curves of compounds **MRS-1** to **MRS-5**, determined by pre-transfer editing assay. The results are presented as mean ± SD (*n* = 3). (**F**) IC_50_ curves of compounds **MRS-1** to **MRS-5**, as determined by the ATP consumption assay. The results are presented as mean ± SD (*n* = 3). (**G**) SPR relative response curves of *Sa*MetRS in the presence of varying concentrations of **MRS-1**, along with the corresponding fitting curve (inset). (**H**) SPR relative response curves of *Sa*MetRS in the presence of different concentrations of **MRS-3**, along with the corresponding fitting curve (inset).

Guided by the co-crystal structure with *Sa*MetRS, the optimization of AZD8186 was initially conducted based on medicinal chemistry knowledge. Structural analysis revealed that the binding of AZD8186 to *Sa*MetRS is primarily driven by H-bonding interactions with Ile12 and Ala270, as well as hydrophobic interactions between its benzopyranone core and Ile273 (Fig. 3B and C). Initially, the benzopyranone core of AZD8186 was replaced with a pyrido[1,2-a]pyrimidine scaffold, which is more synthetically accessible and amenable to modification, resulting in the compound **MRS-1** (Fig. 4A). Moreover, previous studies have shown that upon ligand binding, the l-Met binding pocket within the active site cavity of type 1 MetRS (e.g. *Trypanosoma brucei* MetRS, *Tb*MetRS) expands to form an “enlarged l-Met pocket” (EMP) [51]. This EMP comprises two sub-pockets: one accommodating the l-Met side chain, particularly the sulfur atom (EMP-S), and another forming the enlarged portion compared to the l-Met-bound structure (EMP-E) [52]. Although the conformation of the difluorobenzene group of AZD8186 differs slightly from the corresponding moieties of classic diaryldiamine inhibitors, such as the dichlorobenzene group of Chem1312 [8], the two sub-pockets (EMP-S and EMP-E) remain conserved (Fig. 4B). Extensive structural optimization of diaryldiamine inhibitors has concluded that the single or dual chlorinated benzene groups are preferable for the EMP of type 1 MetRS [9, 10, 53]. Therefore, the difluorobenzene group of **MRS-1** was modified to chlorinated benzene structures, yielding the compounds **MRS-2, MRS-3, MRS-4**, and **MRS-5** (Fig. 4A).

The Δ*T*_m_ values of *Sa*MetRS caused by **MRS-1** to **MRS-5** were determined to be −0.1, 1.3, 5.3, −2.1, and −1.4°C (Fig. 4C and D). Although **MRS-1** exhibited minimal effect on the *T*_m_ value of *Sa*MetRS, its IC_50_ value for *Sa*MetRS was determined to be 9.8 ± 1.8 μM (Fig. 4E), representing an approximately 3-fold improvement in inhibitory activity compared to AZD8186, supporting the use of the scaffold hopping strategy in the inhibitor optimization. While **MRS-3** showed the greatest stabilization effect on *Sa*MetRS among these five compounds (Fig. 4C and D), it also demonstrated most potent inhibitory activity in the pre-transfer editing assay, with an IC_50_ value of 0.33 ± 0.05 μM superior to the values of 9.8 ± 1.8 μM for the **MRS-1**, 12.6 ± 2.3 μM for **MRS-2**, >100 μM for **MRS-4**, and 19.5 ± 4.9 μM for **MRS-5** (Fig. 4E). Consistently, with the ATP consumption assay, the IC_50_ values for **MRS-1** to **MRS-5** were determined to be 13.8 ± 2.7, 16.8 ± 2.7, 0.63 ± 0.12, >100, and 28.8 ± 6.7 μM, respectively, thereby further supporting **MRS-3** as the most potent inhibitor (Fig. 4F). Moreover, the SPR analysis revealed that the *K*_d_ value of **MRS-3** binding to *Sa*MetRS was 59 nM, indicating approximately 8-fold and 21-fold greater affinity than **MRS-1** (*K*_d_ = 0.49 μM) and AZD8186 (*K*_d_ = 1.24 μM), respectively (Fig. 4G–H and Fig. 2E). Collectively, these results demonstrated that **MRS-3** consistently exhibited superior activity across all assays, identifying it as a promising lead compound for further development.

Co-crystal structural analysis revealed that the amide group of AZD8186 occupies the site corresponding to the ribose moiety of ATP, leaving the adjacent adenosine binding site of *Sa*MetRS unoccupied (Fig. 3D). Conventional intermediate analogue inhibitors exhibit strong inhibitory activity by fully occupying the amino acids and ATP-binding sites; for example, MetSA exhibits high potentcy (IC_50_ = 7 nM) against *E. coli* MetRS (*Ec*MetRS) [54]. To gain additional interactions with residues at the adenine-binding site of *Sa*MetRS, substituted aromatic groups were linked to the amide of **MRS-3**, resulting in compounds **MRS-6, MRS-7**, and **MRS-8** (Supplementary Fig. S5). Molecular docking analyses supported the proposed binding modes of these compounds within the active site cavity of *Sa*MetRS (Supplementary Fig. S5). However, all three compounds nearly completely lost their binding ability and inhibitory activity against *Sa*MetRS (Supplementary Fig. S6). We resolved the co-crystal structure of the *Sa*MetRS·**MRS-3** complex (Supplementary Fig. S2B and Supplementary Table S1), revealing that **MRS-3** adopts a binding mode similar to that of AZD8186 (Supplementary Fig. S7). This finding indicates that scaffold hopping from benzopyranone to pyrido[1,2-a]pyrimidine preserves compatibility with the conserved binding pocket. Structural alignment of the *Sa*MetRS·**MRS-3** complex with the *Sa*MetRS·ATP complex (PDB code: 7WPL) revealed notable conformational differences in the residues of the ATP binding site. The differences were particularly pronounced in the residues Tyr13, Glu272, Trp301, and the “HIGH” motif, one of the two signature motifs of class I aaRS members, in **MRS-3**-bound *Sa*MetRS, compared to the corresponding residues in ATP-bound *Sa*MetRS (Supplementary Fig. S8). These differences likely explain why structure-guided linking of adenine-mimicking fragments to **MRS-3** did not effectively target the evolutionarily conserved binding site for the ATP adenine moiety. To address this limitation, we developed a novel diffusion model that integrates evolutionary information and interaction guidance for AI-assisted molecular generation, which we subsequently employed to facilitate the further optimization of compound **MRS-3** based on the co-crystal structure of the *Sa*MetRS·**MRS-3** complex.

### Development of a conservation-aware and interaction-guided 3D generative model DiffDeCIG

Our recent study successfully developed a deep generative model, DiffDec, which integrates 3D pocket constraints for ligand scaffold decoration within the framework of a denoising diffusion probabilistic model [26]. In contrast to many reported lead optimization models that rely primarily on the chemical structures of known binding ligands [55], DiffDec directly utilizes the target structure to guide the growth of lead compound to better fit with the binding pocket, making it particularly well-suited for structure-based optimization of our newly identified triple-site MetRS inhibitors. Furthermore, it is widely recognized that the conserved residues are often functionally and/or structurally critical and exhibit lower mutation rates. Consequently, compounds interacting with such residues are more likely to reduce the mutation-induced drug resistance, a factor that is crucial for antimicrobial drug discovery. Based on DiffDec [26], we developed a novel diffusion model, named DiffDeCIG (Diffusion-based scaffold Decoration model with Conservation-aware and Interaction-prior Guidance).

**Figure 5.**
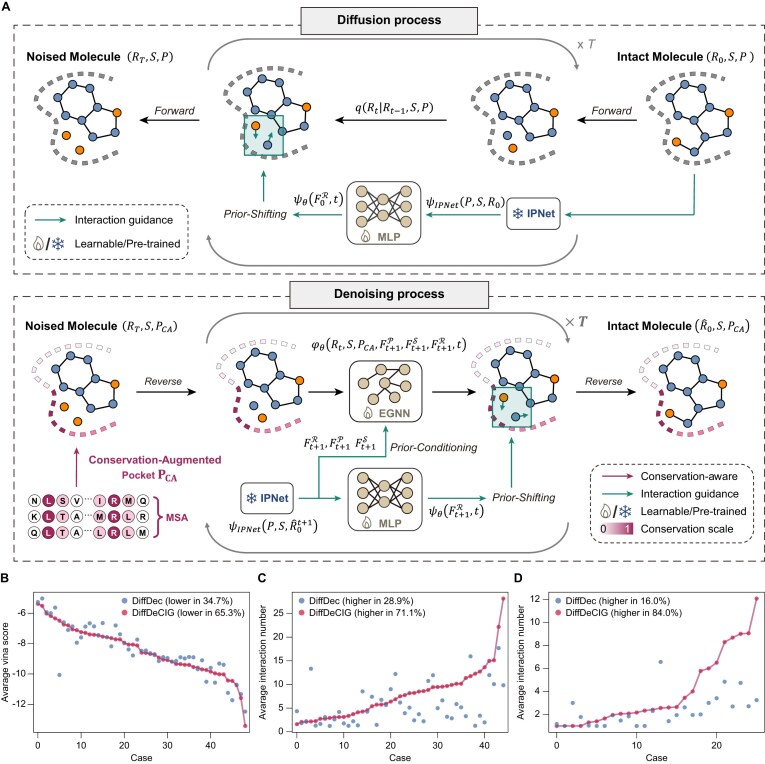
Overview and evaluation of DiffDeCIG. (**A**) Schematic illustration of the DiffDeCIG framework. (**B**) Comparison of the average Vina scores between DiffDeCIG and DiffDec across 49 proteins in the test set. (**C**) Comparison of the average interaction frequencies of ligands generated by DiffDeCIG and DiffDec within the test set. (**D**) Comparison of interaction frequencies for cases involving highly conserved pocket regions in the test set (25 cases with residues conservation score >0.4).

DiffDeCIG is built on the publicly accessible and widely adopted CrossDocked dataset [56]. In comparison with DiffDec, DiffDeCIG introduces additional protein–ligand interaction priors and amino acid conservation information within the binding pocket to direct molecular scaffold decoration, thereby enhancing binding-related properties (Fig. 5A). To ensure broad applicability, DiffDeCIG employs a well-established procedure to calculate conservation scores for each pocket. Specifically, amino acid residue conservation scores are predicted via multiple sequence alignment using HHblits [57] against the UniRef30_2023_02 database and are incorporated as additional features of protein pocket representation. Concurrently, a publicly available interaction-prior neural network (IPNet) [28] built upon SE(3)-equivariant neural networks with cross-attention layers, is pretrained to capture the protein–ligand interactions computed from pocket–ligand complexes under supervision on binding affinity signals. During the forward diffusion process, continuous Gaussian noise is added to the atomic coordinates and types of R-group via a variance-preserving cosine schedule [28], and the protein–ligand interactions pretrained by IPNet are formulated as interaction-based shifting in atomic coordinates along the diffusion trajectory. DiffDeCIG learns to predict both noise and interaction-based shifts within conservation-augmented pocket and scaffold. Once trained, the model can be applied to generate R-groups for arbitrary protein pockets and scaffolds. For example, given a pocket–scaffold complex, residue conservation scores are first computed to obtain a conservation-augmented pocket. DiffDeCIG then samples a set of candidate R-groups from a Gaussian distribution. During sampling, both the conservation-augmented pocket and protein–ligand interactions from IPNet are jointly integrated to guide the generation and position adaptation of R-groups, thereby enhancing non-covalent interactions with conserved residues (Fig. 5A). In this framework, DiffDeCIG is jointly conditioned on the pocket–scaffold complex and the residue conservation scores during both training and sampling, enabling the model to capture key binding-mode features. This design makes DiffDeCIG a generalizable method rather than a target-specific model restricted to a particular class of targets. In addition, this joint conditioning scheme may improve model robustness and help maintain chemically reasonable R-group generation even when the conservation signals are perturbed.

Notably, DiffDeCIG significantly outperforms DiffDec in generating molecules with higher binding affinity, as assessed using AutoDock Vina scores, and in establishing more binding interactions with conserved residues (Fig. 5B–D and Supplementary Table S3). Across 49 protein targets in the test set, DiffDeCIG achieves superior binding affinity in 65.3% of protein targets in the CrossDocked dataset [56], whereas DiffDec outperformed in only 34.7% of targets (Fig. 5B). This improvement is attributed to the ability of DiffDeCIG to generate molecules that form a great number of non-covalent interactions (including H-bonding, halogen bonding, π–π and hydrophobic interactions, and salt bridges) with residues in the binding pocket. Molecules generated by DiffDeCIG exhibited increased interactions with the active site residues in 71.1% of targets (Fig. 5C), and this rising to 84.0% when considering interactions exclusively with conserved residues (Fig. 5D), indicating effective integration of conservation information during molecular generation. Collectively, these results underscored the superior performance of DiffDeCIG in lead optimization via R-group decoration, primarily by enhancing interactions with conserved residues.

### DiffDeCIG-aided inhibitor optimization and evaluation

**Figure 6.**
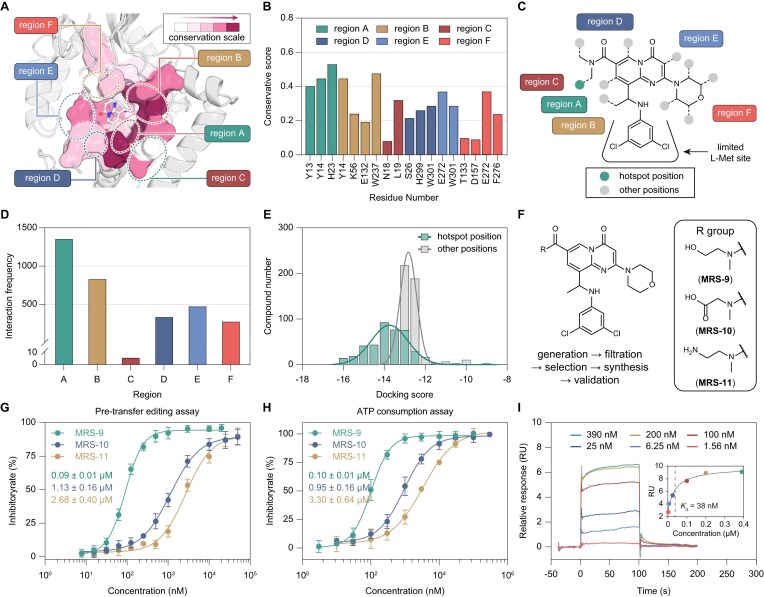
DiffDeCIG-aided optimization of **MRS-3** and its experimental validation. (**A**) The ligand binding pocket of *Sa*MetRS can be divided to six regions with different conservation. (**B**) Conservation scores of the residues in each region. (**C**) Ten R-group growth positions on **MRS-3** face to six regions of the binding pocket with different conservation. (**D**) Frequency of binding interactions between generated molecules and protein residues in each binding region. (**E**) Comparison of refined docking scores between compounds modified at the highly conserved and synthetic accessible hotspot position and those modified at other positions. (**F**) Chemical structures of three AI-generated compounds selected for synthesis and validation. (**G**) IC_50_ curves of compounds **MRS-9** to **MRS-11**, determined using a pre-transfer editing assay. The results are presented as mean ± SD (*n* = 3). (**H**) IC_50_ curves of compounds **MRS-9** to **MRS-11**, determined using an ATP consumption assay. The results are presented as mean ± SD (*n* = 3). (**I**) SPR relative response (RU) curves of *Sa*MetRS in the presence of various concentrations of **MRS-9**, accompanied by the corresponding fitting curve (inset).

We utilized the co-crystal structure of the *Sa*MetRS·**MRS-3** complex as input for the trained DiffDeCIG model. A total of 1000 compounds were generated by decorating on ten positions of **MRS-3**, which form interactions with amino acid residues across six regions exhibiting varying degrees of conservation (Fig. 6A–C). The generated molecules were grouped into six classes according to the region with which their decorated R-groups interacting. Notably, molecules gaining new interactions with the most conserved region, designed as region A, outnumbered those interacting the other five regions (Fig. 6C and D). Furthermore, 356 molecules decorated at the hotspot position of **MRS-3** adjacent to region A exhibited higher binding affinities, as assessed using AutoDock Vina scores, than other molecules (Fig. 6E). These 356 molecules were subsequently filtered based on the following criteria: (i) formation of interactions with the conserved residue His23; (ii) a predicted binding score of ≤−13.5 calculated by Schrödinger 2022 (Fig. 6E); and (iii) a synthetic accessibility (SA) score threshold of ≥0.73 (Supplementary Fig. S9). Finally, three compounds, **MRS-9, MRS-10**, and **MRS-11**, bearing the methanol, formic acid, and methylamine side-chain groups, respectively, were selected for further chemical synthesis and *in vitro* activity evaluation (Fig. 6F).

In the TSA assays, **MRS-10** and **MRS-11** increased the Δ*T*_m_ of *Sa*MetRS by 5.7°C and 2.4°C, respectively, whereas **MRS-9** induced a significantly greater Δ*T*_m_ of 11.8°C, surpassing the 8.8°C shift observed with **MRS-3** (Supplementary Fig. S10). Further validation by the pre-transfer editing assay revealed IC_50_ values of 0.09 ± 0.01 μM for **MRS-9**, 1.13 ± 0.16 μM for **MRS-10** and 2.68 ± 0.40 μM for **MRS-11**, whereas the ATP consumption assay revealed IC_50_ values of 0.10 ± 0.01 μM for **MRS-9**, 0.95 ± 0.16 μM for **MRS-10**, and 3.30 ± 0.64 μM for **MRS-11** (Fig. 6G and H). Consequently, compound **MRS-9** demonstrated a 4- to 6-fold increase in potency compared to **MRS-3** in inhibiting *Sa*MetRS. Consistently, SPR experiments determined a *K*_d_ value of 38 nM for **MRS-9** binding to *Sa*MetRS, outperforming **MRS-3** (*K*_d_ = 59 nM) (Fig. 6I and Fig. 4H). Considering synthetic accessibility, the model input was traced back to select the amide nitrogen of **MRS-3** for decoration, resulting in the synthesis and evaluation of four additional compounds (Supplementary Fig. S11A). TSA assays indicated that **MRS-12, MRS-13, MRS-14**, and **MRS-15** induced Δ*T*_m_ values of 1.6°C, −0.8°C, 1.4°C, and 1.6°C, respectively (Supplementary Fig. S11B and C). The pre-transfer editing assay showed IC_50_ values of 1.8 ± 0.3, 4.7 ± 0.5, 3.0 ± 0.3, and 1.9 ± 0.5 μM for **MRS-12** to **MRS-15**, respectively (Supplementary Fig. S11D). Using the ATP consumption assay, the IC_50_ values of **MRS-12** to **MRS-15** were determined to be 0.75 ± 0.09, 7.5 ± 2.0, 3.0 ± 0.4, and 3.3 ± 0.8 μM, respectively (Supplementary Fig. S11E). Although these compounds were less potent than **MRS-9**, they represent a clear improvement over the previously manually designed compounds **MRS-6** to **MRS-8** (Supplementary Fig. S5 and Supplementary Fig. S6).

### Triple-site inhibitors selectively target type 1 MetRS and exhibit antibacterial potential

**Figure 7.**
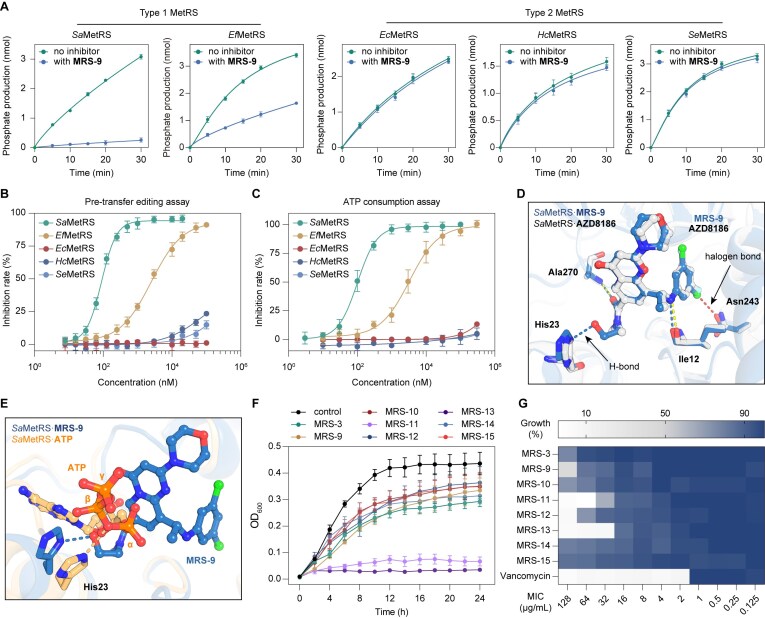
Activity evaluation and binding mode of **MRS-9**. (**A**) Phosphate production by type 1 and type 2 MetRSs in the presence or absence of **MRS-9** over time. The results are presented as mean ± SD (*n* = 3). (**B**) Enzymatic inhibition curves of **MRS-9** against type 1 and type 2 MetRSs, as determined by the pre-transfer editing assay. Each concentration was tested in triplicate, and data are presented as mean ± SD (*n* = 3). (**C**) Enzymatic inhibition curves of **MRS-9** against type 1 and type 2 MetRSs, as determined by the ATP consumption assay. Each concentration was tested in triplicate, and data are presented as mean ± SD (*n* = 3). (**D**) Comparison of the interactions between **MRS-9** and AZD8186 with *Sa*MetRS, highlighting the additional H-bonding and halogen bonding interactions formed by **MRS-9**. (**E**) The methanol moiety of **MRS-9** is located at the position corresponding to the binding site of the α-phosphate of ATP and forms an H-bond with His23. (**F**) Time-growth curves of *S. aureus* treated with **MRS-3** and its AI-assisted optimized derivatives **MRS-9** to **MRS-15**. The results are presented as mean ± SD (*n* = 3). (**G**) MIC values of **MRS-3** and its AI-assisted optimized derivatives **MRS-9** to **MRS-15** against *S. aureus* strain ATCC25923. The results are presented as mean ± SD (*n* = 3).

Inhibitors that interact with conserved residues, such as MetSA, frequently exhibit limited species selectivity [13]. Notably, utilizing pre-transfer editing assay, **MRS-9** demonstrated potent inhibition of type 1 *Sa*MetRS and *Ef*MetRS at a concentration of 5 μM, while exhibiting no detectable inhibition against the tested type 2 MetRSs, including *Ec*MetRS, *Hc*MetRS, and *Se*MetRS at the same concentration (Fig. 7A). Quantitative analysis revealed that **MRS-9** inhibited *Sa*MetRS and *Ef*MetRS with IC_50_ values of 0.09 ± 0.01 μM and 2.5 ± 0.3 μM, respectively. In contrast, its inhibitory rates against the three type 2 MetRSs did not exceed 25% even at 100 μM (Fig. 7B), indicating a selectivity of >1000-fold for **MRS-9** between *Sa*MetRS and *Hc*MetRS. The selectivity of **MRS-9** was further confirmed by the ATP consumption assay. In this assay, **MRS-9** inhibited *Sa*MetRS and *Ef*MetRS with IC_50_ values of 0.10 ± 0.01 μM and 3.3 ± 0.7 μM, respectively, while its inhibition against the three type 2 MetRSs remained below 25% even at 300 μM (Fig. 7C).

The co-crystal structure of the *Sa*MetRS·**MRS-9** complex was resolved at a 2.0 Å resolution (Supplementary Fig. S2C and Supplementary Table S1). Consistent with expectations, **MRS-9** exhibited a triple-site binding mode similar to that of AZD8186 (Supplementary Fig. S12A and B). Structural alignment of the *Sa*MetRS·**MRS-9** complex with the *Sa*MetRS·AZD8186 complex indicated that the dichlorophenyl group of **MRS-9** establishes a new halogen bond with Asn243 located in the l-Met binding site of *Sa*MetRS and the additional methanol moiety of **MRS-9** forms an H-bond with the conserved residue His23 (Fig. 7D and Supplementary Fig. S12C), thereby elucidating the molecular basis for the approximately 300-fold activity enhancement of **MRS-9** relative to AZD8186 in *Sa*MetRS inhibition. It is noteworthy that His23 is the fourth residue within the conserved “HIGH” motif, one of the two signature motifs for Class I aaRSs which is known to be implicated in multiple ways of catalysis, including amino acid activation and the acyl-transfer to tRNA [58]. To interact with His23, the additional methanol moiety of **MRS-9** occupies the position corresponding to the α-phosphate group of the substrate ATP (Fig. 7E).

Notably, several amino acid residues surrounding the **MRS-9** binding pocket differ between *Sa*MetRS and *Hc*MetRS (Supplementary Fig. S13A). Homology modeling of *Hc*MetRS, using the **MRS-9**-bound *Sa*MetRS structure as a template, revealed that **MRS-9** encounters severe steric clashes with *Hc*MetRS (Supplementary Fig. S13B). For example, Ile273 in *Sa*MetRS, which forms a critical hydrophobic interaction with the core structure of **MRS-9**, is replaced by the hydrophilic residue Asn556 in *Hc*MetRS, thereby abolishing this interaction and introducing steric hindrance. Additionally, Thr133 in *Sa*MetRS is replaced by Arg394 in *Hc*MetRS, whose bulky side chain clashes with the morpholine ring of **MRS-9** (Supplementary Fig. S13B). These observations suggest that the unique triple-site binding mode of **MRS-9** may be exclusively accommodated by type 1 MetRSs, but not by type 2 MetRSs, such as *Hc*MetRS.

The potential antibacterial activities of **MRS-3** and its AI-assisted optimized derivatives (**MRS-9** to **MRS-15**) were evaluated against *S. aureus* strain ATCC25923. At a concentration of 64 μg/ml, all compounds delayed the growth of *S. aureus*, with **MRS-11** and **MRS-13** completely inhibiting its proliferation. Subsequently, the MIC values of the compounds were measured using a series of 2-fold dilution concentrations up to 128 μg/ml; higher concentrations were not tested due to solubility limitations. **MRS-13** demonstrated an moderate antibacterial activity, with an MIC of 32 μg/ml. Furthermore, MIC values of 64 and 128 μg/ml were observed for **MRS-11** and **MRS-12**, respectively. However, **MRS-9**, the most potent enzymatic inhibitor, showed limited antibacterial cellular efficacy, with approximately 50% inhibition of *S. aureus* growth at 128 μg/ml. Notably, **MRS-9** is the *N*-methylated derivative of **MRS-12**, and **MRS-11** is the *N*-methylated derivative of **MRS-13**. Thus, although the two *N*-methylated derivatives exhibited better enzymatic inhibitory activity, the non-*N*-methylated compounds exhibited stronger antibacterial effects, indicating that beyond the enzymatic inhibitory potency, the physicochemical properties of the triple-site inhibitors significantly influence their antibacterial activity. To evaluate whether the antibacterial activity of these compounds is specific to type 1 MetRS at the organismal level, we selected *E. coli* (ATCC25922) as a representative organism carrying type 2 MetRS and determined the MIC values. The results showed that none of the tested compounds exhibited significant antibacterial activity against *E. coli*, with all MIC values >128 μg/ml (Supplementary Fig. S14). These findings further support that the antibacterial activity of these compounds is specific to type 1 MetRS.

## Discussion

Type 1 MetRS has long been recognized by scientists in both the pharmaceutical industry and academic institutions as a promising drug target for combating the infectious diseases caused by Gram-positive bacteria and parasites. Extensive efforts have been made to discover effective MetRS inhibitors [6–10], but most of these are l-Met-auxiliary dual-site inhibitors, and none of them has advanced to clinical use so far. There is an urgent need to develop novel inhibitors with alternative scaffolds and binding modes to advance the development of type 1 MetRS-based antibacterial agents. Given that both kinases and MetRS utilize ATP as a substrate, we screened a kinase inhibitor library against *Sa*MetRS and identified AZD8186, a PI3K inhibitor currently in phase II clinic trials, as a novel hit compound of interest for further optimization. To our surprise, although AZD8186 is an ATP-competitive inhibitor of PI3K [59], structural analyses suggested that it may exhibit a previously unobserved triple-site binding mode for MetRS. Notably, such a triple-site inhibitory mechanism has been reported previously for Class II aaRS members, such as Borrelidin and 36j for ThrRS, and MAT436 for ProRS [60–62]. The catalytic domains of Class I and II aaRSs differ in structural fold, the scaffolds of these inhibitors cannot applied to develop triple-site inhibitor for MetRS or other Class I aaRS members.

Upon determining the co-crystal structure of the *Sa*MetRS·AZD8186 complex, structure-based lead optimization was initially conducted using the established knowledge and experience in medicinal chemistry. This process led to the design of **MRS-3**, a compound featuring a more synthetically accessible scaffold and improved inhibitory activity due to enhanced interactions with the l-Met binding site. However, attempts to further extend **MRS-3** toward the conserved adenine-binding site through structure-guided linking of aromatic fragments were unsuccessful. Following the resolution of the co-crystal structures of the *Sa*MetRS·**MRS-3** and *Sa*MetRS·**MRS-9** complexes, we identified a novel ATP-site closure caused by the flipping of residue Trp301 (Supplementary Fig. S15). This closed conformation was not observed in the initial *Sa*MetRS·AZD8186 complex nor in any other reported MetRS structures (PDB codes: 7WPJ, 7WPL, 7WPK, 7WPI, 8XM4, 4EG3, and the AlphaFold 3 model), where Trp301 exhibited either poor electron density or an open conformation. Since there is no direct interaction between Trp301 and the compounds, it remains unclear how the scaffold hopping from the benzopyranone core of AZD8186 to the pyrido[1,2-a]pyrimidine scaffold of **MRS-3** could induce the flipping of Trp301. The closure of Trp301 may interfere with the binding of the adenine moiety of ATP and MetSA (Supplementary Fig. S8), which partially explains why the knowledge-guided linking of adenine-mimicking groups to **MRS-3** resulted in a dramatic decrease of inhibitory activity for **MRS-6** to **MRS-8** against *Sa*MetRS (Supplementary Fig. S6).

Further optimization of **MRS-3** was achieved through the development of a conservation-aware generative model, DiffDeCIG, designed to guide scaffold decoration for interactions with conserved residues in the binding pocket. Building upon our previous 3D target structure-guided molecular scaffold decoration model, DiffDec, DiffDeCIG further integrates the protein pocket conservation and protein–ligand interaction information into the diffusion model to enhance lead optimization. DiffDeCIG facilitated the identification of the compound **MRS-9**, a potent inhibitor against *Sa*MetRS with *K*_d_ = 38 nM and IC_50_ = 0.09 ± 0.01 µM. Importantly, DiffDeCIG is a general AI model that can be applied for structure-based lead optimization across a wide range of protein targets, and the successful discovery of **MRS-9** provides a practical demonstration for its effectiveness. Notably, although DiffDeCIG succeeded in enhancing molecular binding to the target, the contrasting activities of *N*-methylated and non-*N*-methylated analogs (**MRS-9** and **MRS-11** versus **MRS-12** and **MRS-13**) in enzyme and bacterial inhibition highlight that factor beyond enzyme inhibition, such as physicochemical properties affecting membrane penetration, also play a crucial role in antibacterial activity. This knowledge should to be also incorporated into future AI models for drug design.

## Supplementary Material

gkag488_Supplemental_File

## Data Availability

The authors declare that the main data supporting the findings of this study are available within the article, its Supplementary Information files. Extra data (raw plate reader data for biological experiments; NMR and LC/MS data files) are available from the corresponding author upon request. Images of the ^1^H and ^13^C NMR spectra are provided in the Supplementary Information. The co-crystal structures have been deposited in the Protein Data Bank (PDB) under the accession codes of 9V9D (https://doi.org/10.2210/pdb9v9d/pdb), 9V9F (https://doi.org/10.2210/pdb9v9f/pdb), and 9V9M (https://doi.org/10.2210/pdb9v9m/pdb). The code and datasets for DiffDeCIG are available at these repositories: https://github.com/anjie-qiao/DiffDeCIG and https://doi.org/10.5281/zenodo.19680019.
