# Supplementary material for: Discovery of a triple-site inhibitor targeting bacterial methionyl-tRNA synthetase through combined drug repurposing screening and generative AI-assisted optimization
Source: Nucleic Acids Res. 2026 May 19;54(9):gkag488. doi: 10.1093/nar/gkag488 (PMC13183674; doi:10.1093/nar/gkag488)
Supplement: gkag488_Supplemental_File [file gkag488_supplemental_file.pdf]

Supporting Information

Discovery of a triple-site inhibitor targeting bacterial methionyl-tRNA synthetase through combined drug repurposing screening and generative AI-assisted optimization

Jingtian Su[1,2,#], Anjie Qiao[3,#], Weifeng Huang[1,2], Jingyi Xu[1,2], Feihu Lu[1,2], Hao Zhang[1,2], Qirui Deng[1,2], Jialin Zou[3], Zhen Wang[3,*], Jinping Lei[1,2,*], Huihao Zhou[1,2,*]

[1]State Key Laboratory of Anti-Infective Drug Discovery and Development, School of Pharmaceutical Sciences, Sun Yat-sen University, Guangzhou 510006, China.

[2]Guangdong Provincial Key Laboratory of Chiral Molecule and Drug Discovery, School of Pharmaceutical Sciences, Sun Yat-sen University, Guangzhou 510006, China.

[3]School of Computer Science and Engineering, Sun Yat-sen University, Guangzhou 510006, China.

[#]These authors contributed equally to this research.

[*]Corresponding author: zhuihao@mail.sysu.edu.cn (H.Z.), leijp@mail.sysu.edu.cn (J.L.), wangzh665@mail.sysu.edu.cn (Z.W.)

Table of Contents:

Supplementary Methods

Development of the conservation-aware model 3

Chemical synthesis 7

Supplementary Figures

Supplementary Figure S1 14

Supplementary Figure S2 15

Supplementary Figure S3 16

Supplementary Figure S4 17

Supplementary Figure S5 19

Supplementary Figure S6 20

Supplementary Figure S7 21

Supplementary Figure S8 22

Supplementary Figure S9 23

Supplementary Figure S10 24

Supplementary Figure S11 25

Supplementary Figure S12 26

Supplementary Figure S13 27

Supplementary Figure S14 28

Supplementary Figure S15 29

Supplementary Tables

Supplementary Table S1 30

Supplementary Table S2 31

Supplementary Table S3 32

Supplementary NMR Spectra and Analytical Data

^{1}H NMR, ^{13}C NMR and HRMS dates of compounds 33

Supplementary References

References 54

Supplementary Methods

Development of the conservation-aware generative model

Dataset Construction

In this study, we focus on single R-group decoration-based molecule generation, where the goal is to generate an R-group that attaches to a predefined molecular scaffold. To achieve this, we conduct our experiments using the CrossDocked data set[1] that contains 22.5 million docked binding complexes and adopt the same preparation and reaction-based slicing method as DiffDec[2]. At first, we selected high-quality docking poses (RMSD < 1Å) and ensured protein diversity (sequence identity < 30%). Next, we extracted the protein substructure within a 10Å radius of the binding ligand. Following the splitting settings proposed by Luo et al.[3], the original training and test sets consisted of 100,000 and 100 protein-ligand pairs, respectively. Each ligand in the original training and test sets was split into a scaffold and an R-group using the slicing method from LibINVENT[4], which enhances validity and synthetic accessibility by applying 37 customized reaction-based rules. This process yielded a final training set of 76,370 tuples and a test set of 49 tuples, with each tuple comprising a protein pocket, a scaffold, and an R-group.

Preliminary

Given a protein pocket $\mathcal{P} = \{(x_i^{\mathcal{P}}, v_i^{\mathcal{P}})\}_{i=1}^{N_P}$ and a ligand molecule $\mathcal{M} = (\mathcal{S}, \mathcal{R})$, in this work, a ligand molecule $\mathcal{M}$ can be represented as a scaffold $\mathcal{S} = \{(x_i^{\mathcal{S}}, v_i^{\mathcal{S}})\}_{i=1}^{N_S}$ and an R-group $\mathcal{R} = \{(x_i^{\mathcal{R}}, v_i^{\mathcal{R}})\}_{i=1}^{N_R}$. Where N_P (resp. N_S, N_R) refers to the number of atoms of protein pocket $\mathcal{P}$ (resp. the scaffold $\mathcal{S}$, the R-group $\mathcal{R}$). $x \in \mathbb{R}^3$ and $v \in \mathbb{R}^K$ denote the 3D coordinate and type of the atom, respectively, where K denotes the number of atom types with v being one-hot vector. Throughout this work, matrices are represented by uppercase boldface letters. For a given matrix $\boldsymbol{X}$, x_i denotes the vector in its i-th row, For brevity, we denote the pocket as $\boldsymbol{P} = [\boldsymbol{X}^{\mathcal{P}}, \boldsymbol{V}^{\mathcal{P}}]$, where $\boldsymbol{X}^{\mathcal{P}} \in \mathbb{R}^{N_P \times 3}$ and $\boldsymbol{V}^{\mathcal{P}} \in \mathbb{R}^{N_P \times K}$. Similarly, the scaffold and R-group are represented as $\boldsymbol{S} = [\boldsymbol{X}^{\mathcal{S}}, \boldsymbol{V}^{\mathcal{S}}]$, $\boldsymbol{R} = [\boldsymbol{X}^{\mathcal{R}}, \boldsymbol{V}^{\mathcal{R}}]$, respectively. The decoration task can be formulated as modeling the conditional distribution $p(R|P, S)$, where, for simplicity, our notation does not distinguish the random variables from their realizations (i.e., specific matrices).

We proposed a conservation- and interaction-guided diffusion model to generate an R-group that is expected to bind to the highly conserved regions of the given pocket while enhancing interaction within the complex. Our framework extends the diffusion model with two key mechanisms: (1) conservation-aware conditioning pocket, which encourages the R-group to grow toward highly conserved region of the binding pocket, and (2) Interaction-prior guidance, which promotes the formation of stronger and more diverse interactions between the decorated ligand and the pocket.

Conservation-Aware Condition

For each protein pocket $\mathbf{P} = [\mathbf{X}^{\mathcal{P}}, \mathbf{V}^{\mathcal{P}}]$, we first retrieve its full amino acid sequence using its UniProt ID[5]. We then compute sequence conservation by performing multiple sequence alignments (MSAs) against the UniRef30_2023_02 database[6] through HHblits[7], a fast and sensitive homology detection tool based on Hidden Markov Models (HMMs). This process yields a conservation score matrix $\mathbf{C}^{\mathcal{P}}$ for the pocket P, where $\mathbf{C}^{\mathcal{P}} \in \mathbb{R}^{N_P \times 1}$ and each score $c_i \in [0,1]$, in which a larger value indicates the i-th amino acid residue with higher conservation. The conservation scores serve as additional features for the pocket representation, resulting in an augmented pocket descriptor:

$$\mathbf{P}_{\mathbf{CA}} = [\mathbf{X}^{\mathcal{P}}, \mathbf{V}^{\mathcal{P}}, \mathbf{C}^{\mathcal{P}}]$$

Interaction-Prior Guidance

We leverage IPNet[8] to offer interaction-based prior to our diffusion model. Specifically, IPNet, denoted as $\psi_{IPNet}(\cdot)$, is a pretrained interaction-prior network built upon SE(3)-equivariant neural networks[9] and cross-attention layers[10] to predict the binding affinity of protein-ligand complexes.

Given a protein $\boldsymbol{P}$ and a ligand molecule $\boldsymbol{M}$, IPNet encodes their interactions into protein and ligand representations, capturing the complex interplay between protein binding sites and ligand molecules:

$$F^{\mathcal{P}}, F^{\mathcal{M}} = \psi_{IPNet}(\boldsymbol{P}, \boldsymbol{M})$$

This representation is then utilized to predict the binding affinity of the protein-ligand complex. In our work, we repurpose this representation as an interaction prior to guide the diffusion process. Specifically, given a pocket $\boldsymbol{P}$, a scaffold $\boldsymbol{S}$, and an R-group $\boldsymbol{R}$, we extract their interaction-prior representations as:

$$F^{\mathcal{P}}, F^{\mathcal{S}}, F^{\mathcal{R}} = \psi_{IPNet}(\boldsymbol{P}, \boldsymbol{S}, \boldsymbol{R})$$

Next, we incorporate the Conservation-Aware and Interaction-Prior mechanisms to guide both the forward diffusion and reverse denoising stages for molecule generation.

Forward Diffusion Process

Recent studies often adopt fixed form noise schedule for diffusion-based generative models. In our work, we employ a variance-preserving cosine schedule version[11,12], which defines a set of β_t $(t = 0, \ldots, T)$ for each timestep t. Rather than directly applying this schedule, we redefine the noise process following the Signal-to-Noise Ratio (SNR)[13]:

$$\gamma_t = log(1 - \beta_t) - log\beta_t,$$
$$\alpha_t = \sqrt{sigmoid(-\gamma_t)},$$
$$\sigma_t = \sqrt{sigmoid(\gamma_t)}.$$

In the forward process, we model the atomic coordinates and types of R-group $\boldsymbol{R}$ as continuous random variables and introduce noise iteratively from a Gaussian distribution $\mathcal{N}(0, I)$ at each time step t. The scaffold S and pocket P are treated as fixed contextual information, remaining unchanged

throughout both the forward and reverse processes:

$$q(R_t|R_0, S, P) = \mathcal{N}(R_t; \alpha_t R_0, \sigma_t^2 I).$$

Moreover, we extract the interactive representations of R-group $F_0^{\mathcal{R}}$ from the pretrained IPNet $\psi_{INPnet}(R_0, S, P)$, and then introduce a learnable neural network $\psi_\theta(\cdot)$ for guiding the R-group atom coordinates generation with an interaction-based shifting:

$$\boldsymbol{S}_t^{\mathcal{R}} = k_t \cdot \psi_\theta(F^{\mathcal{R}}, t),$$

where $\psi_\theta(\cdot)$ is a MLP neural network, $\boldsymbol{S}_t^{\mathcal{R}} \in \mathbb{R}^{N_R \times 3}$ is the cumulative mean shift in step t, and k_t is a pre-defined preserving scaling coefficient:

$$k_t = \sqrt{\prod_{s=1}^{t}(1-\beta_s)} \cdot \left(1 - \sqrt{\prod_{s=1}^{t}(1-\beta_s)}\right).$$

We incorporate this mean shift into the forward diffusion process as follows:

$$q\left(R_t \middle| R_0, S, P, F_0^{\mathcal{R}}\right) = \mathcal{N}(R_t; \alpha_t R_0 + S_t^R, \sigma_t^2 I),$$

$$q\left(R_t \middle| R_{t-1}, S, P, F_0^{\mathcal{R}}\right) = \mathcal{N}\left(R_t; \alpha_{t|t-1}(R_{t-1} - S_{t-1}^R) + S_t^R, \sigma_{t|t-1}^2 I\right),$$

where

$$\alpha_{t|t-1} = \alpha_t / \alpha_{t-1},$$

$$\sigma_{t|t-1}^2 = \sigma_t^2 - \alpha_{t|t-1}^2 \sigma_{t-1}^2.$$

Reverse denoising process

In the reverse process, our objective is to learn to reverse the forward noise injection. The corresponding posterior can be expressed as:

$$q(R_{t-1}|R_t, S, P) = \mathcal{N}(R_{t-1}; \tilde{\mu}(R_t, R_0, t), \tilde{\sigma}(t) I),$$

where

$$\tilde{\mu}(R_t, R_0, t) = \frac{\alpha_{t|t-1}\sigma_{t-1}^2}{\sigma_t^2} R_t + \frac{\alpha_{t-1}\sigma_{t|t-1}^2}{\sigma_t^2} R_0,$$

$$\tilde{\sigma}(t) = \sigma_{t|t-1}^2 \sigma_{t-1}^2 / \sigma_t^2.$$

However, since the ground-truth fragment R_0 is inaccessible at timestep t, we train a denoiser $\varphi_\theta(\cdot)$ to approximate it. Specifically, we use this denoiser to predict the Gaussian noise and concurrently replace P with the conservation-augmented pocket P_{CA} during noise prediction:

$$\hat{\epsilon}_t = \varphi_\theta(R_t, S, P_{CA}, t),$$

The training objective is to minimize the mean squared error between true noise ϵ and the predicted noise $\hat{\epsilon}_t$:

$$\mathcal{L} = ||\epsilon - \hat{\epsilon}_t||^2.$$

From the predicted noise, we obtain an estimate of the ground-truth:

$$\hat{R}_0 = (1/\alpha_t) R_t - (\sigma_t/\alpha_t)\varphi_\theta(R_t, S, P_{CA}, t).$$

Since $\boldsymbol{R_0}$ is not directly available, we use the estimated $\hat{R}_0$ from the previous time step $t+1$ to compute the interactive representation:

$$F_{t+1}^{\mathcal{R}}, F_{t+1}^{\mathcal{P}}, F_{t+1}^{\mathcal{S}} = \psi_{IPNet}\left(P, S, \hat{R}_0^{t+1}\right),$$

which are then used to calculate the mean shift for the R-group:

$$S_t^{\mathcal{R}} = k_t \cdot \psi_\theta\left(F_{t+1}^{\mathcal{R}}, t\right).$$

Similarly, the estimate $\hat{R}_0^t$ obtained at timestep t is used to compute $S_{t-1}^{\mathcal{R}}$ at next timestep $t-1$. At the beginning timestep $t = T$, the initial interactive representation $F_{T+1}^{\mathcal{R}}$ is 'None'.

To maximize the exploitation of the protein-ligand interaction prior encoded in the pre-trained IPNet, we further integrate the interactive representation $F_{t+1}^{\mathcal{P}}$, $F_{t+1}^{\mathcal{S}}$, $F_{t+1}^{\mathcal{R}}$ into the denoiser prediction:

$$\hat{R}_0 = (1/\alpha_t)R_t - (\sigma_t/\alpha_t)\varphi_\theta\left(R_t, S, P_{CA}, F_{t+1}^{\mathcal{P}}, F_{t+1}^{\mathcal{S}}, F_{t+1}^{\mathcal{R}}, t\right).$$

Therefore, the reverse transition kernel is then formulated as:

$$p\left(R_{t-1} \middle| R_t, S, P, F_{t+1}^{\mathcal{R}}, F_{t+1}^{\mathcal{P}}, F_{t+1}^{\mathcal{S}}\right) = \mathcal{N}\left(R_{t-1}; \tilde{\mu}\left(R_t, \hat{R}_0, F_{t+1}^{\mathcal{R}}, F_{t+1}^{\mathcal{P}}, F_{t+1}^{\mathcal{S}}, t\right), \tilde{\sigma}(t)I\right),$$

where

$$\tilde{\mu}\left(R_t, R_0, F_{t+1}^{\mathcal{R}}, F_{t+1}^{\mathcal{P}}, F_{t+1}^{\mathcal{S}}, t\right) = \frac{\alpha_{t|t-1}\sigma_{t-1}^2}{\sigma_t^2}\left(R_t - S_t^{\mathcal{R}}\right) + \frac{\alpha_{t-1}\sigma_{t|t-1}^2}{\sigma_t^2}\hat{R}_0 + S_{t-1}^{\mathcal{R}}.$$

In this way, by integrating both the Conservation-Aware and Interaction-Prior mechanisms, our diffusion model leverages evolutionary information and protein-ligand interaction cues to guide the reverse process to recover the underlying molecular configuration.

Chemical synthesis

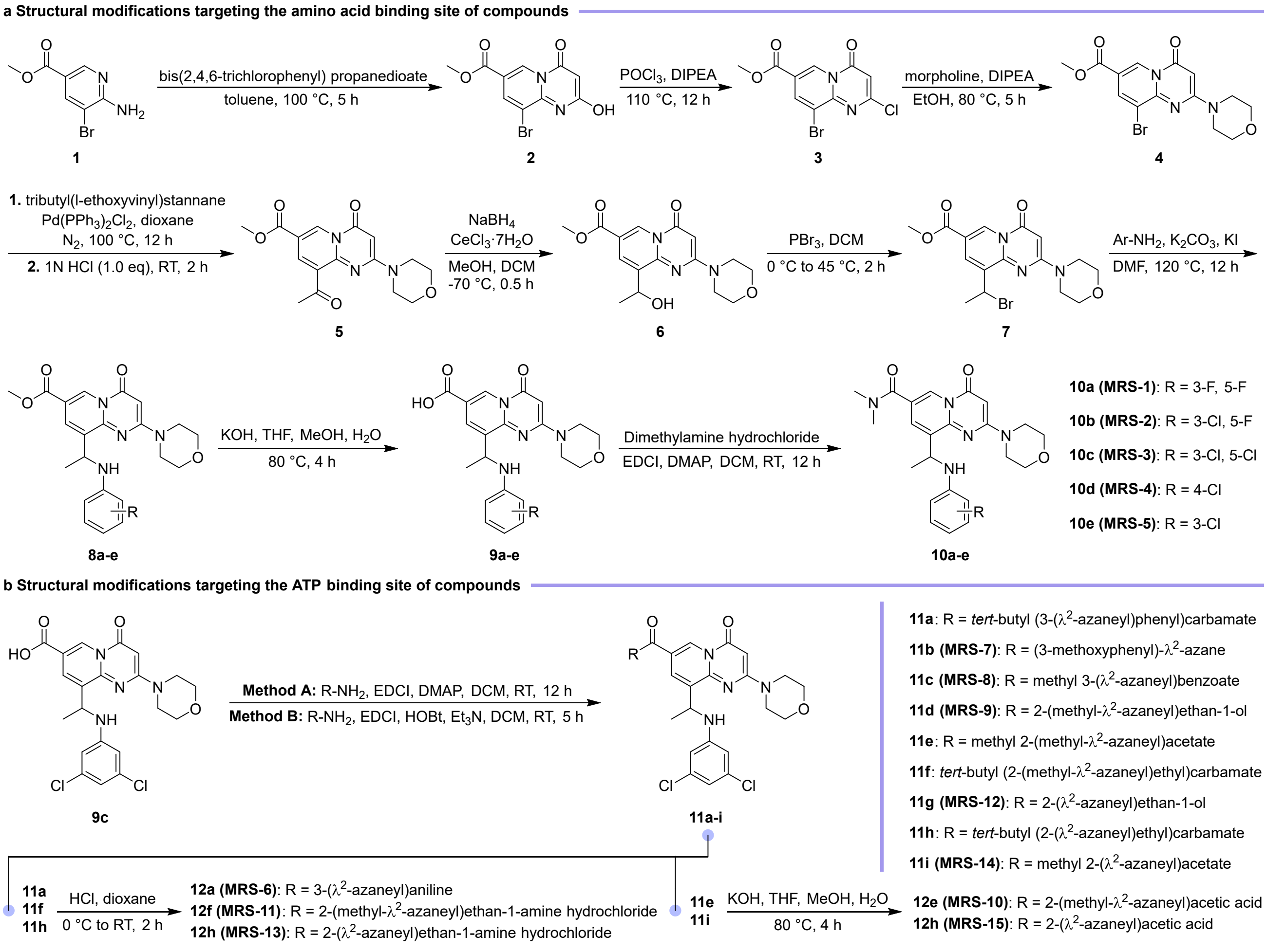

Synthesis route of compounds

Methyl 9-bromo-2-hydroxy-4-oxo-4H-pyrido[1,2-a]pyrimidine-7-carboxylate **(2)**

To a mixture of methyl 6-amino-5-bromonicotinate (**1**, 7.5 g, 32.5 mmol) in toluene (120 mL) was added bis(2,4,6-trichlorophenyl) propanedioate (18 g, 39 mmol, 1.2 *eq*) in portions at 25 °C. The mixture was heated to 100 °C and stirred for 5 h under N_2 atmosphere. TLC showed the reaction was complete. After cooling to room temperature, the precipitate was collected by vacuum filtration, and washed with cold petroleum ether until the filtrate was colourless. The precipitate was dried in vacuo to afford compound **2** (8.92 g, 29.9 mmol, 92%) as light brown solid. ^{1}H NMR (400 MHz, DMSO-d_6) δ 12.24 (s, 1H), 9.34 (d, *J* = 1.9 Hz, 1H), 8.48 (d, *J* = 2.0 Hz, 1H), 5.60 (s, 1H), 3.93 (s, 3H). MS (ESI): *m/z* $[M+H]^+$ = 298.95.

Methyl 9-bromo-2-chloro-4-oxo-4H-pyrido[1,2-a]pyrimidine-7-carboxylate **(3)**

To a solution of **2** (8.8 g, 29.9 mmol) in $POCl_3$ (80 mL) was added dropwise DIPEA (5.2 mL, 29.9 mmol, 1.0 *eq*) at 0 °C. The mixture was heated to 110 °C and stirred for 12 h under N_2 atmosphere. After cooling to room temperature, the reaction mixture was concentrated under reduced pressure to give a residue. To the residue was added dropwise H_2O at 0 °C until no soild was produced. The precipitate

was collected by vacuum filtration, and washed with cold H_2O until the filtrate was colourless. The precipitate was dried in vacuo to afford compound **3** (9.5 g, 30.1 mmol, 93%) as grey brown solid, which was used into the next step without further purification. This analysis pure compound **3** was further purified as yellow soild by column chromatography. ^1H NMR (400 MHz, DMSO-d_6) δ 9.33 (d, *J* = 1.9 Hz, 1H), 8.64 (d, *J* = 1.9 Hz, 1H), 6.75 (s, 1H), 3.96 (s, 3H). MS (ESI): *m/z* $[M+H]^+$ = 318.35.

Methyl 9-bromo-2-morpholino-4-oxo-4H-pyrido[1,2-a]pyrimidine-7-carboxylate **(4)**

To a mixture of **3** (12.6 g, 39.9 mmol) and DIPEA (13.9 mL, 79.8 mmol, 2.0 *eq*) in EtOH (200 mL) was added dropwise morpholine (5.2 mL, 59.9 mmol, 1.5 *eq*) at 25 °C. The mixture was heated to 80 °C and stirred for 5 h under N_2 atmosphere. After cooling to room temperature, the precipitate was collected by vacuum filtration, and washed with cold EtOH until the filtrate was colourless. The precipitate was dried in vacuo to afford compound **4** (14.4 g, 39.2 mmol, 98%) as light yellow solid. ^1H NMR (400 MHz, Chloroform-*d*) δ 9.54 (d, *J* = 1.8 Hz, 1H), 8.43 (d, *J* = 1.8 Hz, 1H), 5.57 (s, 1H), 3.96 (s, 3H), 3.84 – 3.70 (m, 8H). MS (ESI): *m/z* $[M+H]^+$ = 368.00.

Methyl 9-acetyl-2-morpholino-4-oxo-4H-pyrido[1,2-a]pyrimidine-7-carboxylate **(5)**

A mixture of **4** (14 g, 38.1 mmol), tributyl(l-ethoxyvinyl)stannane (17.9 mL, 57.2 mmol, 1.5 *eq*), and $Pd(PPh_3)_2Cl_2$ (1.3 g, 1.9 mmol, 0.05 *eq*) in dioxane (150 mL) was stirred at 100 °C for 12 h under N_2. After cooling to room temperature, the mixture was added dropwise 1N HCl (38 mL, 38.1 mmol1.0 *eq*) and was stirred at 25 °C for 2 h. The organic solvent was removed under reduced pressure. The aqueous was adjusted to pH = 7 with saturated aqueous $NaHCO_3$ and extracted with DCM (3 × 100 mL). The combined organic phase was washed with brine (3 × 50 mL), dried over Na_2SO_4, filtered and concentrated under reduced pressure to give a residue. The residue was triturated by petroleum ether. The precipitate was collected by vacuum filtration, and washed with cold petroleum ether until the filtrate was colourless. The precipitate was further purified by column chromatography to afford compound **5** (10 g, 30.2 mmol, 79%) as yellow soild. ^1H NMR (400 MHz, Chloroform-*d*) δ 9.67 (d, *J* = 2.2 Hz, 1H), 8.39 (d, *J* = 2.2 Hz, 1H), 5.61 (s, 1H), 3.96 (s, 3H), 3.82 – 3.78 (m, 4H), 3.72 – 3.65 (m, 4H), 2.74 (s, 3H). MS (ESI): *m/z* $[M+H]^+$ = 332.15.

Methyl 9-(1-hydroxyethyl)-2-morpholino-4-oxo-4H-pyrido[1,2-a]pyrimidine-7-carboxylate **(6)**

To a mixture of **5** (5 g, 15.1 mmol) and $CeCl_3{\cdot}7H_2O$ (5.4 g, 15.1 mmol, 1 *eq*) in MeOH (60 mL) and DCM (15 mL) was added $NaBH_4$ (330 mg, 9.6 mmol, 0.6 eq) in portions at - 70 °C and then the mixture was stirred at -70 °C for 0.5 h. TLC showed the reaction was complete. The mixture was quenched with ice water slowly at -70 °C until no gas was produced. The mixture was concentrated under reduced pressure to give a residue. The residue was dissolved with MeOH, dried over Na_2SO_4, and filtered. The filtrate was further purified by column chromatography to afford compound **6** (3.6 g, 10.8 mmol, 71%) as light yellow soild. ^1H NMR (400 MHz, DMSO-d_6) δ 9.25 (d, *J* = 2.1 Hz, 1H), 8.18 (d, *J* = 2.1 Hz, 1H), 5.68 (s,

1H), 5.51 (d, J = 4.7 Hz, 1H), 5.18 (p, J = 5.7 Hz, 1H), 3.91 (s, 3H), 3.73 – 3.60 (m, 8H), 1.40 (d, J = 6.3 Hz, 3H). MS (ESI): *m/z* $[M+H]^+$ = 334.15.

Methyl 9-(1-bromoethyl)-2-morpholino-4-oxo-4H-pyrido[1,2-a]pyrimidine-7-carboxylate **(7)**

To a solution of **6** (3.6 g, 10.8 mmol) in DCM (30 mL) was added dropwise PBr_3 (1.3 mL, 14 mmol, 1.3 *eq*) at 0 °C. The mixture was heated to 45 °C and stirred for 2 h under N_2 atmosphere. After cooling to room temperature, the mixture was adjusted to pH = 7 with saturated aqueous $NaHCO_3$ and extracted with DCM (3 × 50 mL). The combined organic phase was washed with brine (3 × 25 mL), dried over Na_2SO_4, filtered and concentrated under reduced pressure to give a residue. The residue was triturated by EtOAc. The precipitate was collected by vacuum filtration, and washed with cold EtOAc until the filtrate was colourless. The precipitate was further purified by column chromatography to afford compound **7** (2.8 g, 7.1 mmol, 66%) as white soild. ^{1}H NMR (400 MHz, Chloroform-*d*) δ 9.56 (d, J = 2.0 Hz, 1H), 8.31 (d, J = 2.0 Hz, 1H), 5.82 (q, J = 7.0 Hz, 1H), 5.61 (s, 1H), 3.97 (s, 3H), 3.85 – 3.68 (m, 8H), 2.12 (d, J = 7.0 Hz, 3H). MS (ESI): *m/z* $[M+H]^+$ = 396.05.

General synthesis procedure for compounds **10a-e** (**MRS-1** to **MRS-5**)

As an example, a mixture of **7** (150 mg, 0.38 mmol), 3,5-dichloroaniline (62 mg, 0.38 mmol, 1 *eq*), K_2CO_3 (105mg, 0.76 mmol, 2 *eq*) and KI (6.7mg, 0.04 mmol, 0.1 *eq*) in DMF (5 mL) was stirred at 120 °C for 12 h under N_2. After cooling to room temperature, the reaction mixture was diluted with H_2O (30 mL) and extracted with EtOAc (3 × 30 mL). The combined organic phase was washed with brine (3 × 20 mL), dried over Na_2SO_4, filtered and concentrated under reduced pressure to give a residue. The residue was further purified by column chromatography to afford compound **8c** (85 mg, 0.18 mmol, 47%) as light pink soild. ^{1}H NMR (400 MHz, DMSO-d_6) δ 9.24 (d, J = 2.1 Hz, 1H), 8.02 (d, J = 2.1 Hz, 1H), 7.04 (d, J = 7.3 Hz, 1H), 6.61 (s, 1H), 6.46 (s, 2H), 5.73 (s, 1H), 5.10 (p, J = 6.7 Hz, 1H), 3.87 (s, 3H), 3.75 – 3.65 (m, 8H), 1.48 (d, J = 6.7 Hz, 3H).

A mixture of **8c** (80 mg, 0.17 mmol), KOH (34 mg, 0.85 mmol, 5 *eq*) in MeOH (2 mL), THF (2 mL), and H_2O (2 mL) was stirred at 80 °C for 4 h under N_2. After cooling to room temperature, the organic solvent was removed under reduced pressure. The aqueous was adjusted to pH = 4 with 1N HCl. The precipitate was collected by vacuum filtration, washed with cold H_2O, and dried in vacuo to afford compound **9c** (69 mg, 0.15 mmol, 90%) as white solid. ^{1}H NMR (400 MHz, DMSO-d_6) δ 9.23 (d, J = 2.1 Hz, 1H), 8.03 (d, J = 2.0 Hz, 1H), 7.04 (d, J = 7.0 Hz, 1H), 6.61 (s, 1H), 6.46 (s, 2H), 5.72 (s, 1H), 5.15 – 5.05 (m, 1H), 3.75 – 3.65 (m, 8H), 1.48 (d, J = 6.6 Hz, 3H).

A mixture of **9c** (50 mg, 0.11 mmol), dimethylamine hydrochloride (18 mg, 0.22 mmol, 2 *eq*), EDCI (42 mg, 0.22 mmol, 2 *eq*) and DMAP (27 mg, 0.22 mmol, 2 *eq*) in DCM (5 mL) was stirred at 25 °C for 12 h. The organic solvent was removed under reduced pressure. The residue was diluted with H_2O (20 mL) and extracted with EtOAc (3 × 20 mL). The combined organic phase was washed with 1N HCl (3 × 5 mL)

and brine (3 × 20 mL), dried over Na_2SO_4, filtered and concentrated under reduced pressure to give a residue. The residue was further purified by column chromatography to afford compound **10c** (**MRS-3**, 30 mg, 0.06 mmol, 55%) as white soild. ^{1}H NMR (400 MHz, DMSO-d_6) δ 8.77 (d, J = 2.0 Hz, 1H), 7.72 (d, J = 2.0 Hz, 1H), 6.91 (d, J = 7.3 Hz, 1H), 6.60 (s, 1H), 6.47 (s, 2H), 5.71 (s, 1H), 5.12 (p, J = 7.0 Hz, 1H), 3.72 – 3.66 (m, 8H), 2.93 (s, 6H), 1.50 (d, J = 6.6 Hz, 3H). ^{13}C NMR (101 MHz, DMSO) δ 166.83, 160.31, 157.83, 150.06, 148.37, 137.52, 134.81, 131.95, 126.15, 121.16, 115.34, 111.15, 80.61, 66.32, 47.45, 44.90, 21.49. MS (ESI): *m/z* [M+H]$^+$ = 490.10. HRMS (ESI): *m/z* calcd for C23H25Cl2N5O3 [M+H]$^+$ 490.1407, found 490.1411.

Compound **10a** (**MRS-1**), white soild. ^{1}H NMR (400 MHz, DMSO-d_6) δ 8.77 (d, J = 2.0 Hz, 1H), 7.70 (d, J = 2.1 Hz, 1H), 6.95 (d, J = 7.2 Hz, 1H), 6.26 – 6.18 (m, 1H), 6.15 – 6.06 (m, 2H), 5.70 (s, 1H), 5.09 (p, J = 6.8 Hz, 1H), 3.74 – 3.66 (m, 8H), 2.91 (s, 6H), 1.50 (d, J = 6.6 Hz, 3H). ^{13}C NMR (101 MHz, $CDCl_3$) δ 167.30, 165.32, 165.16, 162.89, 162.74, 160.28, 158.57, 148.80, 148.67, 148.54, 148.12, 137.06, 131.94, 126.31, 120.60, 96.06, 95.78, 93.07, 92.81, 92.55, 81.33, 66.48, 48.82, 44.65, 21.44. ^{19}F NMR (376 MHz, $CDCl_3$) δ -109.94. MS (ESI): *m/z* [M+H]$^+$ = 458.20. HRMS (ESI): *m/z* calcd for C23H25F2N5O3 [M+H]$^+$ 458.1998, found 458.2009.

Compound **10b** (**MRS-2**), yellow soild. ^{1}H NMR (400 MHz, DMSO-d_6) δ 8.80 (d, J = 16.8 Hz, 1H), 7.77 (d, J = 15.0 Hz, 1H), 6.98 (d, J = 6.6 Hz, 1H), 6.45 (d, J = 8.3 Hz, 2H), 6.22 (d, J = 11.3 Hz, 1H), 5.75 (s, 1H), 5.21 – 5.08 (m, 1H), 3.82 – 3.64 (m, 8H), 2.96 (s, 6H), 1.55 (d, J = 5.8 Hz, 3H). ^{13}C NMR (126 MHz, DMSO) δ 166.80, 164.54, 162.66, 160.25, 157.85, 150.41, 148.35, 137.54, 134.53, 131.80, 126.09, 121.05, 108.95, 103.35, 98.18, 80.57, 66.30, 55.36, 47.62, 44.85, 21.40. ^{19}F NMR (376 MHz, DMSO) δ -111.07. MS (ESI): *m/z* [M+H]$^+$ = 474.15. HRMS (ESI): *m/z* calcd for C23H25ClFN5O3 [M+H]$^+$ 474.1703, found 474.1682.

Compound **10d** (**MRS-4**), light yellow soild. ^{1}H NMR (400 MHz, Chloroform-d) δ 8.90 (d, J = 2.1 Hz, 1H), 7.67 (d, J = 2.1 Hz, 1H), 6.97 (d, J = 8.7 Hz, 2H), 6.30 (d, J = 8.7 Hz, 2H), 5.58 (s, 1H), 5.01 (q, J = 6.7 Hz, 1H), 3.77 – 3.56 (m, 8H), 2.92 (s, 6H), 1.52 (d, J = 6.7 Hz, 3H). ^{13}C NMR (101 MHz, DMSO) δ 166.90, 160.31, 157.88, 148.43, 146.56, 138.08, 131.61, 129.07, 125.92, 121.17, 119.94, 114.55, 80.57, 66.30, 47.86, 44.82, 21.54. MS (ESI): *m/z* [M+H]$^+$ = 456.20. HRMS (ESI): *m/z* calcd for C23H26ClN5O3 [M+H]$^+$ 456.1797, found 456.1809.

Compound **10e** (**MRS-5**), yellow soild. ^{1}H NMR (400 MHz, Chloroform-d) δ 8.99 (d, J = 2.1 Hz, 1H), 7.74 (d, J = 2.1 Hz, 1H), 7.00 (t, J = 8.0 Hz, 1H), 6.63 (d, J = 7.9 Hz, 1H), 6.50 – 6.41 (m, 1H), 6.32 (dd, J = 8.3, 2.2 Hz, 1H), 5.66 (s, 1H), 5.10 (q, J = 6.6 Hz, 1H), 3.84 – 3.63 (m, 8H), 3.00 (s, 6H), 1.59 (d, J = 6.7 Hz, 3H). ^{13}C NMR (101 MHz, DMSO) δ 166.88, 160.32, 157.86, 149.22, 148.40, 138.05, 133.95, 131.73, 130.95, 126.00, 121.19, 116.08, 112.57, 111.54, 80.59, 66.32, 47.58, 44.86, 21.57. MS (ESI): *m/z* [M+H]$^+$ = 456.15. HRMS (ESI): *m/z* calcd for C23H26ClN5O3 [M+H]$^+$ 456.1797, found 456.1800.

General synthesis procedure for compounds **MRS-6** to **MRS-15**

Method A. This method is equivalent to the *general synthesis procedure for compounds* ***10a–e*** *(**MRS-1** to **MRS-5**)*, with the only difference being the use of different amine substrates corresponding to each target compound. The majority of compounds **11a–i** were synthesized using Method A, with the exception of **11d** (**MRS-9**), **11f**, **11g** (**MRS-12**), and **11h**, for which **Method B** was employed.

Compound **MRS-7**, white soild. ^{1}H NMR (400 MHz, Chloroform-*d*) δ 10.43 (s, 1H), 9.98 (s, 1H), 8.35 (s, 1H), 7.50 (s, 1H), 7.36 – 7.27 (m, 2H), 6.72 (d, *J* = 7.8 Hz, 1H), 6.62 (s, 1H), 6.34 (s, 2H), 5.41 (s, 1H), 5.13 (q, *J* = 6.4 Hz, 1H), 3.83 – 3.69 (m, 8H), 3.52 (s, 3H), 1.57 (d, *J* = 6.6 Hz, 3H). ^{13}C NMR (101 MHz, DMSO) δ 162.95, 160.24, 159.85, 158.00, 150.11, 148.88, 140.33, 137.80, 134.84, 131.50, 129.84, 127.59, 120.21, 115.27, 113.21, 110.97, 109.94, 106.72, 80.55, 66.32, 55.44, 47.32, 44.84, 21.56. MS (ESI): *m/z* $[M-H]^-$ = 566.20. HRMS (ESI): *m/z* calcd for C28H27Cl2N5O4 $[M+H]^+$ 568.1513, found 568.1515.

Compound **MRS-8**, white soild. ^{1}H NMR (400 MHz, Chloroform-*d*) δ 10.71 (s, 1H), 9.99 (s, 1H), 8.41 (s, 1H), 8.35 (s, 1H), 8.05 (d, *J* = 8.0 Hz, 1H), 7.82 (d, *J* = 7.4 Hz, 1H), 7.44 (t, *J* = 7.9 Hz, 1H), 6.62 (s, 1H), 6.35 (s, 2H), 5.44 (s, 1H), 5.13 (q, *J* = 6.9 Hz, 1H), 3.94 – 3.85 (m, 4H), 3.78 – 3.71 (m, 4H), 3.55 (s, 3H), 1.59 (d, *J* = 6.3 Hz, 3H). ^{13}C NMR (101 MHz, DMSO) δ 166.47, 163.08, 160.24, 157.98, 150.13, 148.90, 139.61, 137.80, 134.84, 131.37, 130.47, 129.54, 127.87, 125.32, 124.92, 121.46, 119.84, 115.27, 110.96, 80.54, 66.32, 52.64, 47.34, 44.85, 21.53. MS (ESI): *m/z* $[M-H]^-$ = 594.20. HRMS (ESI): *m/z* calcd for C29H27Cl2N5O5 $[M+H]^+$ 596.1462, found 596.1462.

Compound **MRS-14**, white soild. ^{1}H NMR (400 MHz, DMSO-d_6) δ 9.41 – 9.32 (m, 2H), 8.09 (d, *J* = 1.9 Hz, 1H), 6.98 (d, *J* = 7.0 Hz, 1H), 6.59 (t, *J* = 1.6 Hz, 1H), 6.53 – 6.40 (m, 2H), 5.12 (p, *J* = 6.5 Hz, 1H), 4.01 (d, *J* = 5.7 Hz, 2H), 3.77 – 3.67 (m, 8H), 3.66 – 3.63 (m, 3H), 1.50 (d, *J* = 6.6 Hz, 3H). ^{13}C NMR (101 MHz, DMSO) δ 170.57, 164.36, 160.27, 157.93, 150.16, 148.92, 137.81, 134.82, 130.95, 127.32, 119.15, 115.26, 80.50, 66.31, 52.27, 47.38, 44.86, 41.69, 21.53. MS (ESI): *m/z* $[M+H]^+$ = 534.10. HRMS (ESI): *m/z* calcd for C24H25Cl2N5O5 $[M+H]^+$ 534.1306, found 534.1292.

Method B. A mixture of compound **9c** (80 mg, 0.18 mmol, 1.0 *eq*), the corresponding amine substrate (1.1 *eq*), EDCI (1.5 *eq*), HOBt (1.5 *eq*), and Et_3N (2.0 *eq*) in DCM (5 mL) was stirred at 25 °C for 5 h. The organic solvent was then removed under reduced pressure. The residue was diluted with H_2O (20 mL) and extracted with EtOAc (3 × 20 mL). The combined organic layers were washed with brine (3 × 20 mL), dried over anhydrous Na_2SO_4, filtered, and concentrated under reduced pressure to yield a residue. The residue was further purified by column chromatography to afford compounds **11d** (**MRS-9**), **11f**, **11g** (**MRS-12**), and **11h**.

Compound **MRS-9**, white soild. ^{1}H NMR (400 MHz, DMSO-d_6) δ 8.81 (s, 1H), 7.72 (s, 1H), 6.89 (d, *J* = 6.9 Hz, 1H), 6.60 (s, 1H), 6.46 (s, 2H), 5.70 (s, 1H), 5.16 – 5.06 (m, 1H), 4.75 (s, 1H), 3.74 – 3.63 (m, 8H), 3.60 – 3.34 (m, 4H), 2.95 (s, 3H), 1.50 (d, *J* = 6.6 Hz, 3H). ^{13}C NMR (101 MHz, DMSO) δ 160.31, 157.88, 150.07, 148.31, 137.49, 134.80, 132.17, 126.03, 115.31, 111.11, 80.62, 66.32, 47.46, 44.90,

21.50. MS (ESI): *m/z* $[M+H]^+$ = 520.15. HRMS (ESI): *m/z* calcd for C24H27Cl2N5O4 $[M+H]^+$ 520.1513, found 520.1506.

Compound **MRS-12**, white soild. ^1H NMR (400 MHz, DMSO-d_6) δ 9.30 (d, J = 1.8 Hz, 1H), 8.85 (t, J = 5.3 Hz, 1H), 8.10 (d, J = 1.8 Hz, 1H), 6.98 (d, J = 6.9 Hz, 1H), 6.59 (s, 1H), 6.45 (s, 2H), 5.72 (s, 1H), 5.16 – 5.08 (m, 1H), 4.72 (t, J = 5.6 Hz, 1H), 3.73 – 3.64 (m, 8H), 3.50 (q, J = 6.0 Hz, 2H), 3.30 – 3.26 (m, 2H), 1.49 (d, J = 6.6 Hz, 3H). ^{13}C NMR (101 MHz, DMSO) δ 163.92, 160.26, 158.00, 150.17, 148.83, 137.59, 134.82, 131.39, 126.97, 120.06, 115.22, 110.94, 80.49, 66.32, 59.99, 47.28, 44.84, 42.75, 21.60. MS (ESI): *m/z* $[M+H]^+$ = 506.15. HRMS (ESI): *m/z* calcd for C23H25Cl2N5O4 $[M+H]^+$ 506.1356, found 506.1361.

To obtain compounds **MRS-6**, **MRS-11**, and **MRS-13**, compounds **11a**, **11f** and **11h**, respectively, were each dissolved in 2 mL of 4 M HCl in dioxane and added dropwise at 0 °C. The mixtures were then allowed to warm to room temperature and stirred for 2 hours. The resulting precipitates were collected by filtration, washed several times with cold dioxane, and dried under vacuum to yield **MRS-6**, **MRS-11**, and **MRS-13** as solid products.

Compound **MRS-6**, white soild. ^1H NMR (400 MHz, DMSO-d_6) δ 10.38 (s, 1H), 9.41 (s, 1H), 8.14 (s, 1H), 7.13 – 6.93 (m, 3H), 6.85 (d, J = 7.7 Hz, 1H), 6.60 (s, 1H), 6.48 (s, 2H), 6.34 (d, J = 7.5 Hz, 1H), 5.25 – 5.05 (m, 3H), 3.94 – 3.63 (m, 8H), 1.52 (d, J = 6.2 Hz, 3H). ^{13}C NMR (101 MHz, DMSO) δ 162.69, 160.26, 157.98, 150.16, 149.34, 148.86, 139.69, 137.68, 134.84, 131.65, 129.32, 127.46, 120.55, 115.27, 110.98, 110.59, 109.00, 106.75, 80.51, 66.32, 47.27, 44.86, 21.58. MS (ESI): *m/z* $[M+H]^+$ = 553.15. HRMS (ESI): *m/z* calcd for C27H26Cl2N6O3 $[M+H]^+$ 553.1516, found 553.1525.

Compound **MRS-11**, white soild. ^1H NMR (400 MHz, DMSO-d_6) δ 8.94 (s, 1H), 8.02 (s, 3H), 7.86 (s, 1H), 7.03 (s, 1H), 6.60 (t, J = 1.7 Hz, 1H), 6.54 – 6.46 (m, 2H), 5.72 (s, 1H), 5.17 – 5.07 (m, 1H), 3.75 – 3.62 (m, 10H), 3.12 – 2.99 (m, 2H), 2.93 (s, 3H), 1.51 (d, J = 6.6 Hz, 3H). MS (ESI): *m/z* $[M+H]^+$ = 519.10.

Compound **MRS-13**, yellow soild. ^1H NMR (400 MHz, DMSO-d_6) δ 9.31 (d, J = 1.7 Hz, 1H), 9.06 (t, J = 5.2 Hz, 1H), 8.33 – 8.24 (m, 1H), 8.12 (s, 3H), 7.07 (s, 1H), 6.60 (d, J = 21.2 Hz, 1H), 6.50 (d, J = 15.5 Hz, 2H), 5.14 (s, 1H), 3.94 – 3.77 (m, 2H), 3.76 – 3.63 (m, 8H), 3.05 – 2.95 (m, 2H), 1.49 (d, J = 6.6 Hz, 3H). ^{13}C NMR (101 MHz, DMSO) δ 164.45, 160.26, 157.94, 150.20, 148.84, 137.76, 134.78, 131.44, 127.41, 119.72, 115.22, 111.02, 80.45, 66.31, 63.63, 63.26, 47.21, 44.86, 43.08, 38.83, 37.65, 21.71. MS (ESI): *m/z* $[M+H]^+$ = 505.15. HRMS (ESI): *m/z* calcd for C23H26Cl2N6O3 $[M+H]^+$ 505.1516, found 505.1499.

To synthesize compounds **MRS-10** and **MRS-15**, compounds **11e** and **11i**, respectively, were dissolved in a mixture of MeOH, THF, and H_2O (1 mL:1 mL:1 mL) containing KOH (8 *eq*). The reaction mixtures were stirred at 80 °C for 4 h. After completion, the solvents were removed under reduced pressure to afford residues, which were diluted with water and acidified to pH = 4 using 1 N HCl. The resulting

precipitates were collected by filtration, washed several times with cold water, and dried under vacuum to yield **MRS-10** and **MRS-15**.

Compound **MRS-15**, white soild. ^{1}H NMR (400 MHz, DMSO-d_6) δ 9.33 (s, 1H), 9.23 (t, *J* = 5.6 Hz, 1H), 8.11 (s, 1H), 6.99 (d, *J* = 6.9 Hz, 1H), 6.59 (s, 1H), 6.46 (s, 2H), 5.73 (s, 1H), 5.21 – 5.05 (m, 1H), 3.91 (d, *J* = 5.6 Hz, 2H), 3.74 – 3.67 (m, 8H), 1.50 (d, *J* = 6.5 Hz, 3H). ^{13}C NMR (101 MHz, DMSO) δ 171.53, 164.16, 160.27, 157.94, 150.18, 148.90, 137.75, 134.82, 131.05, 127.23, 119.46, 115.24, 110.95, 80.49, 66.31, 47.36, 44.86, 41.82, 21.54. MS (ESI): *m/z* $[M+H]^+$ = 520.10. HRMS (ESI): *m/z* calcd for C23H23Cl2N5O5 $[M+H]^+$ 520.1149, found 520.1164.

Supplementary Figures

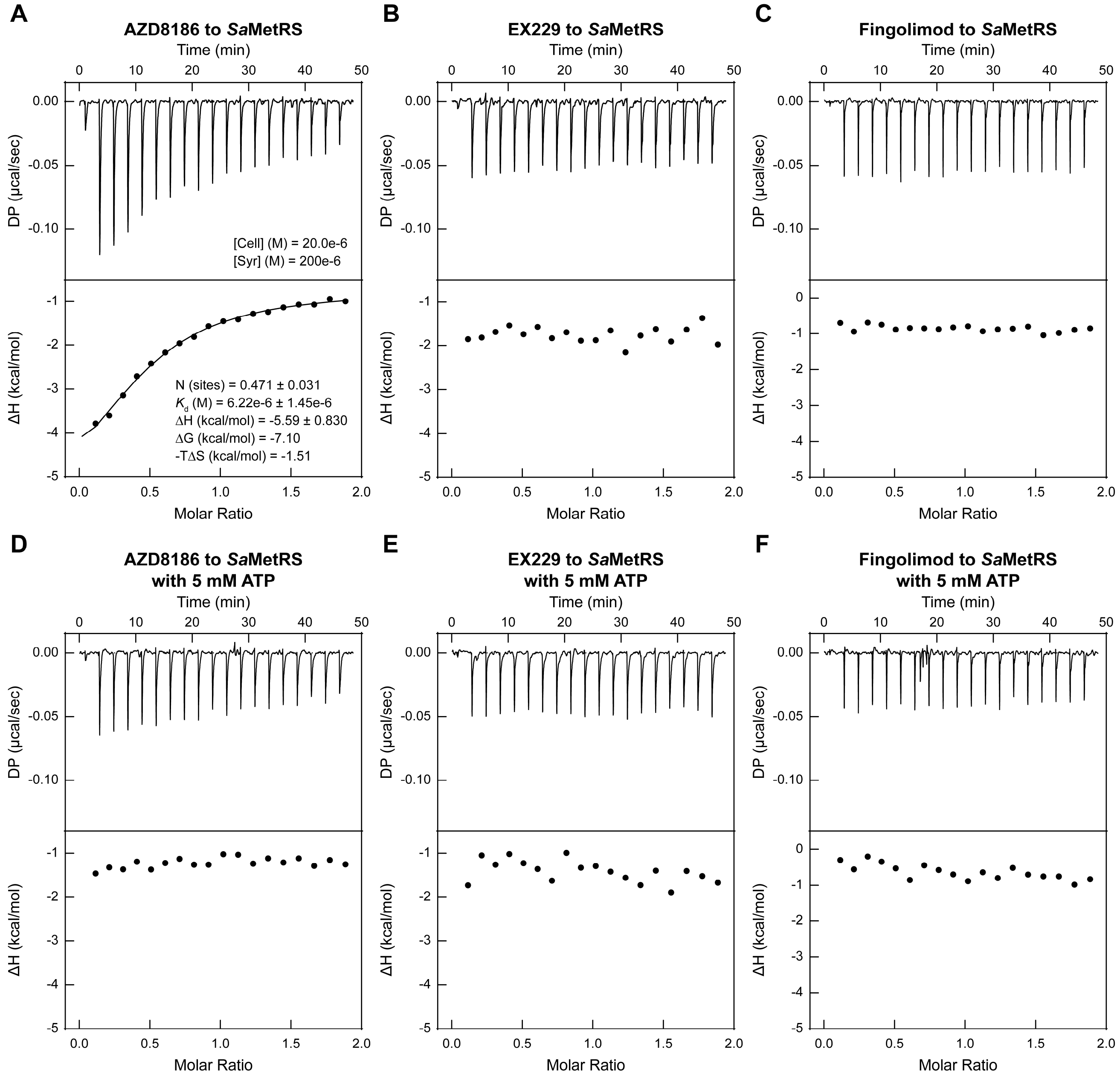

Supplementary Figure S1. Isothermal titration calorimetry (ITC) assays confirmed the binding of AZD8186 to *Sa*MetRS in the absence of a high concentration of ATP. (**A**-**C**) The ITC titrations of the compounds AZD8186, EX229, and Fingolimod with *Sa*MetRS. (**D**-**F**)The ITC titrations of the compounds AZD8186, EX229, and Fingolimod with *Sa*MetRS in the presence of 5 mM ATP.

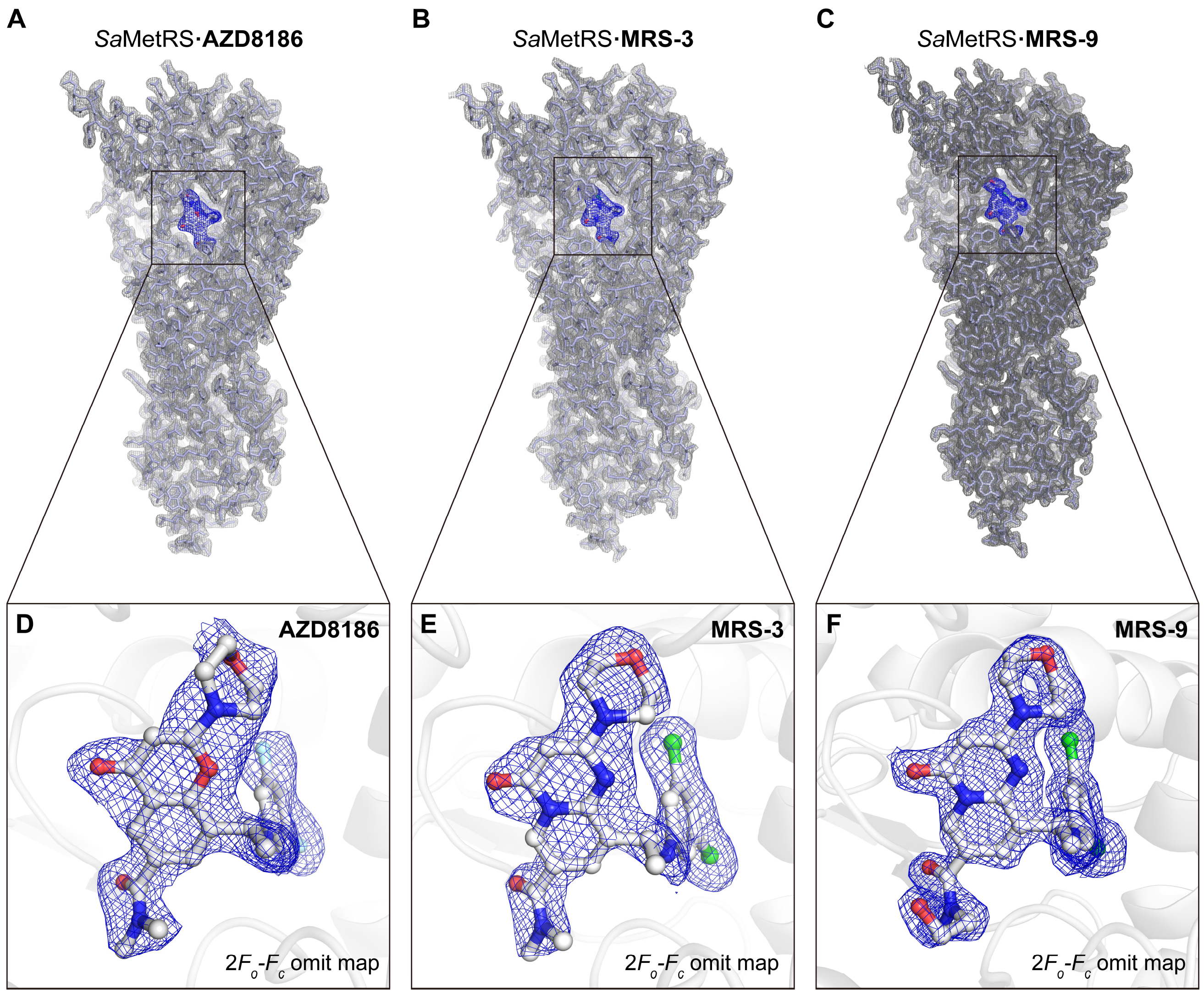

Supplementary Figure S2. Electron density maps of three co-crystal structures. (**A-C**) The overall $2F_o$-F_c electron density maps of the *Sa*MetRS·AZD8186, *Sa*MetRS·**MRS-3**, and *Sa*MetRS·**MRS-9** complexes, respectively. (**D-F**) The zoom-in view of the $2F_o$-F_c omit electron density maps for AZD8186, **MRS-3**, and **MRS-9**, shown as blue meshes and contoured at a level of 1.0 σ.

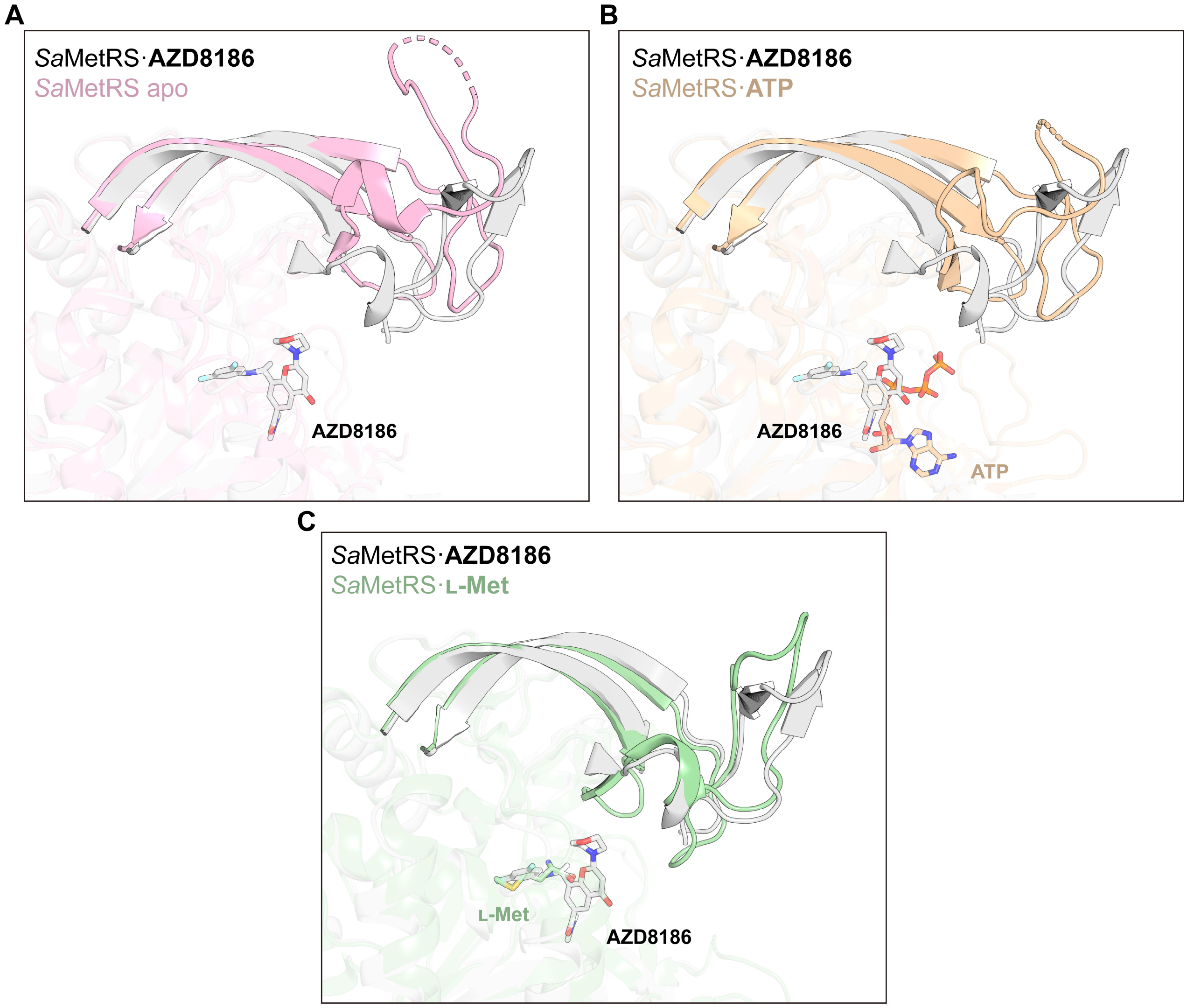

Supplementary Figure S3. Comparison of the knuckle region structure within the *Sa*MetRS·AZD8186 complex with that of *Sa*MetRS in its apo (**A**), ATP-bound (**B**), and L-Met-bound (**C**) states. Following the alignment of the CP domain of *Sa*MetRS across these different ligand-bound states, the conformation of the knuckle region of the CP domain in the *Sa*MetRS·AZD8186 complex closely resembles that observed in the L-Met bound state (colored in green, PDB code 7WPK). In contrast, it differs from the conformations observed in the apo (colored in pink, PDB code 7WPJ) and the ATP-bound (colored in yellow, PDB code 7WPL) states. For enhanced clarity, regions of the CP domain outside the knuckle region are rendered semi-transparent.

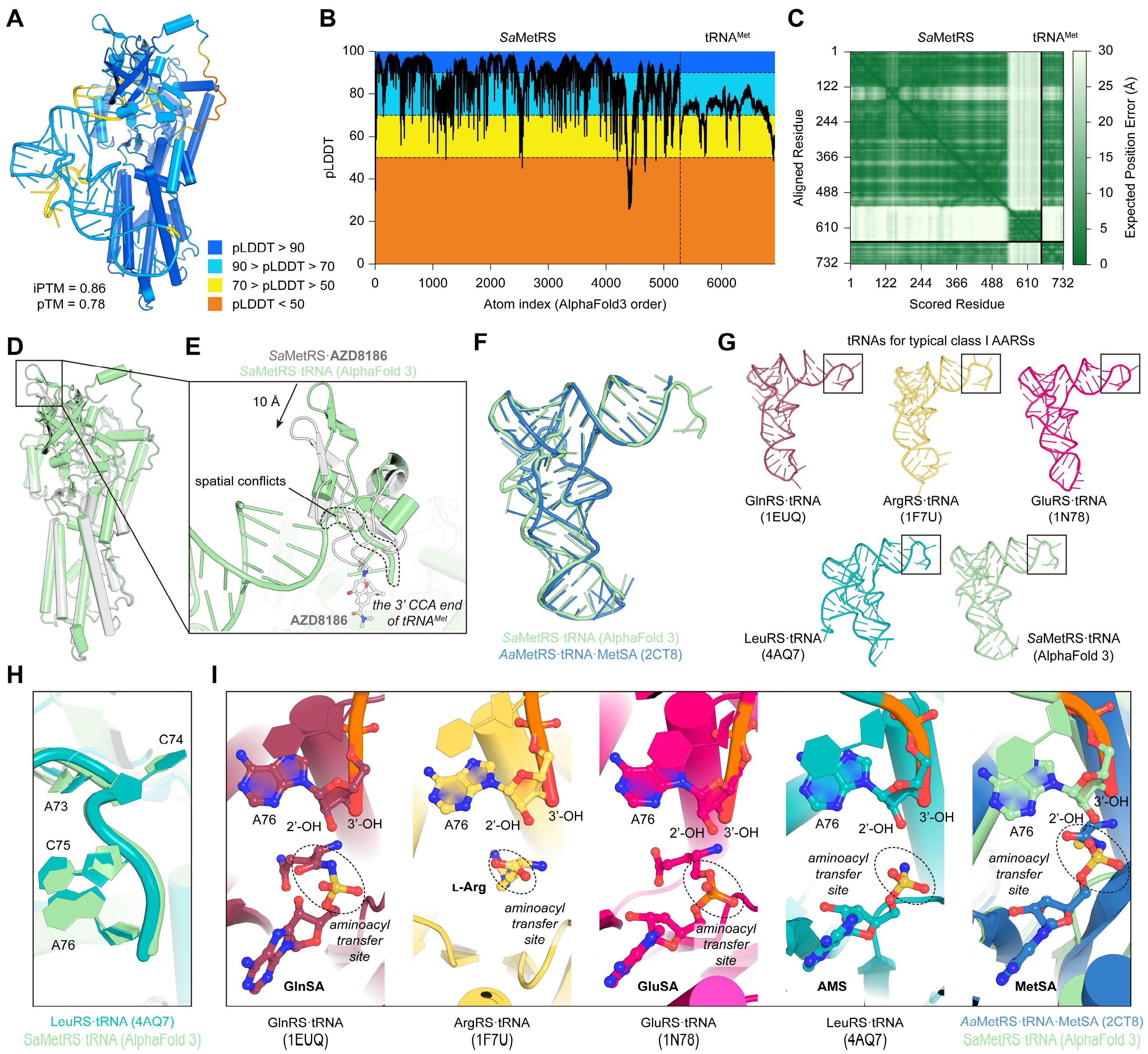

Supplementary Figure S4. The predicted structural model of the *Sa*MetRS·tRNA^{Met} complex generated using AlphaFold 3, along with analyses conducted to assess its reliability. (**A**) Overall 3D structure of the predicted *Sa*MetRS·tRNA^{Met} complex, color-coded by pLDDT scores. (**B**) The pLDDT scores of the predicted model at atomic resolution. (**C**) The predicted aligned error (PAE) plot of the model. (**D**) Superposition of the MetRS structure from the predicted *Sa*MetRS·tRNA^{Met} complex model with that in the co-crystal structure of the *Sa*MetRS·AZD8186 complex demonstrates close agreement except for the knuckle region of the CP domain. (**E**) In the predicted *Sa*MetRS·tRNA^{Met} complex model, the knuckle region is displaced upward by approximately 10 Å, a shift that may facilitate accommodation of the 3' CCA end of tRNA^{Met} with the active-site cavity for aminoacylation. (**F**) Superposition of tRNA^{Met} from the predicted *Sa*MetRS·tRNA^{Met} complex model with that from the co-crystal structure of the *Aa*MetRS·tRNA^{Met} complex (PDB code 2CT8) demonstrates overall agreement except for disorder observed in the 3' CCA end of RNA^{Met} in the co-crystal structure. (**G**) The 3' CCA end of tRNAs bound to

class I aaRSs typically adopts a hairpin conformation. The 3’ CCA end in the predicted *Sa*MetRS·tRNA^{Met} complex model is consistent with this feature. (**H**) Superposition of the 3’ CCA end in the predicted *Sa*MetRS·tRNA^{Met} complex model with that in the co-crystal structure of the LeuRS·tRNA complex in aminoacylation state reveals close overlap. (**I**) The 2’-OH of nucleotide A76 in the predicted *Sa*MetRS·tRNA^{Met} complex model is positioned adjacent to the intermediate analog MetSA, which is consistent with that class I aaRSs acylate the 2’-OH rather than the 3’-OH of A76.

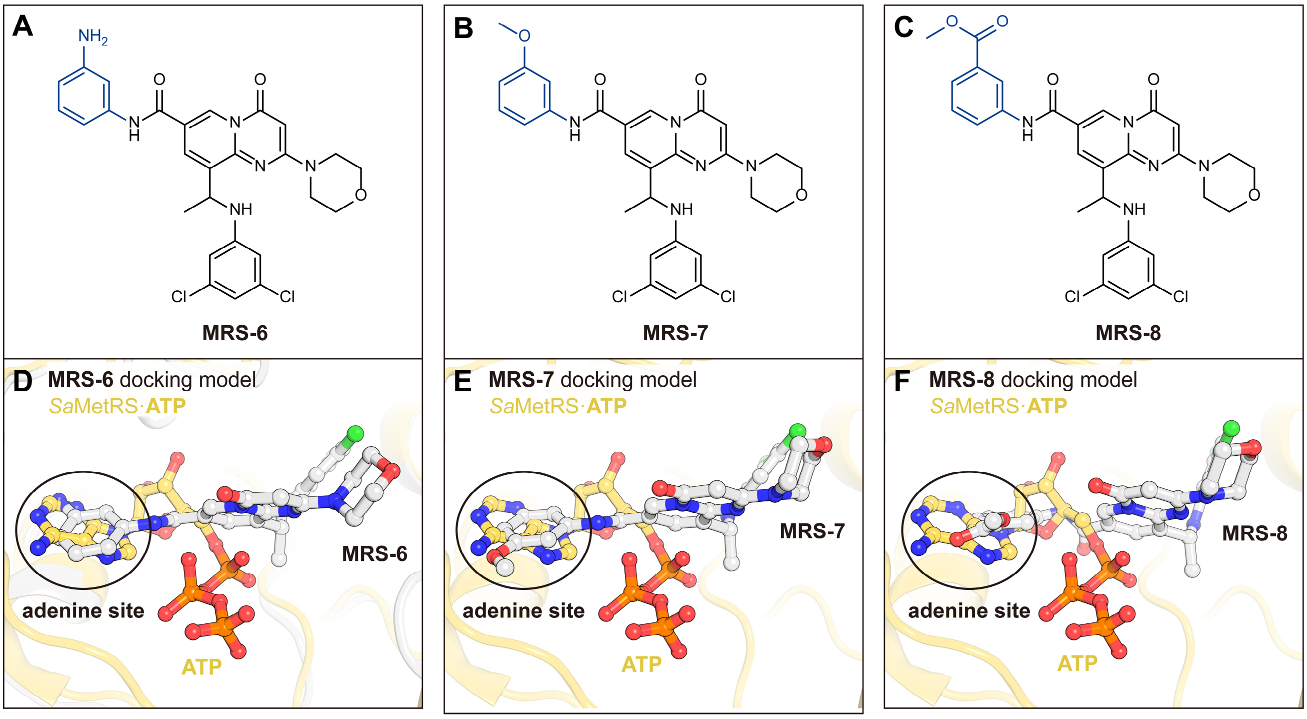

Supplementary Figure S5. Chemical structures and molecular docking models of **MRS-6** to **MRS-8**. (**A**) Chemical structure of **MRS-6**. (**B**) Chemical structure of **MRS-7**. (**C**) Chemical structure of **MRS-8**. (**D-F**) Docking of the three compounds into the active site cavity of *Sa*MetRS. Structural superimposition of these docking models with the ATP-bound *Sa*MetRS (PDB code 7WPL) supports that **MRS-6** to **MRS-8** bind within the active site cavity of *Sa*MetRS, with their substituted aromatic groups occupying the adenine binding site.

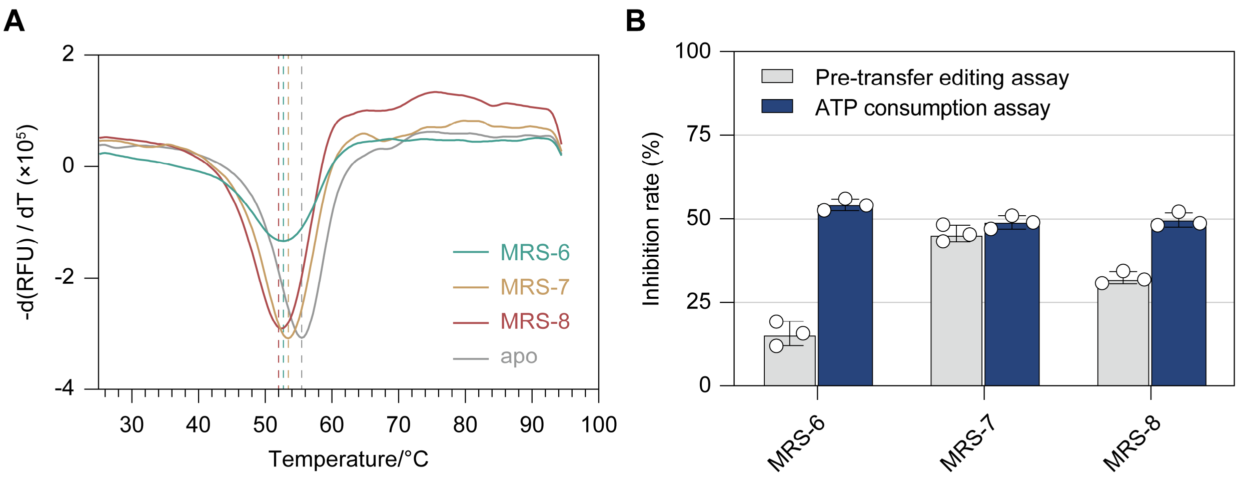

Supplementary Figure S6. TSA and enzymatic activity data for compounds **MRS-6** to **MRS-8**. (**A**) The TSA assay revealed no increase, but rather a slight decrease, in the T_m value of *Sa*MetRS upon the addition of **MRS-6**, **MRS-7**, and **MRS-8**, relatived to the apo enzyme. (**B**) The pre-transfer editing assay and ATP consumption assay showed that compounds **MRS-6**, **MRS-7**, and **MRS-8** inhibited *Sa*MetRS activity by approximately 50% or less, even at a concentration of 100 μM. In comparison to **MRS-3**, these compounds exhibited a substantial loss of inhibitory activity against *Sa*MetRS. The results are presented as mean ± SD (n = 3).

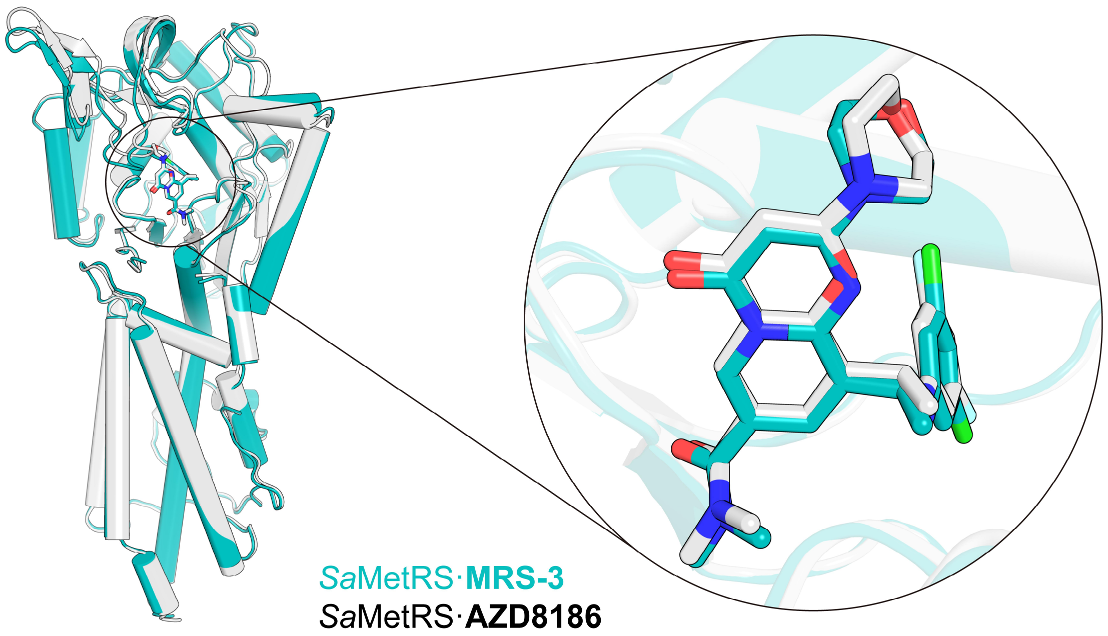

Supplementary Figure S7. Structural comparison between the *Sa*MetRS·**MRS-3** and the *Sa*MetRS·AZD8186 complexes. The *Sa*MetRS·**MRS-3** complex demonstrated a binding mode almost identical to that of the *Sa*MetRS·AZD8186 complex, indicating that scaffold hopping from benzopyranone to pyrido[1,2-a]pyrimidine preserves compatibility with the active site cavity of *Sa*MetRS.

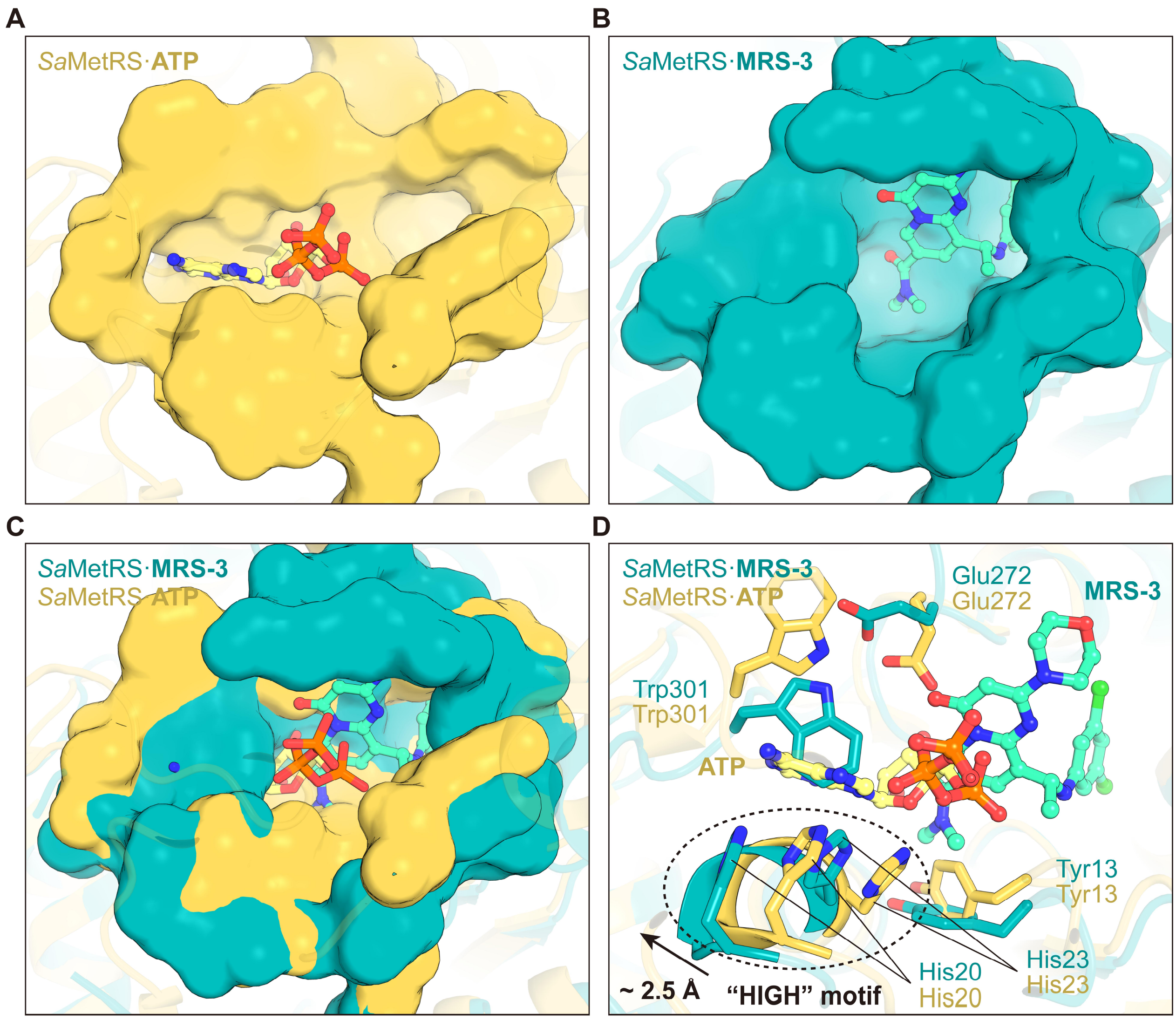

Supplementary Figure S8. Structural comparison of the *Sa*MetRS·**MRS-3** complex with the *Tb*MetRS·MetSA complex (PDB code 4EG3). (**A**) Surface representation of the active site cavity of *Tb*MetRS in complex with MetSA. (**B**) Surface representation of the active site cavity of *Sa*MetRS in complex with **MRS-3**. (**C**) Superimposed surface representations of the *Tb*MetRS·MetSA and *Sa*MetRS·**MRS-3** complexes. (**D**) Comparison of ATP binding site residues between MetRS in the MetSA-bound and **MRS-3**-bound states. The result reveals significant conformational differences in the ATP binding site residues between the **MRS-3**-bound and MetSA-bound states.

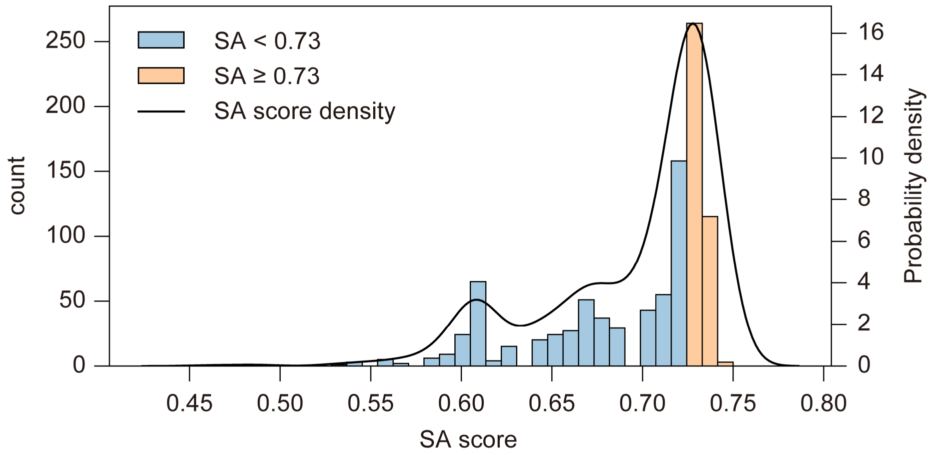

Supplementary Figure S9. The distribution of synthetic accessibility (SA) score for the 1,000 molecules generated by DiffDeCIG. The SA score for each compound was calculated using RDKit. Molecules that could not be parsed by RDKit or contained invalid structures were excluded from the analysis.

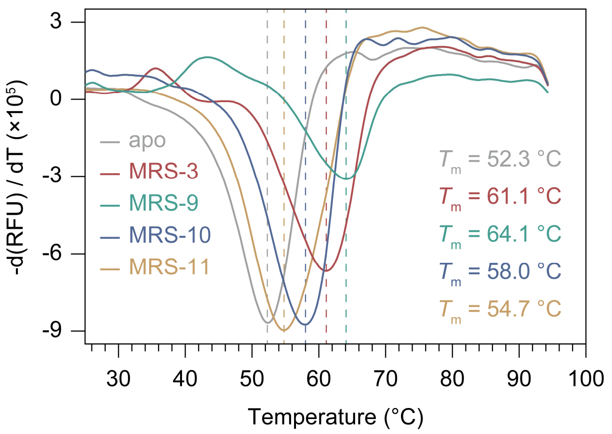

Supplementary Figure S10. TSA curves of *Sa*MetRS in the presence of selected molecules. Each curve represents the average of three measurements.

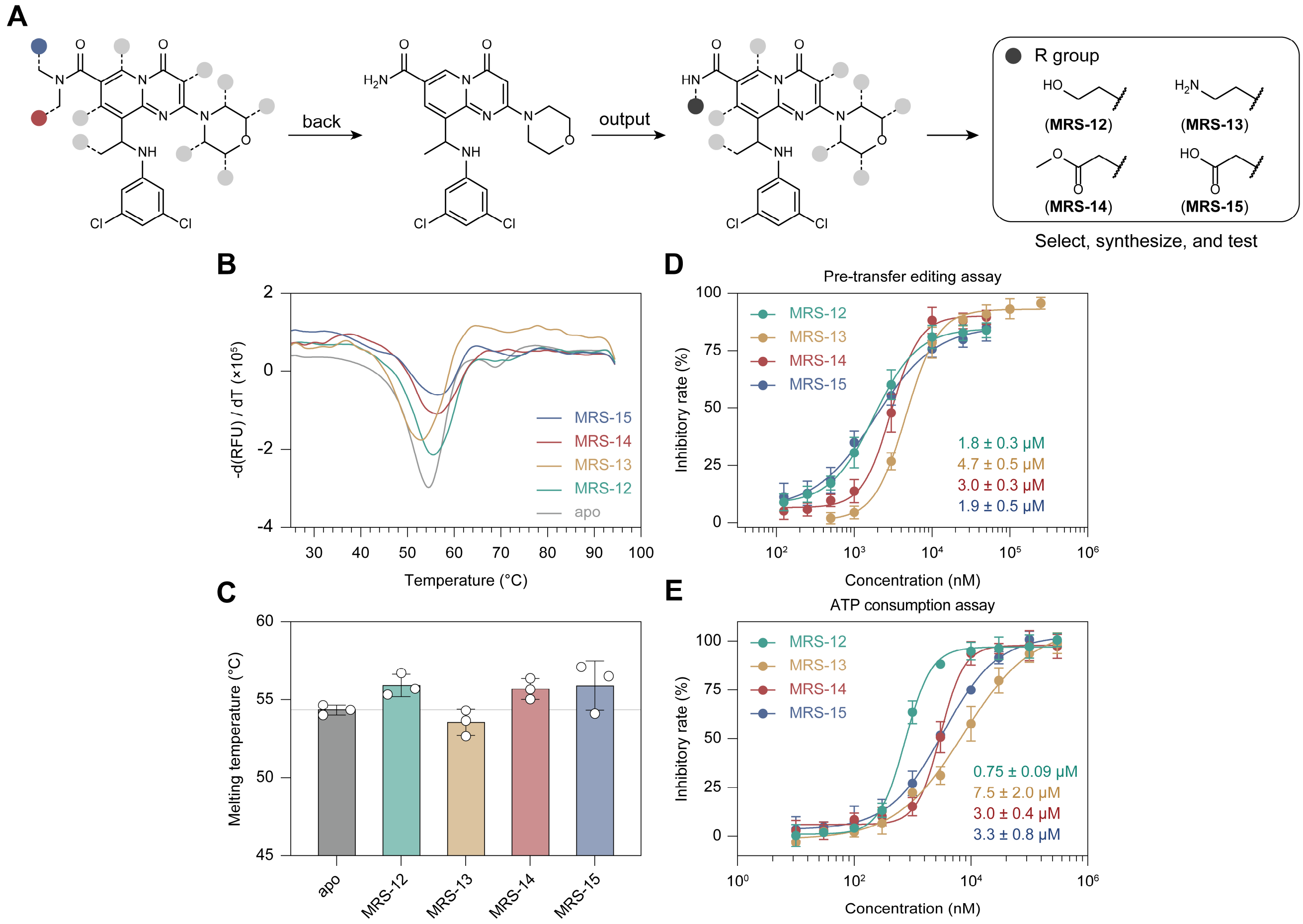

Supplementary Figure S11. The design and evaluation of compounds **MRS-12** to **MRS-15**. (**A**) A schematic illustration depicting the molecular generation process using DiffDeCIG with a rollback of decoration site from the *N*,*N*-dimethyl group to the amide group, alongside the chemical structures of the selected compounds. (**B**) Representative melting curves of *Sa*MetRS in the presence or absence of compounds **MRS-12** to **MRS-15**. (**C**) The T_m values of *Sa*MetRS in the presence or absence of compounds **MRS-12** to **MRS-15**, derived from the melting curves. The results are presented as mean ± SD (n = 3). (**D**) IC_{50} curves of compounds **MRS-12** to **MRS-15**, as determined by a pre-transfer editing assay. The results are presented as mean ± SD (*n* = 3). (**E**) IC_{50} curves of compounds **MRS-12** to **MRS-15**, as determined by an ATP consumption assay. The results are presented as mean ± SD (*n* = 3).

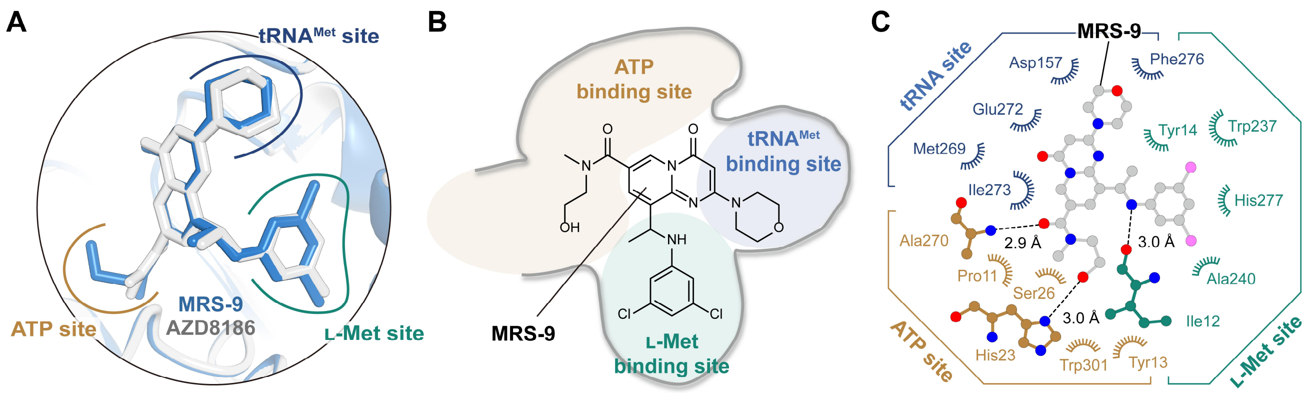

Supplementary Figure S12. The binding mode of **MRS-9** with *Sa*MetRS. (**A**) Comparison of the binding modes of **MRS-9** and AZD8186 within the active site cavity of *Sa*MetRS. (**B**) A schematic representation of the triple-site binding mode of **MRS-9** within the active site cavity of *Sa*MetRS. (**C**) A 2D representation detailing the interactions between the compound **MRS-9** and the residues within the active site cavity of *Sa*MetRS.

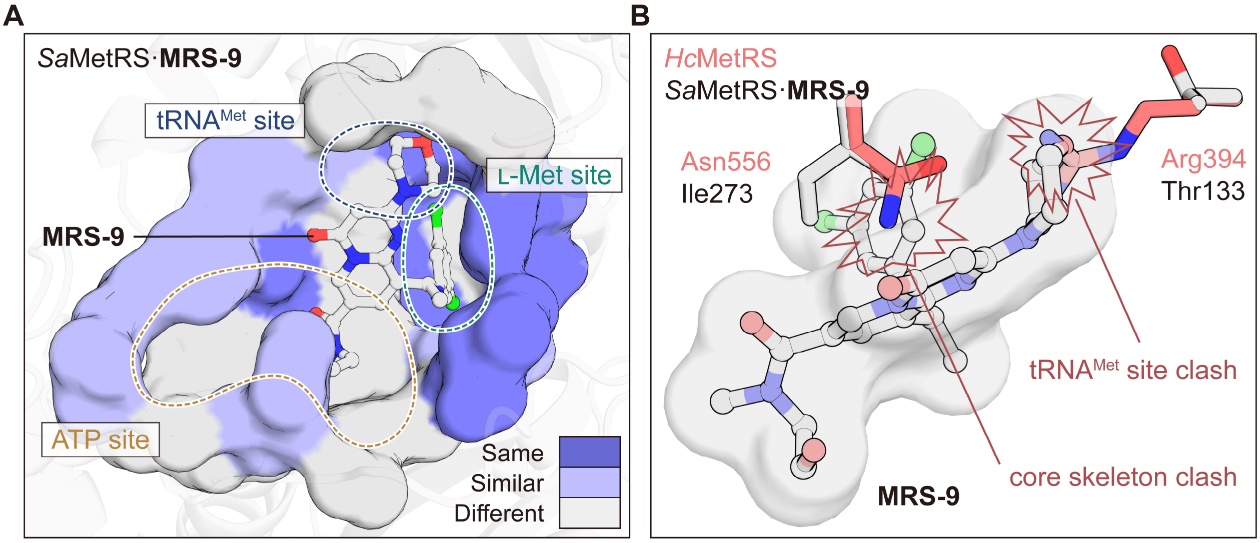

Supplementary Figure S13. Structural basis underlying the species selectivity of **MRS-9**. (**A**) Residues of *Sa*MetRS surrounding the **MRS-9** binding site are colored according to their similarity to their corresponding residues in *Hc*MetRS. (**B**) Potential conflicts of *Hc*MetRS active sites residues with **MRS-9**. The structural model of *Hc*MetRS was built using homology modeling program SWISS-MODEL[14] based on the co-crystal structure of the *Sa*MetRS·**MRS-9** complex, and subsequently superimposed onto the *Sa*MetRS·**MRS-9** structure. Severe clashes were observed between **MRS-9** and the residues Asn556 and Arg394 of *Hc*MetRS, indicating that the unique triple-site binding mode of **MRS-9** is likely accommodated exclusively by type 1 MetRSs, but not by type 2 MetRSs such as *Hc*MetRS.

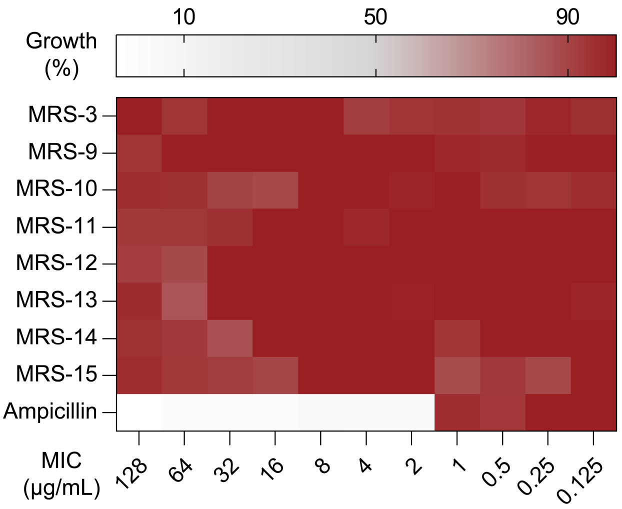

Supplementary Figure S14. MIC values of **MRS-3** and its derivatives **MRS-9** to **MRS-15** against *E.coli* strain ATCC25922. The results are presented as mean ± SD (*n* = 3).

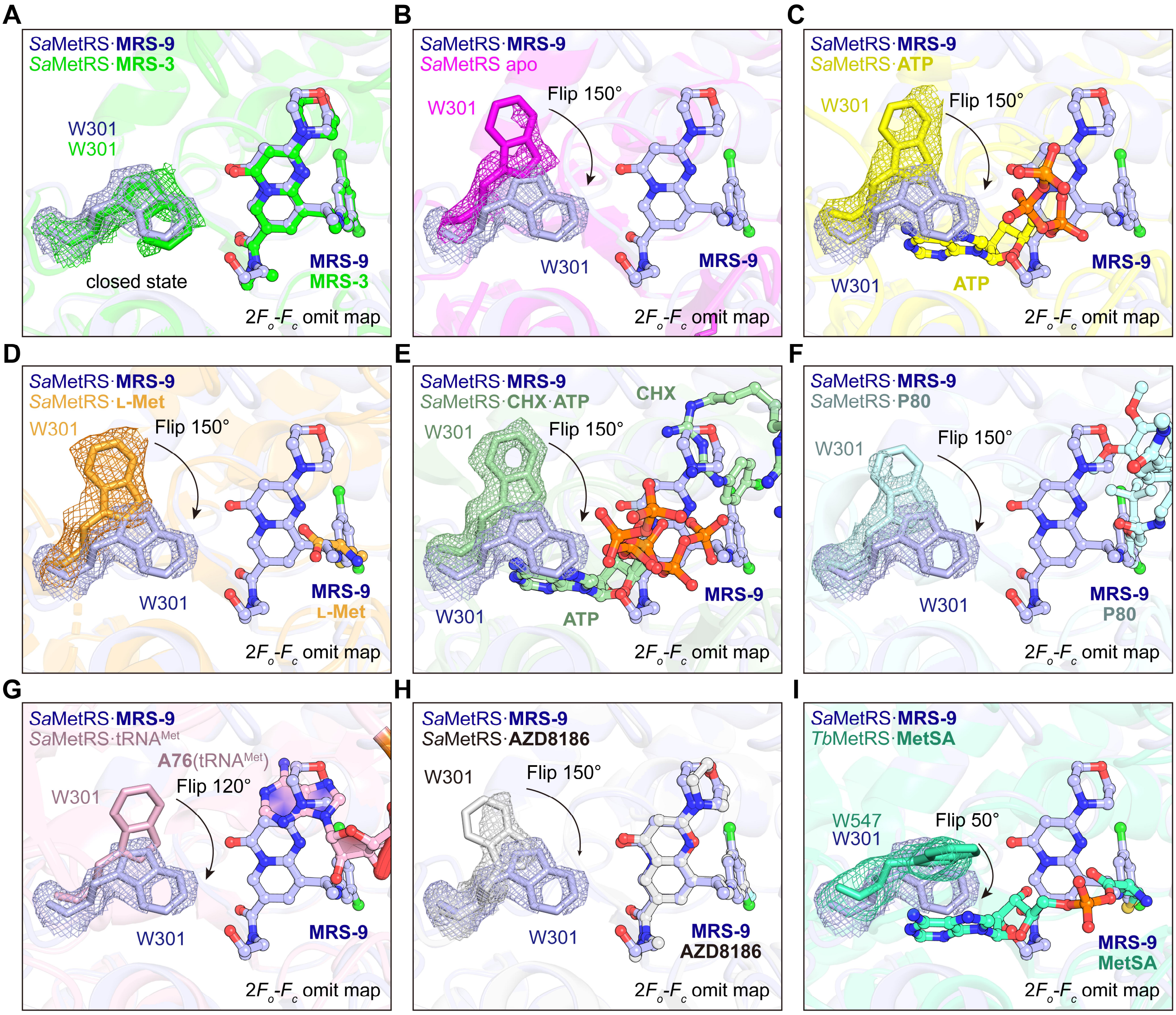

Supplementary Figure S15. Conformation changes of the residue Trp301 in *Sa*MetRS across various ligand-bound states. (**A**) In the structures of the *Sa*MetRS·**MRS-9** and *Sa*MetRS·**MRS-3** complexes, the side chain of Trp301 undergoes a flip, resulting in the closure of the binding pocket for the adenine group of ATP. This unique conformation of Trp301, together with the closed state of the adenine pocket in the *Sa*MetRS·**MRS-9** and *Sa*MetRS·**MRS-3** complexes, differs markedly from those observed in other *Sa*MetRS states, including the apo state (PDB code 7WPJ) (**B**), the ATP-bound state (PDB code 7WPL) (**C**), the L-Met-bound state (PDB code 7WPK) (**D**), the amino acid-auxiliary dual-site inhibitor CHX-bound state (PDB code 8XM4) (**E**), the inhibitor P80-bound state (PDB code 7WPI) (**F**), the tRNA-bound state (AlphaFold 3 model) (**G**), and even the initial triple-site inhibitor AZD8186-bound state (**H**), as well as those observed in the *Tb*MeRS structure at the amino acid-ATP dual-site inhibitor MetSA-bound state (PDB code 4EG3) (**I**). The $2F_o$-F_c omit electron density maps surrounding the Trp301 are presented as meshes contoured at 1.0 σ.

Supplementary Tables

Supplementary Table S1. Statistics of X-ray diffraction data collection and structure refinement.

	*Sa*MetRS·**AZD8186**	*Sa*MetRS·**MRS-3**	*Sa*MetRS·**MRS-9**
**PDB accession code**	9V9M	9V9D	9V9F
**Data collection**			
Wavelength (Å)	1.5418	1.5418	0.9792
Resolution (Å)	23.64-2.50 (2.59-2.50) [a]	26.98-2.50 (2.59-2.50)	46.96-2.00 (2.05-2.00)
Space group	$P2_12_12_1$	$P2_12_12_1$	$P2_12_12_1$
Cell dimensions			
a, b, c (Å)	59.6, 77.2, 119.5	54.0, 74.1, 116.7	59.2, 76.8, 118.8
α, β, γ (°)	90.0, 90.0, 90.0	90.0, 90.0, 90.0	90.0, 90.0, 90.0
Unique reflections	19687 (1975)	16849 (1691)	37385 (2747)
Redundancy	4.3 (2.8)	9.0 (9.3)	12.4 (12.7)
R_{merge} [b]	0.063 (0.218)	0.160 (0.390)	0.088 (0.256)
Average I/σ(I)	24.32 (4.66)	12.3 (5.1)	22.4 (9.9)
Completeness (%)	99.7 (99.9)	99.9 (100.0)	100.0 (100.0)
**Refinement**			
Resolution (Å)	23.63-2.50	26.98-2.50	46.96-2.00
Reflections for refinement/test	18617 (1010)	15959 (828)	35474 (1844)
R_{work} [c]/R_{free} (%) [d]	22.3 (25.6)	23.0 (25.7)	19.6 (21.9)
RMSD bond (Å)	0.002	0.002	0.003
RMSD angle (°)	1.13	1.18	1.14
Mean B factor ($Å^2$)	17.295	16.5	17.930
Non-hydrogen protein atoms	4032	4054	4021
Water oxygen atoms	213	167	531
Ligand/ion atoms	65	33	51
Ramachandran plot (%)			
Favored	96.0	97.4	98.0
Allowed	4.0	2.6	2.0
Outliers	0	0	0

[a] Values in parentheses are for the highest resolution shell.

[b] $R_{merge} = \sum_h \sum_l | I(h)_l - \langle I(h) \rangle | / \sum_h \sum_l I(h)_l$, where $I(h)_l$ is the *l*th observation of the reflection h and $\langle I(h) \rangle$ is the weighted average intensity for all observations *l* of reflection h.

[c] Rwork = $\sum_h ||F_{obs}(h)| - |F_{cal}(h)|| / \sum_h |F_{obs}(h)|$, where $F_{obs}(h)$ and $F_{cal}(h)$ are the observed and calculated structure factors for reflection h respectively.

[d] R_{free} was calculated as R_{work} using 5% of the reflections which were selected randomly and omitted from refinement.

Supplementary Table S2. The chemical structures and kinase targets of the hit compounds identified through experimental screening, and the TSA results of these compounds against *Sa*MetRS.

	Structure	ΔT_m	Target
**KHS101**	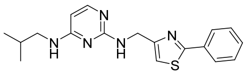	-4.0	FGFR; Microtubule Associated; TACC
**Fingolimod**	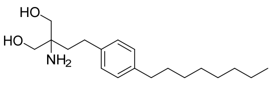	-3.2	LPL Receptor; PAK; S1P Receptor; TRP/TRPV Channel
**Honokiol**	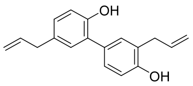	-11.2	Akt; Autophagy; ERK; HCV Protease; MEK
**CRT0066101**	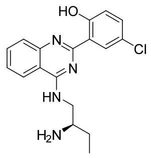	-2.2	Serine/threonin kinase
**CASIN**	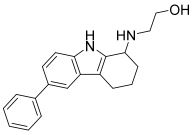	-3.2	CDK; Ras; STAT
**AZD8186**	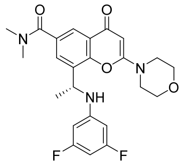	2.2	PI3K
**GMB475**	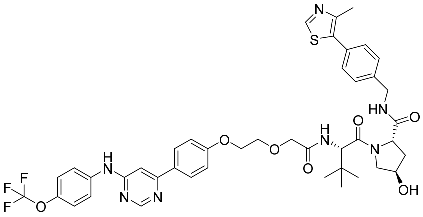	-2.2	Bcr-Abl
**EX229**	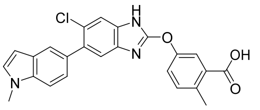	-8.3	AMPK

Supplementary Table S3. Comparison of molecule generative abilities of the two models.

	Validity [a]	Uniqueness [b]	Vina score [c]	High affinity [d]
**DiffDec**	**91.8**	53.3	-8.11	42.9%
**DiffDeCIG**	90.1	**67.3**	**-8.19**	**44.6%**

The better-performing methods are emphasized in bold.

[a] **Validity** refers to the percentage of generated molecules that both retain the original scaffolds and can be successfully parsed by RDKit.

[b] **Uniqueness** represents the proportion of unique compounds among all generated molecules.

[c] **Vina score** is used to estimate the binding affinity between the generated molecules and the target protein. This score is obtained through molecular docking using QVina and is expressed in units of kcal/mol.

[d] **High affinity** denotes the proportion of test cases in which the Vina scores of the generated molecules are equal to or better than that of the reference compound.

Supplementary NMR Spectra and Analytical Data

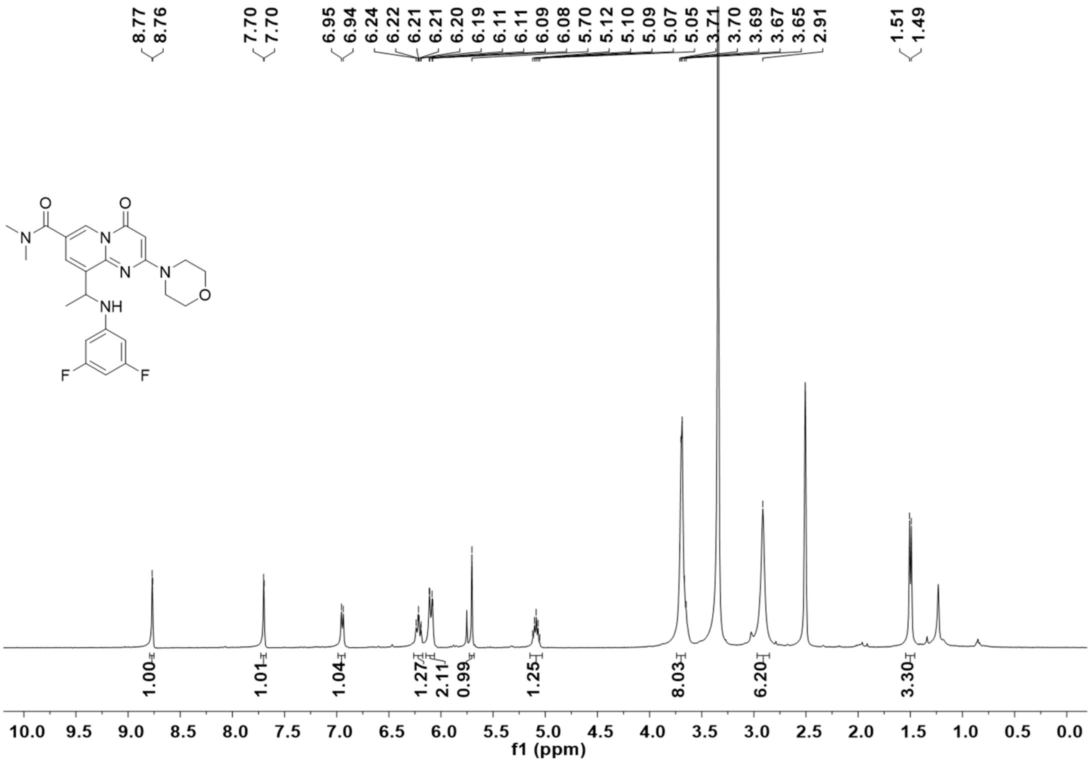

^{1}H NMR spectra of **MRS-1**

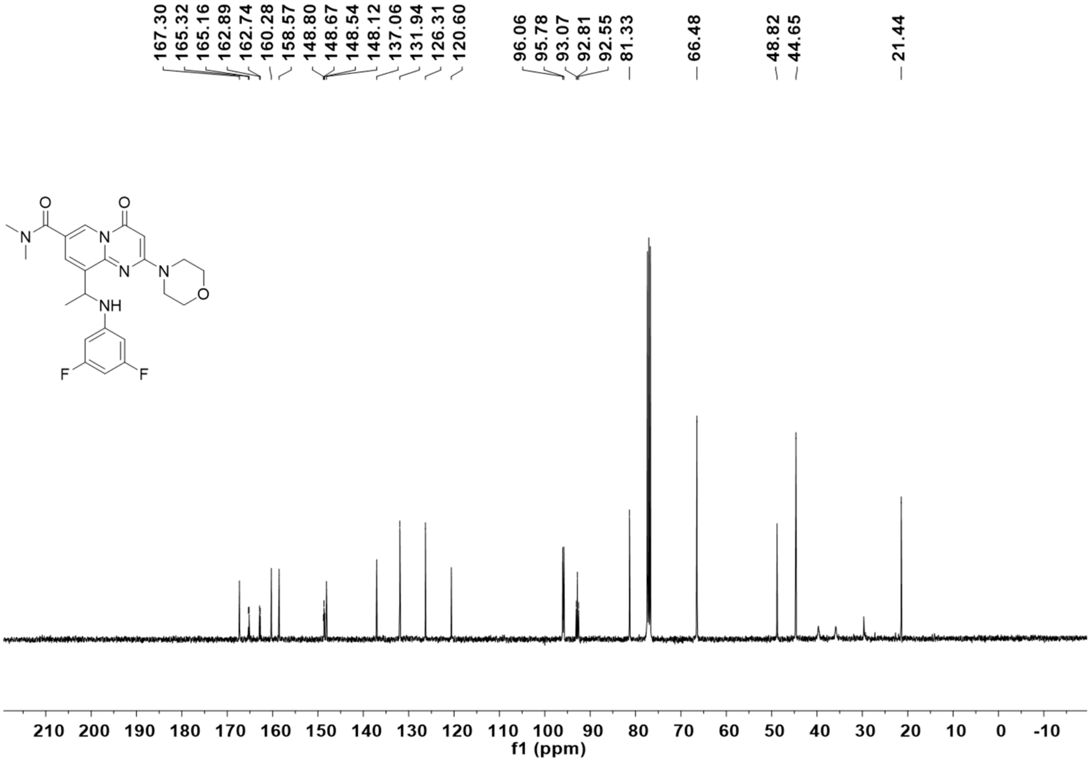

^{13}C NMR spectra of **MRS-1**

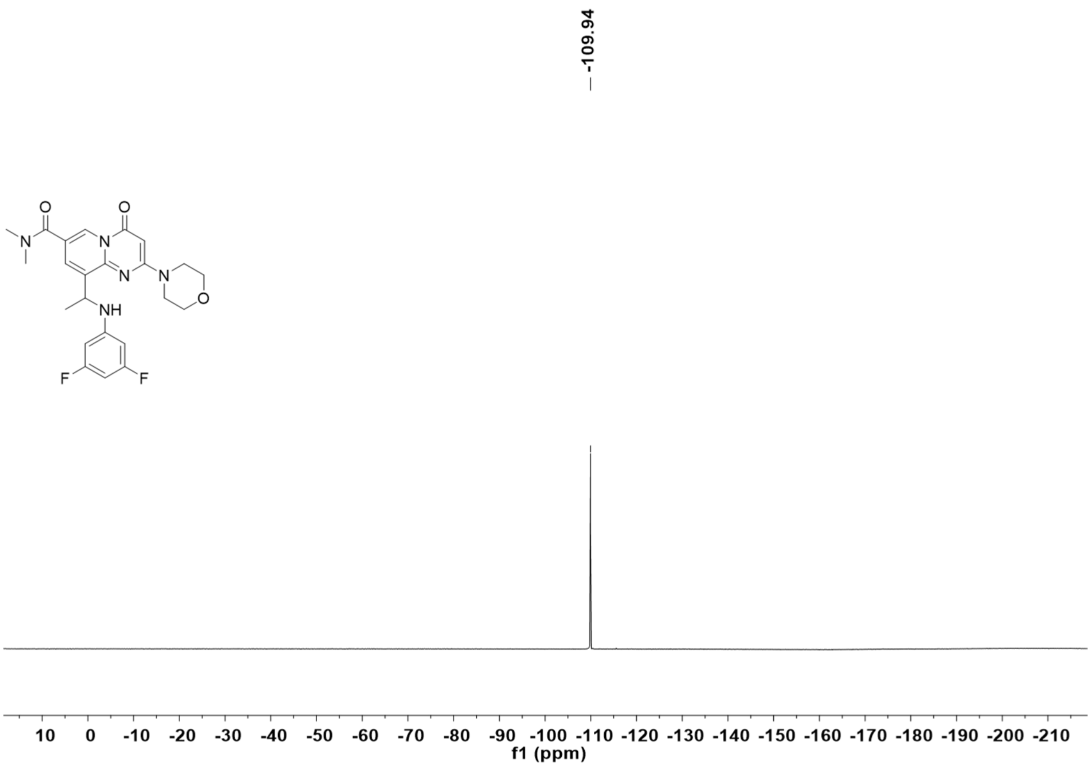

^{19}F NMR spectra of **MRS-1**

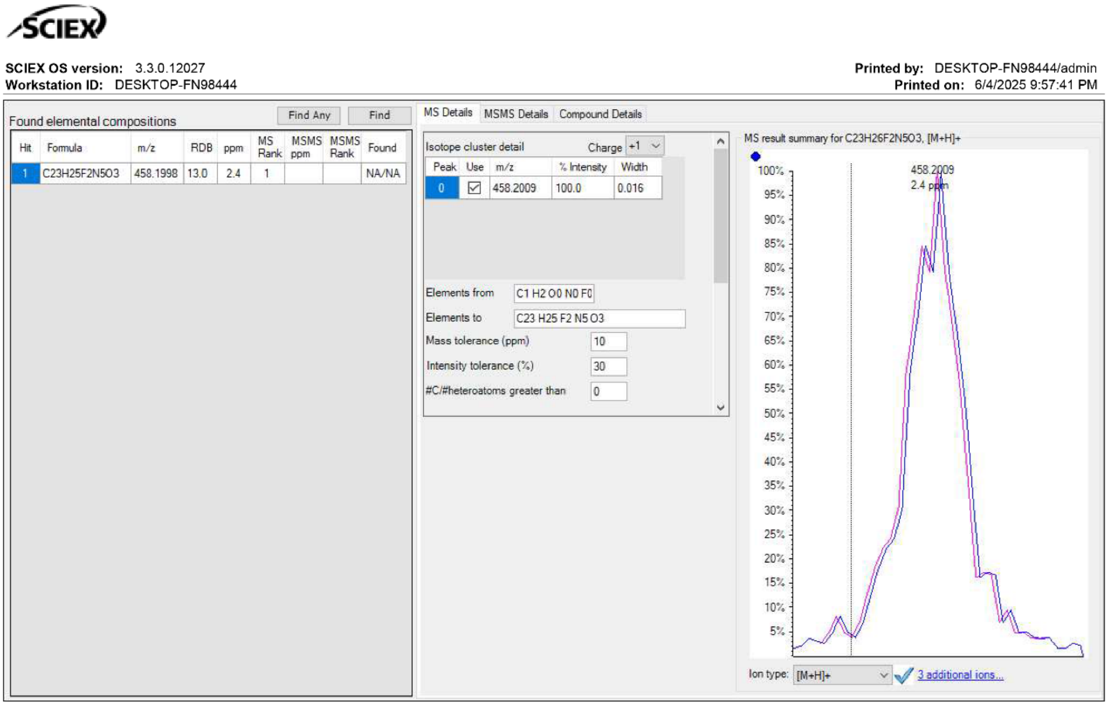

HRMS data of **MRS-1**

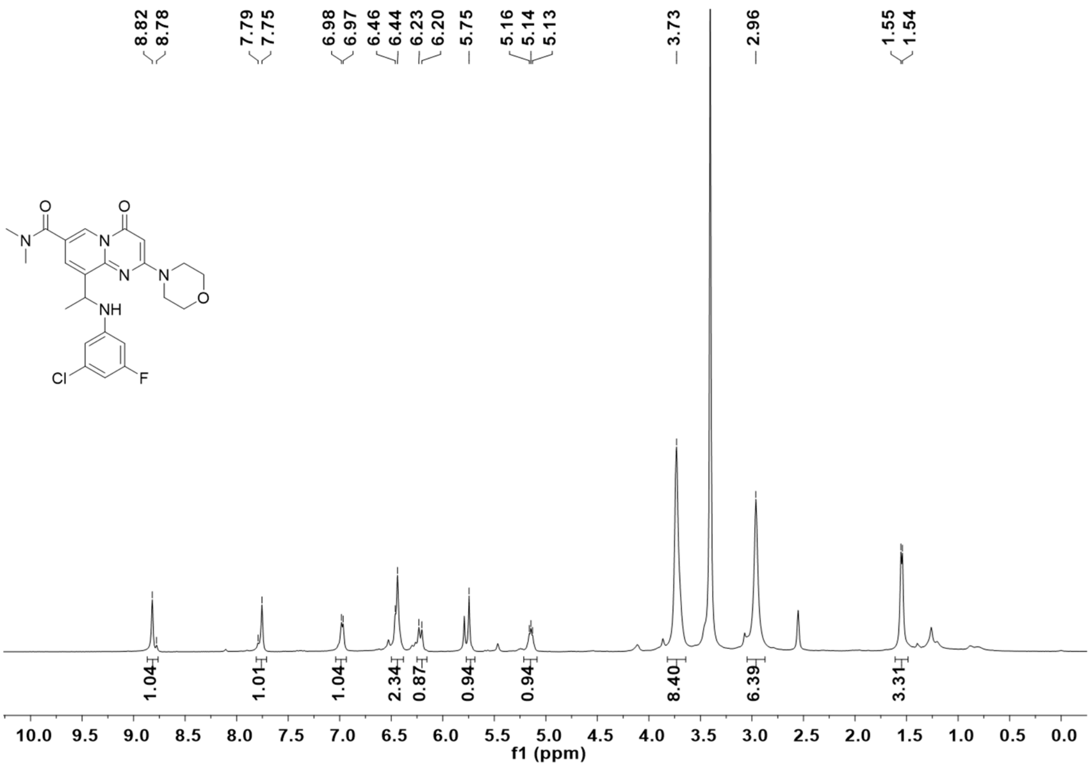

^{1}H NMR spectra of **MRS-2**

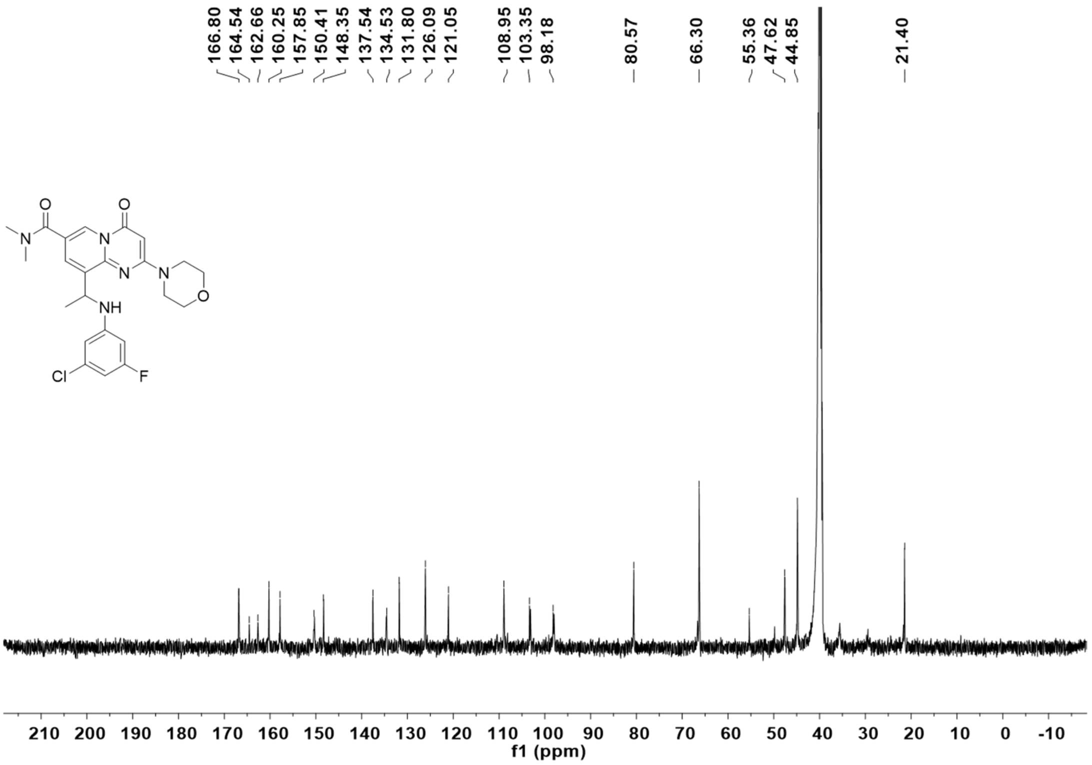

^{13}C NMR spectra of **MRS-2**

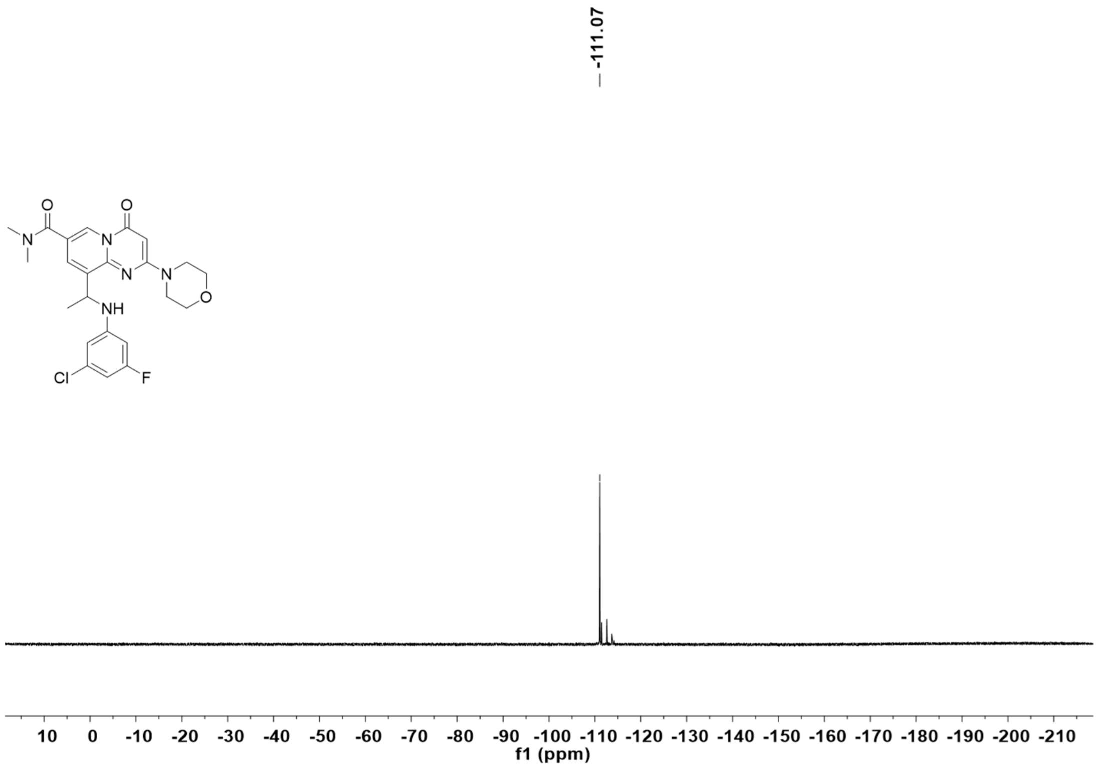

^{19}F NMR spectra of **MRS-2**

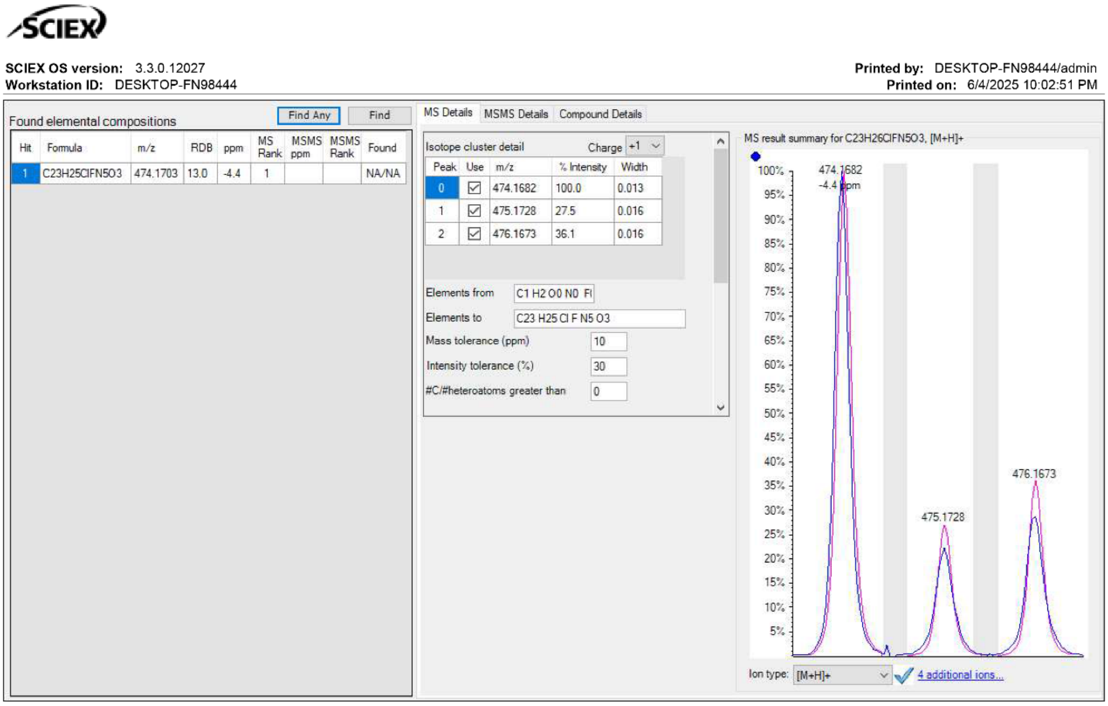

HRMS data of **MRS-2**

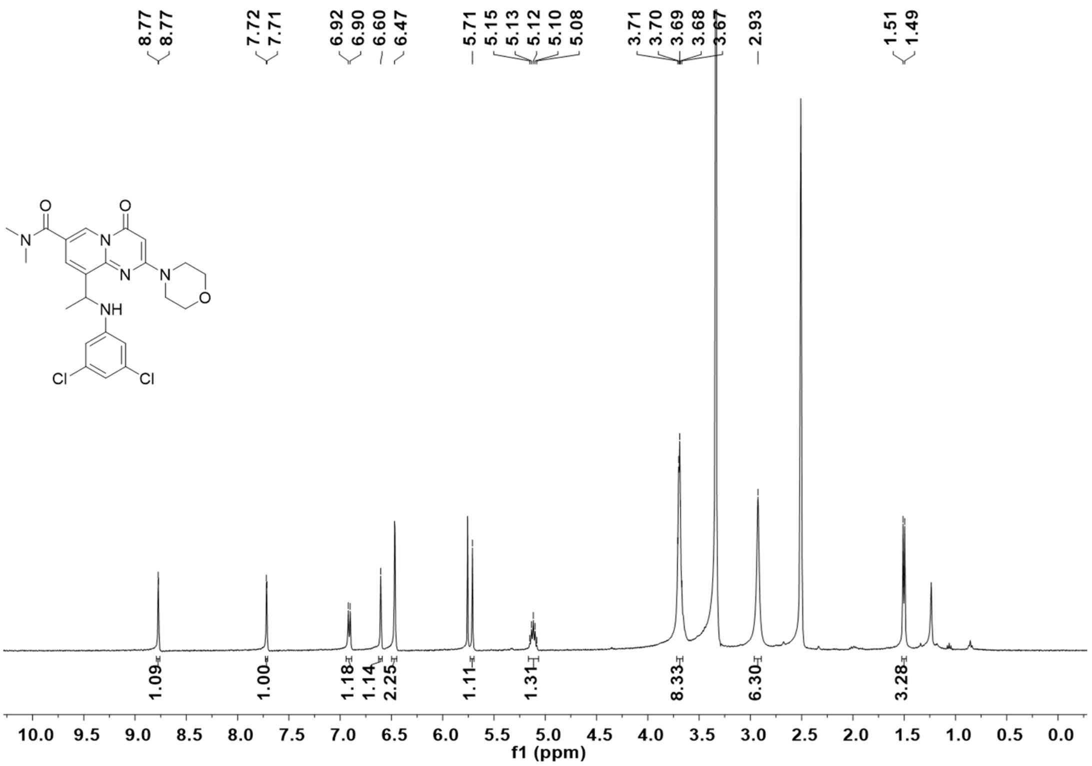

^{1}H NMR spectra of **MRS-3**

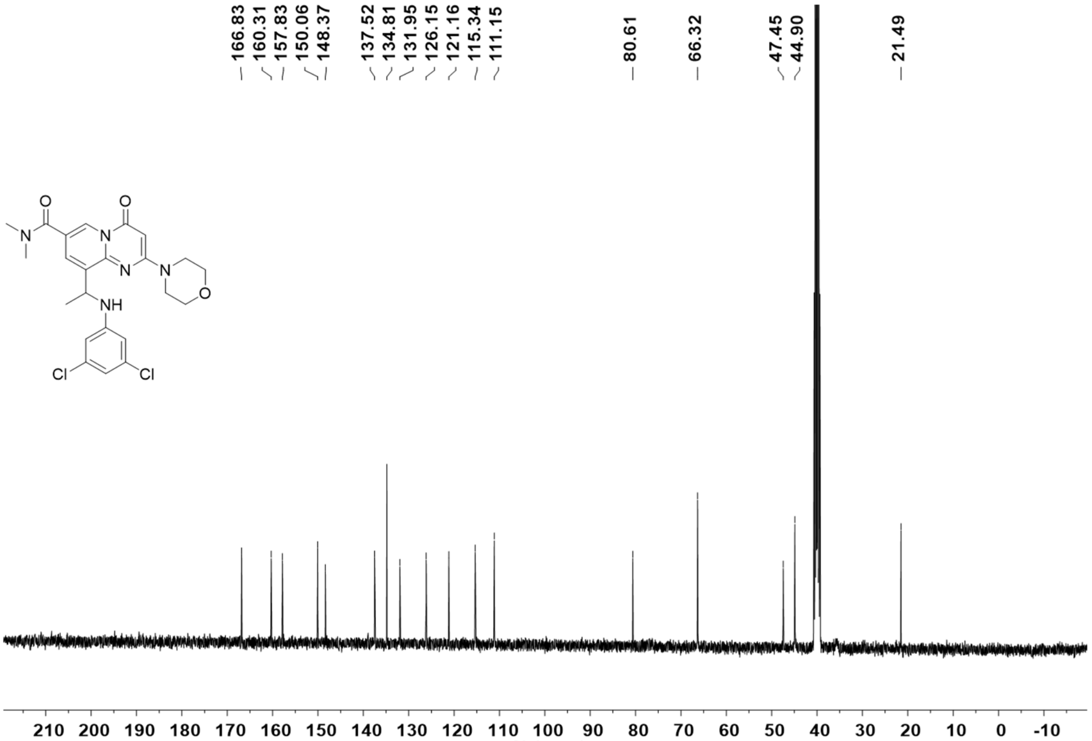

^{13}C NMR spectra of **MRS-3**

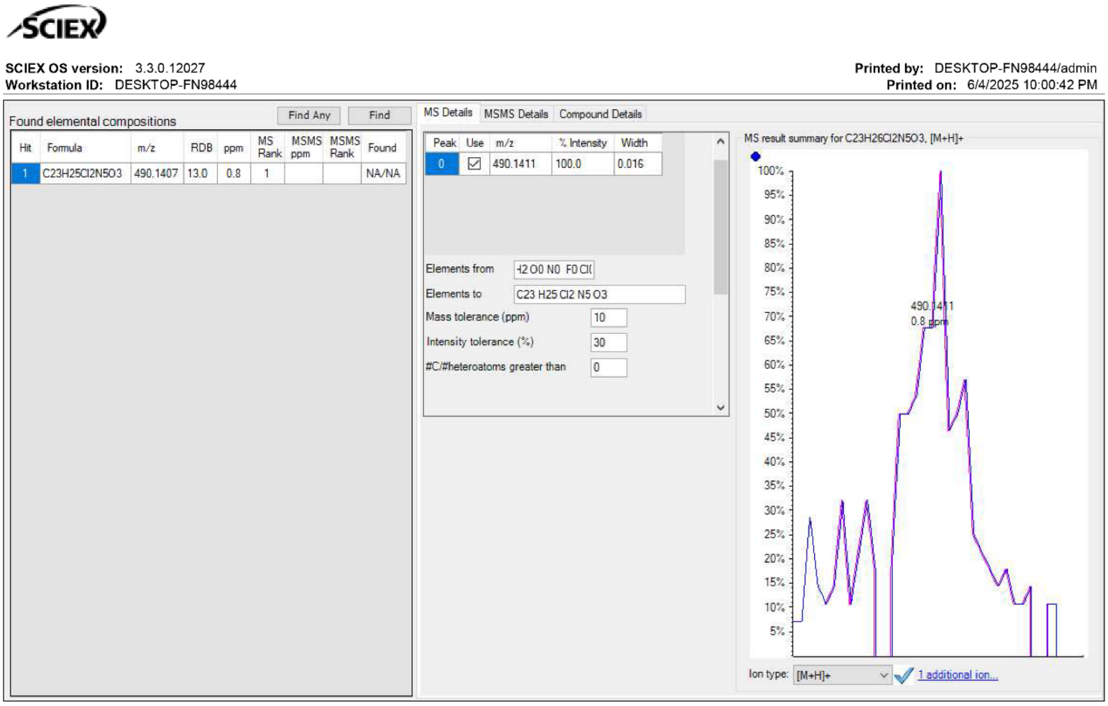

HRMS data of **MRS-3**

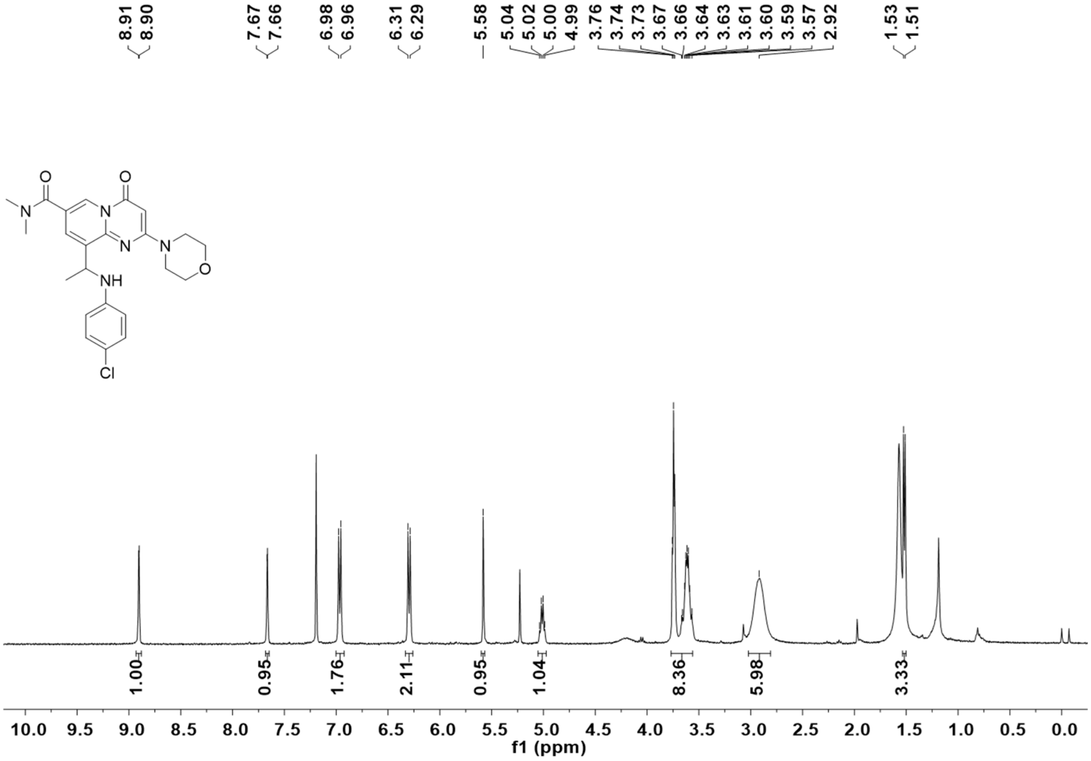

^{1}H NMR spectra of **MRS-4**

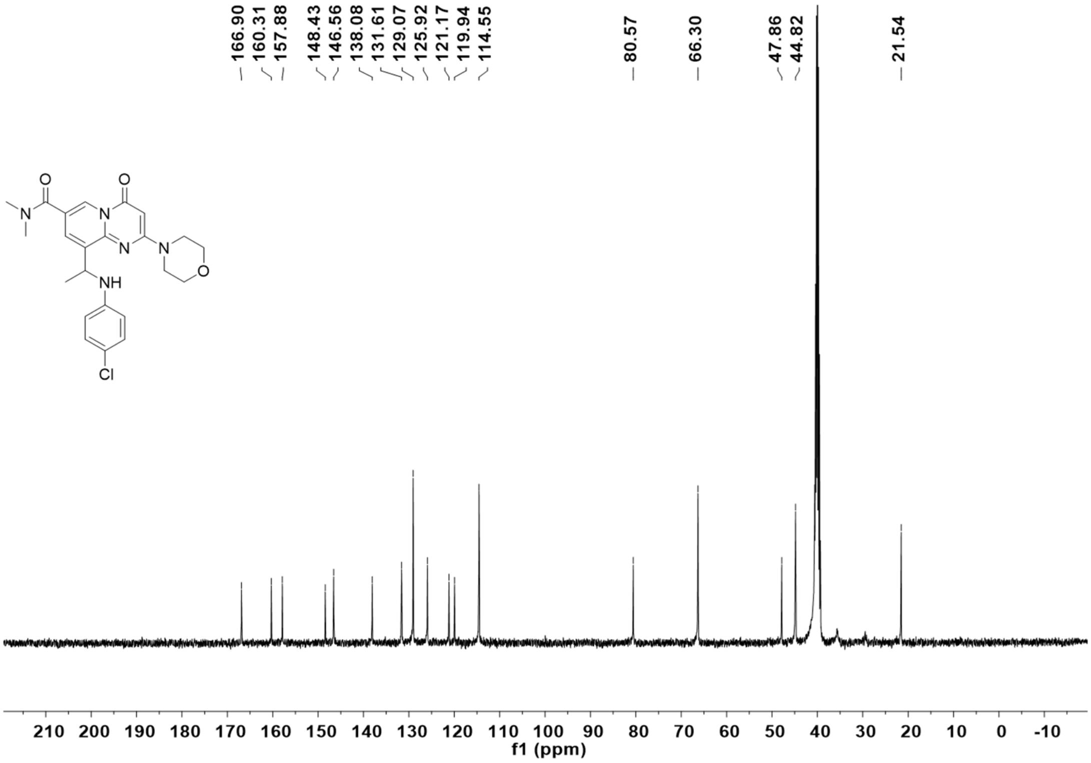

^{13}C NMR spectra of **MRS-4**

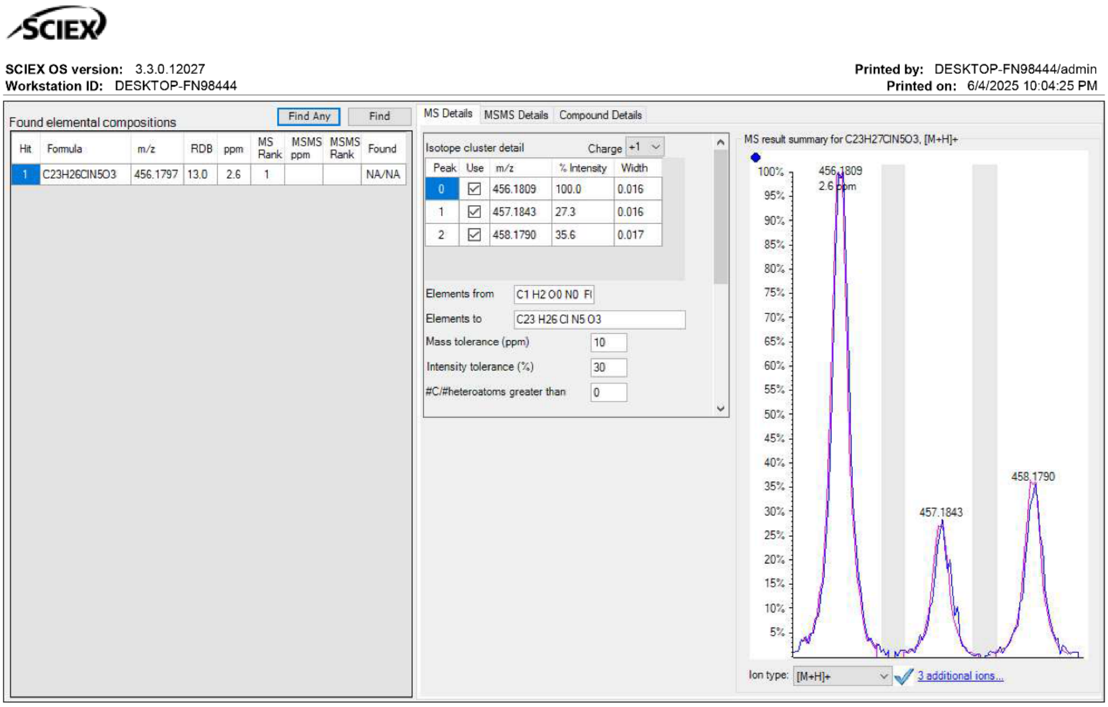

HRMS data of **MRS-4**

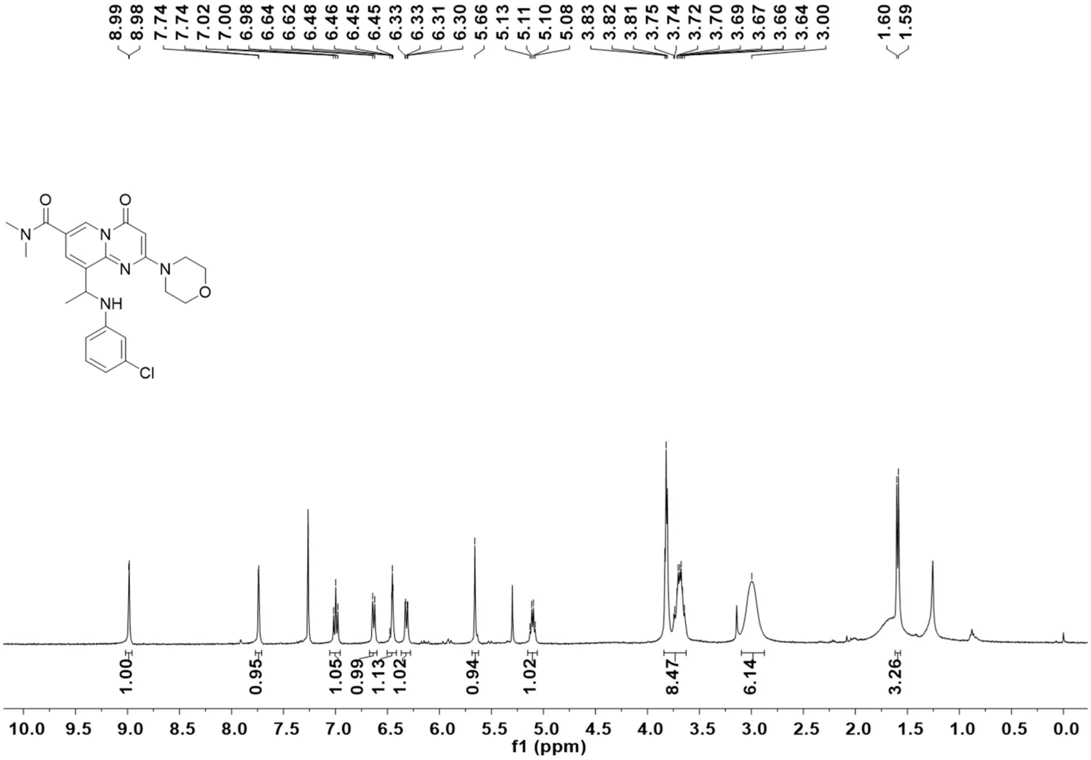

^{1}H NMR spectra of **MRS-5**

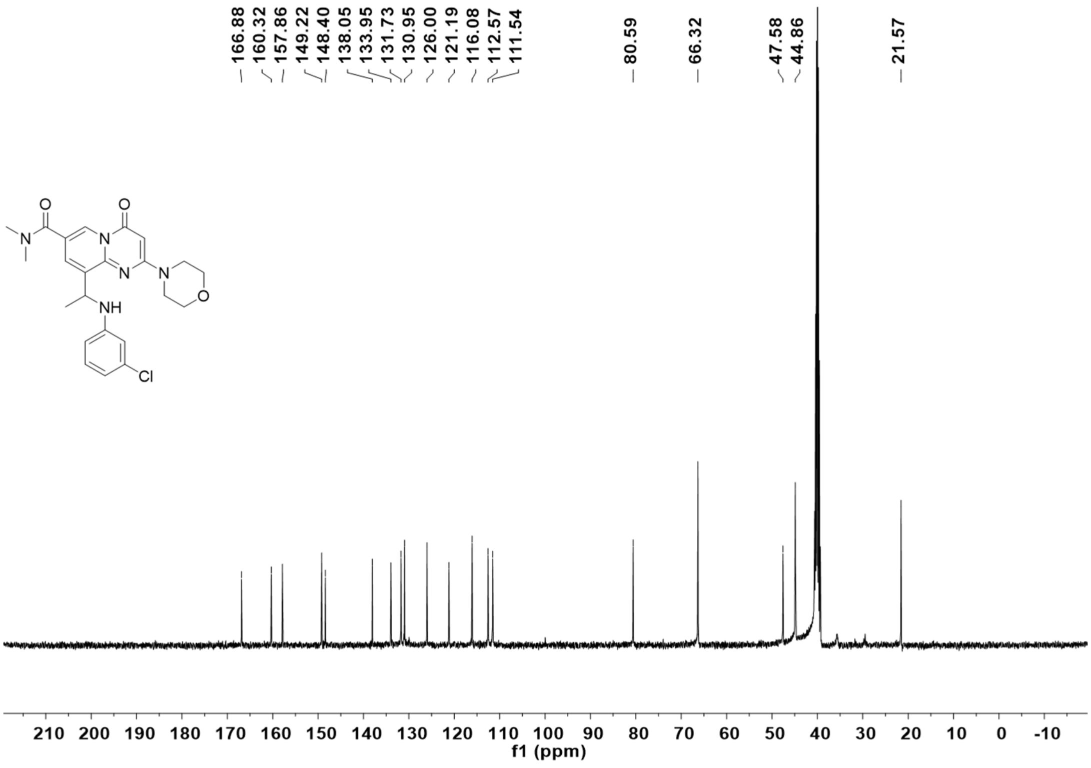

^{13}C NMR spectra of **MRS-5**

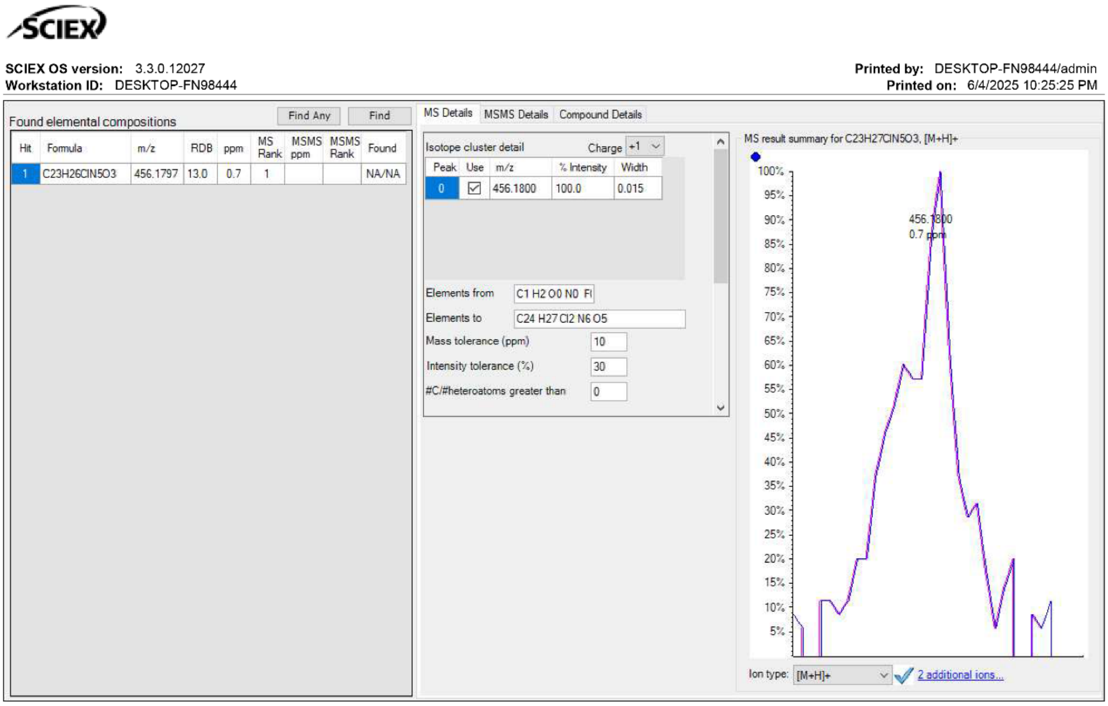

HRMS data of **MRS-5**

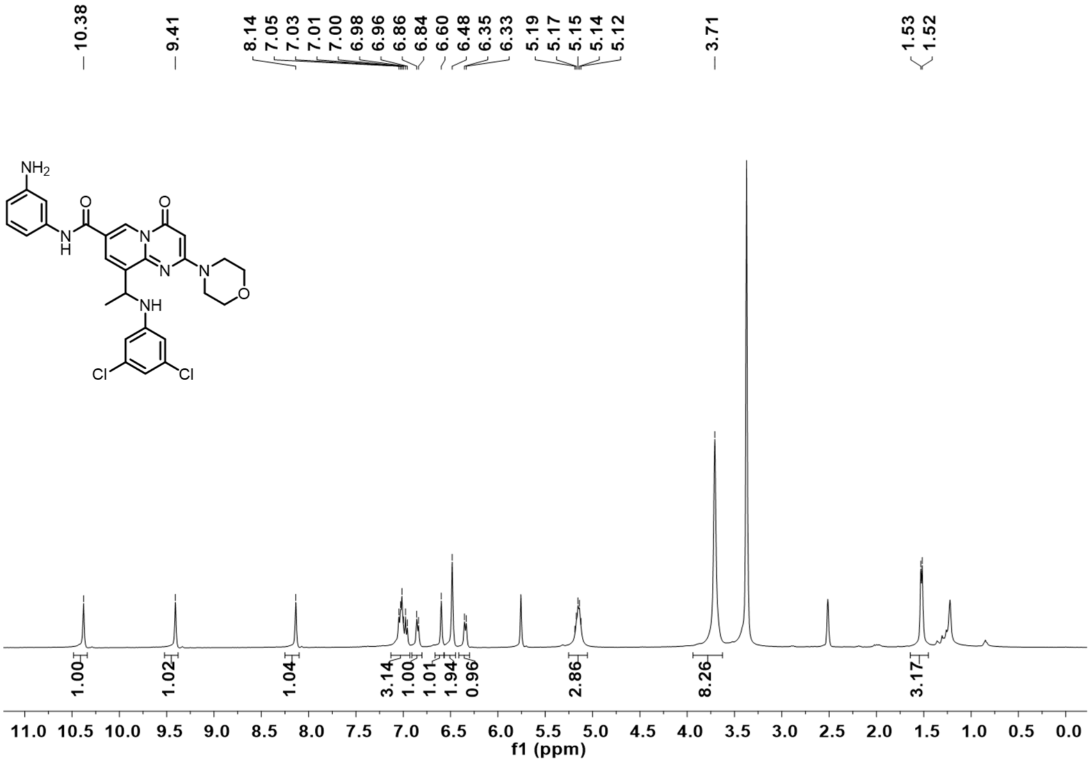

^{1}H NMR spectra of **MRS-6**

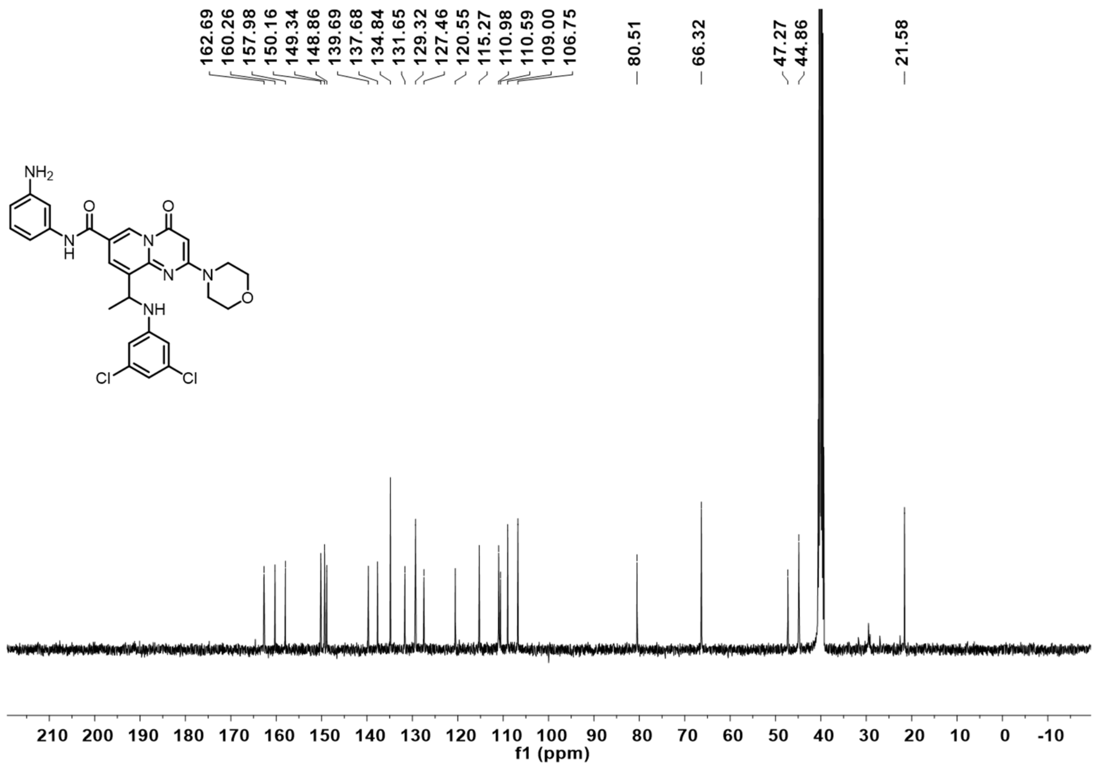

^{13}C NMR spectra of **MRS-6**

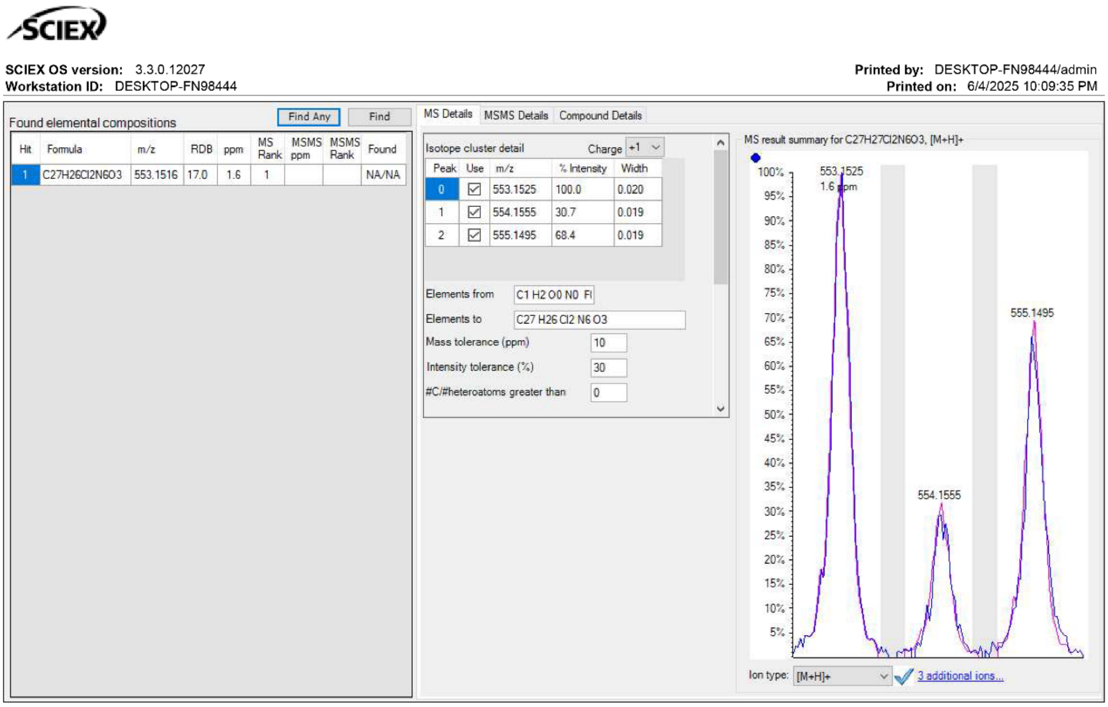

HRMS data of **MRS-6**

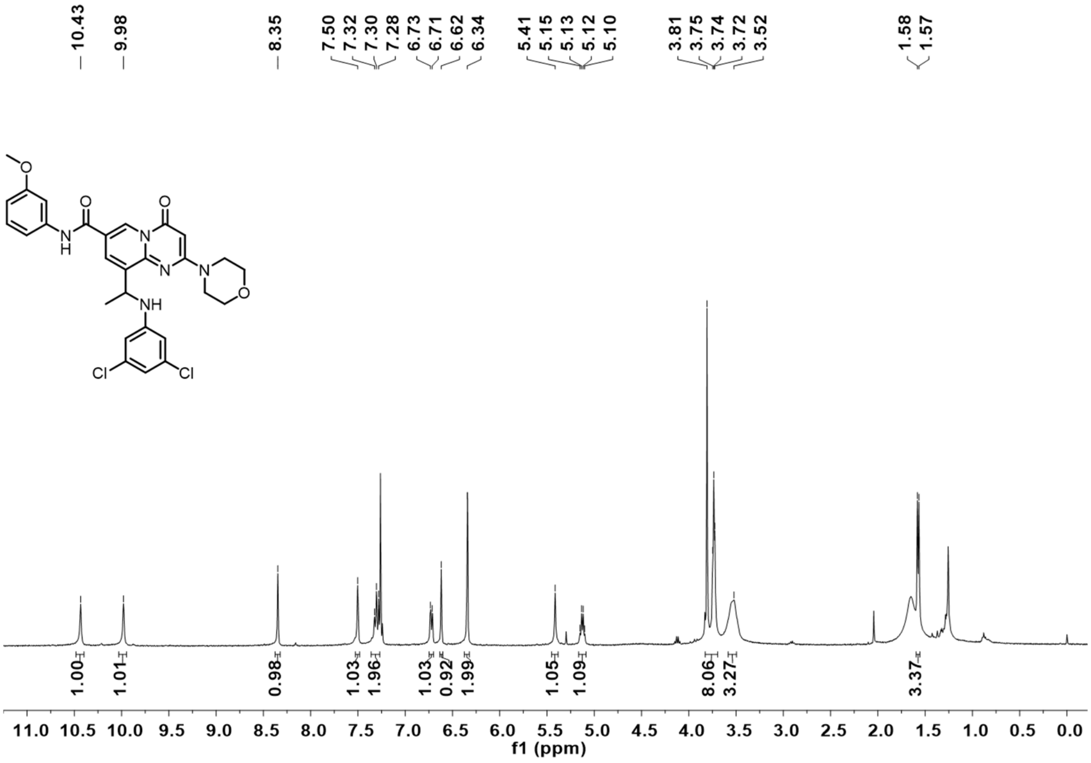

^{1}H NMR spectra of **MRS-7**

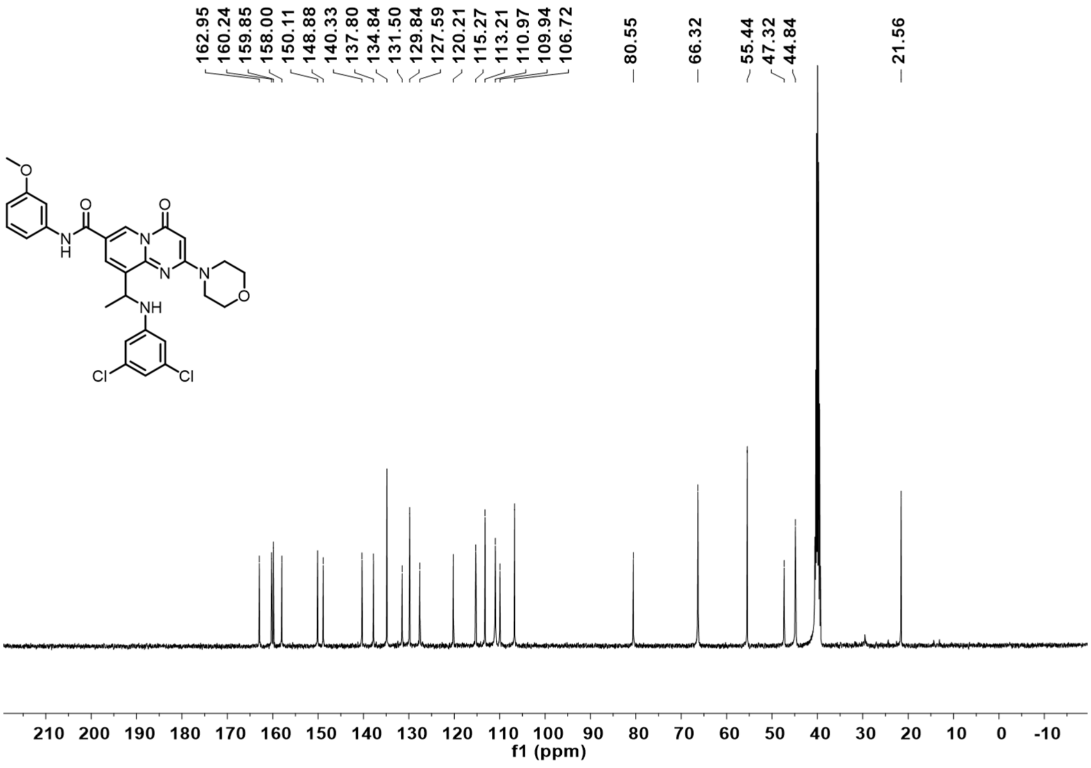

^{13}C NMR spectra of **MRS-7**

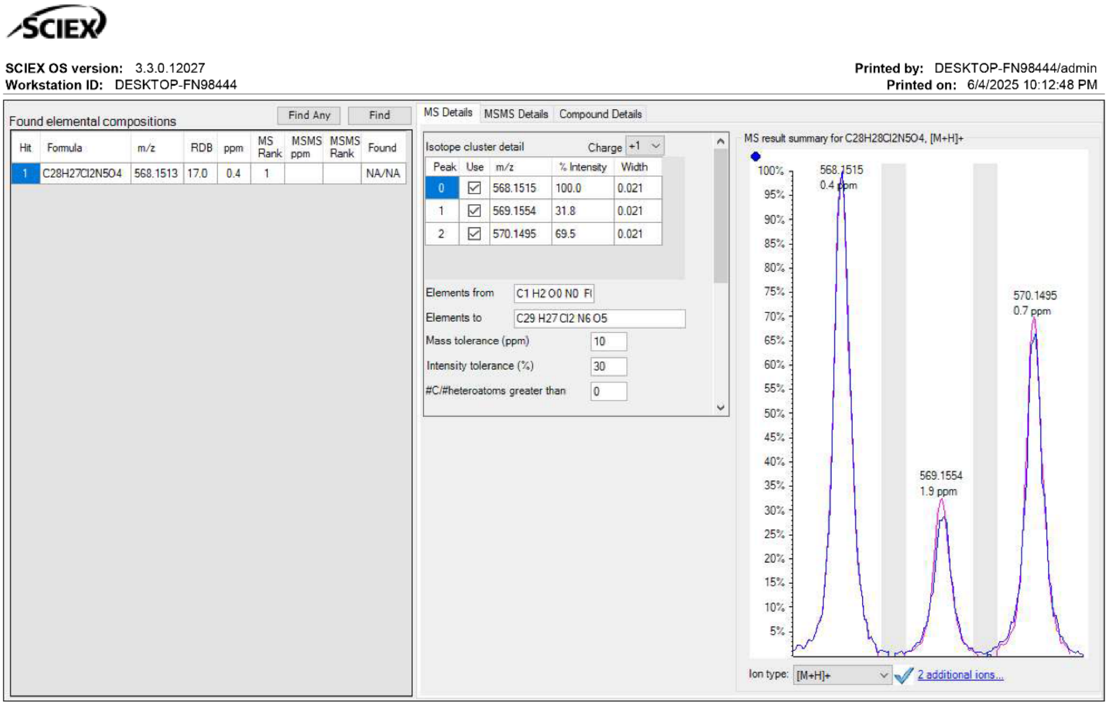

HRMS data of **MRS-7**

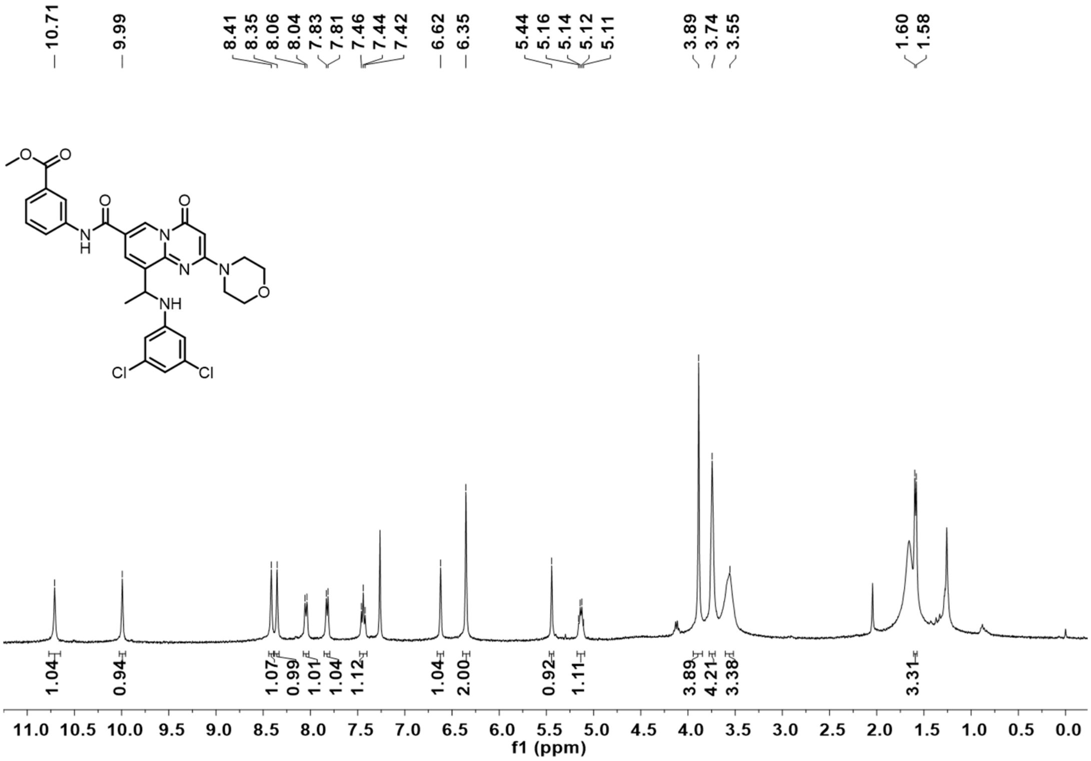

^{1}H NMR spectra of **MRS-8**

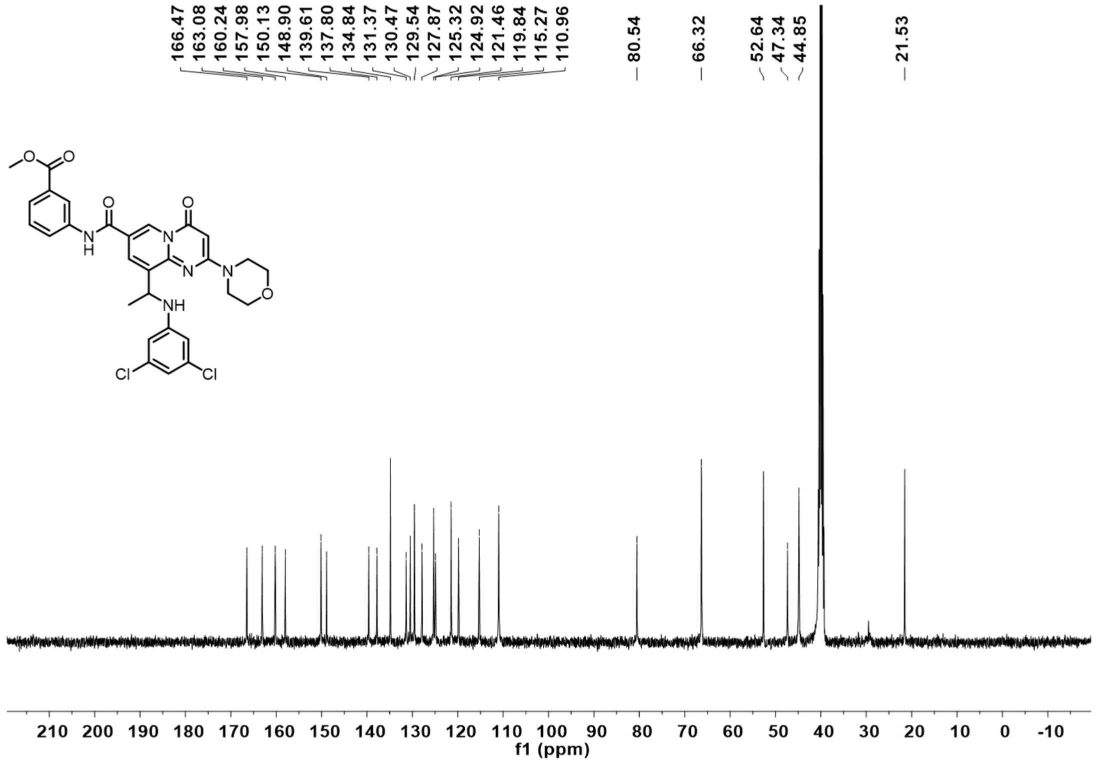

^{13}C NMR spectra of **MRS-8**

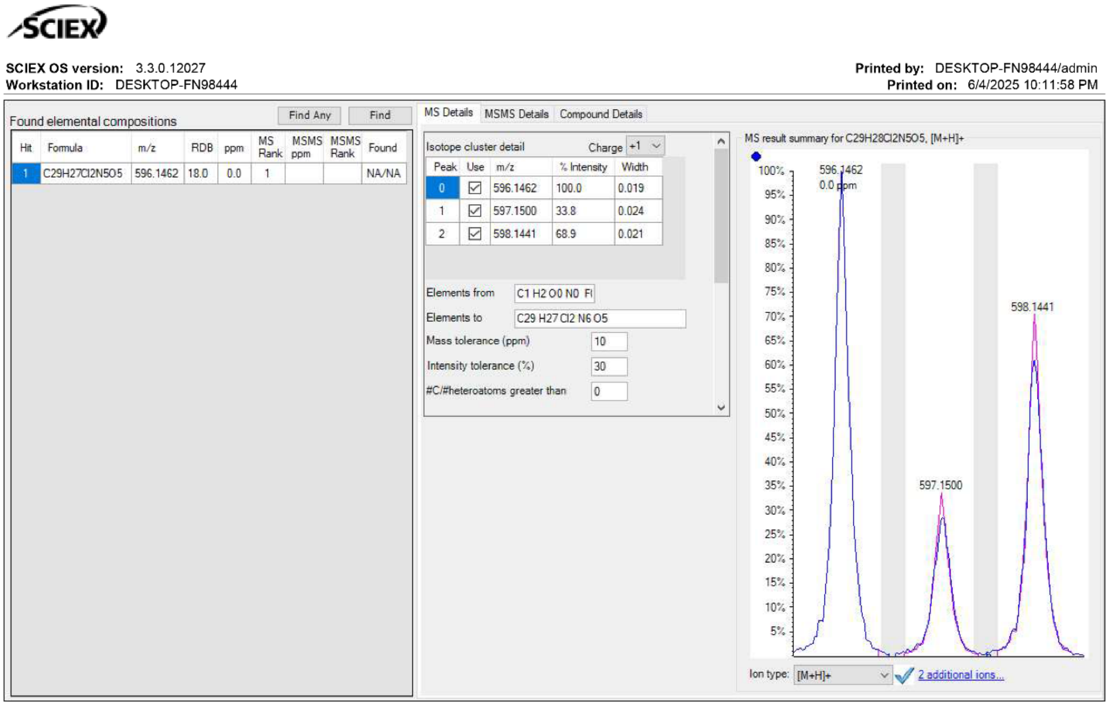

HRMS data of **MRS-8**

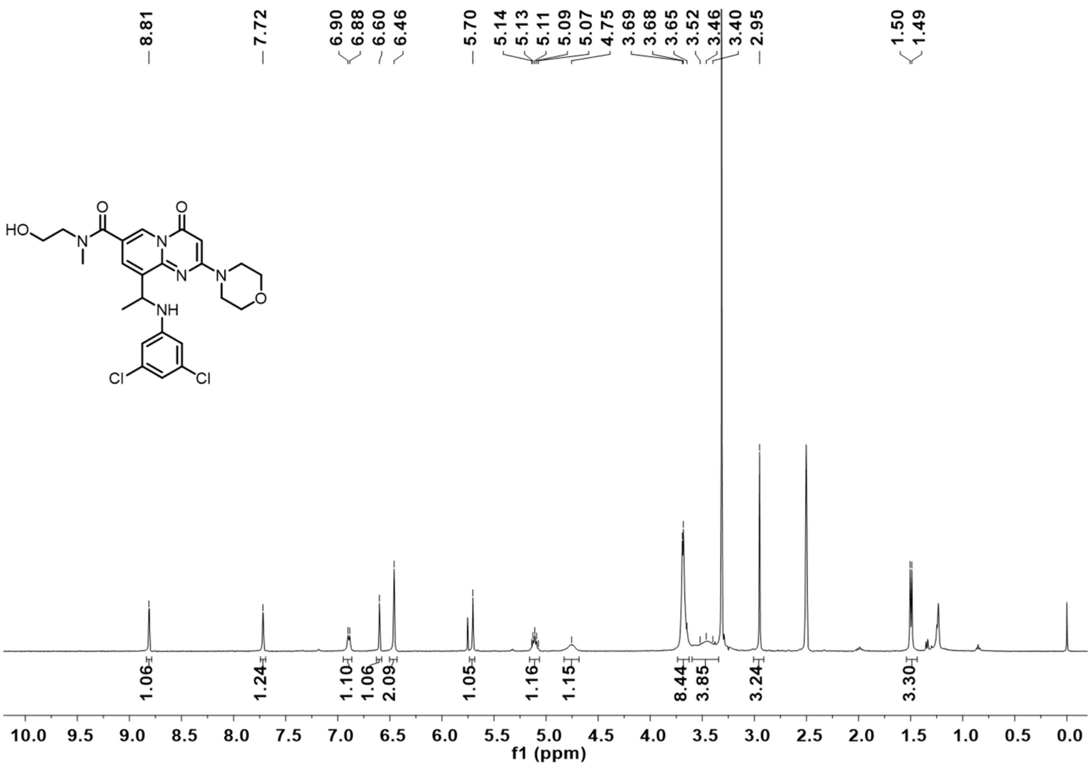

^{1}H NMR spectra of **MRS-9**

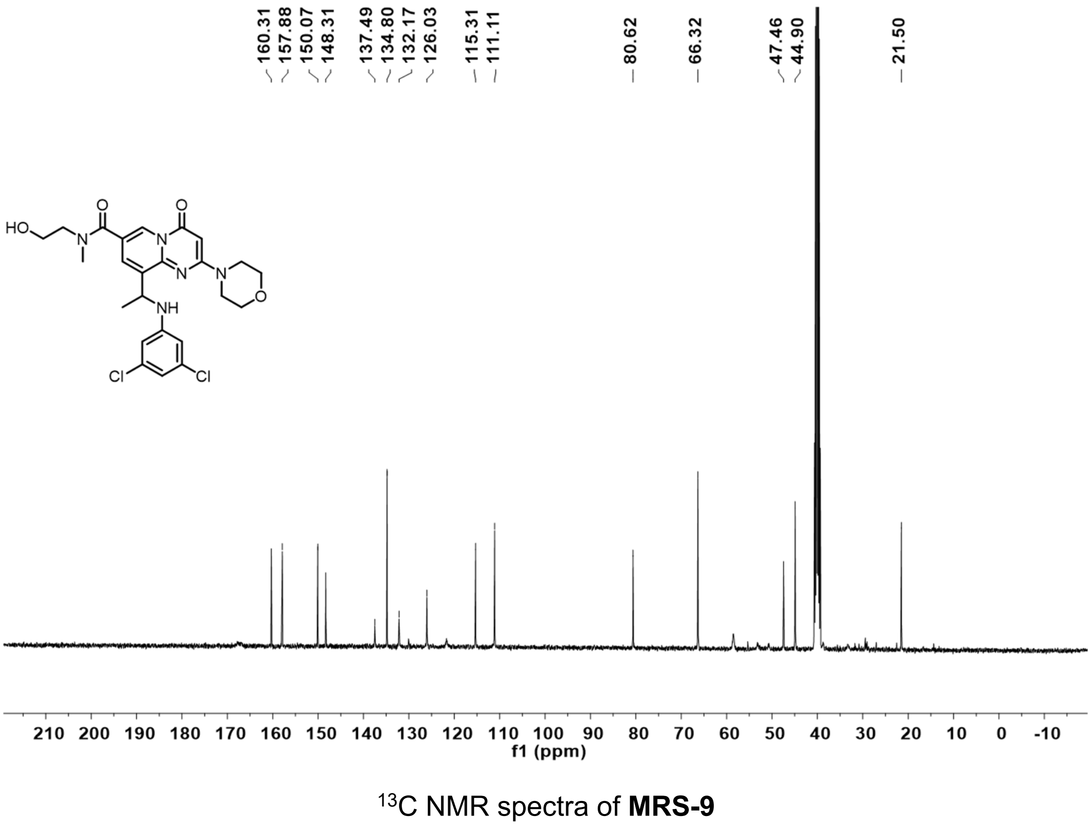

^{13}C NMR spectra of **MRS-9**

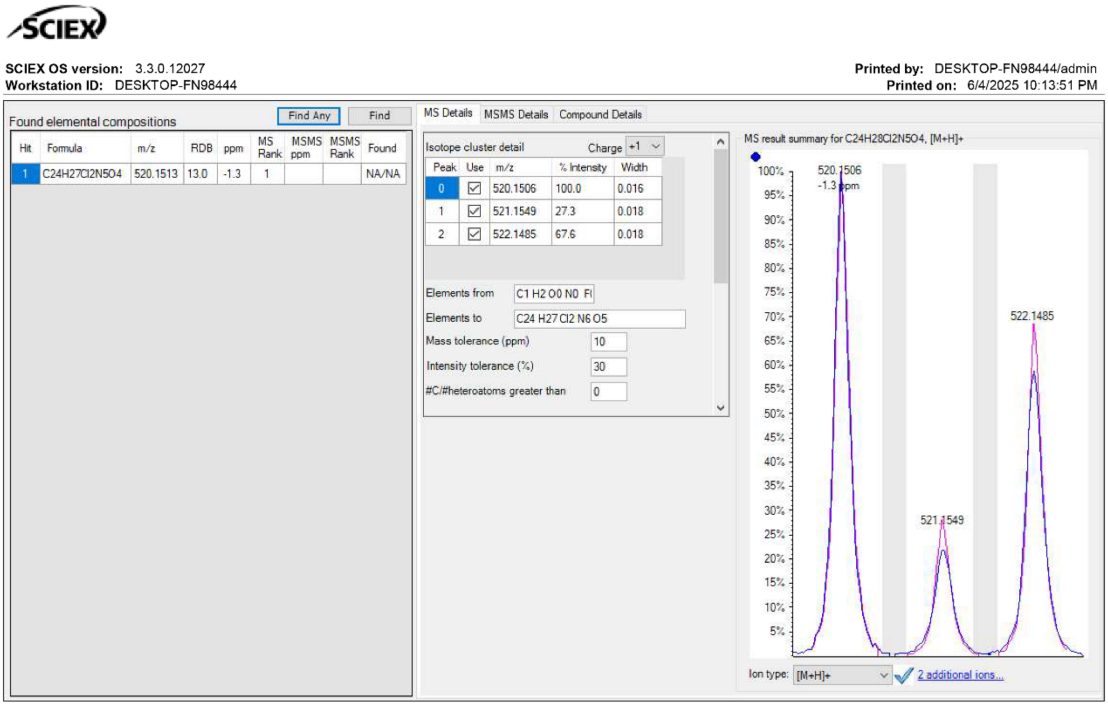

HRMS data of **MRS-9**

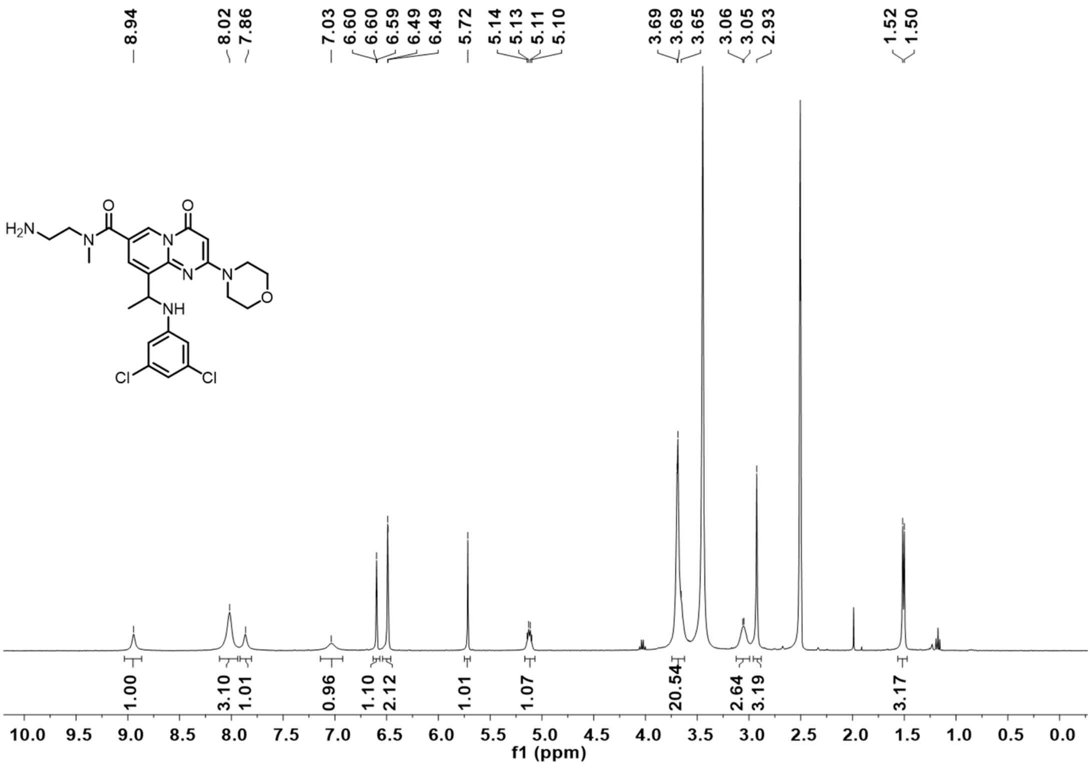

^{1}H NMR spectra of **MRS-11**

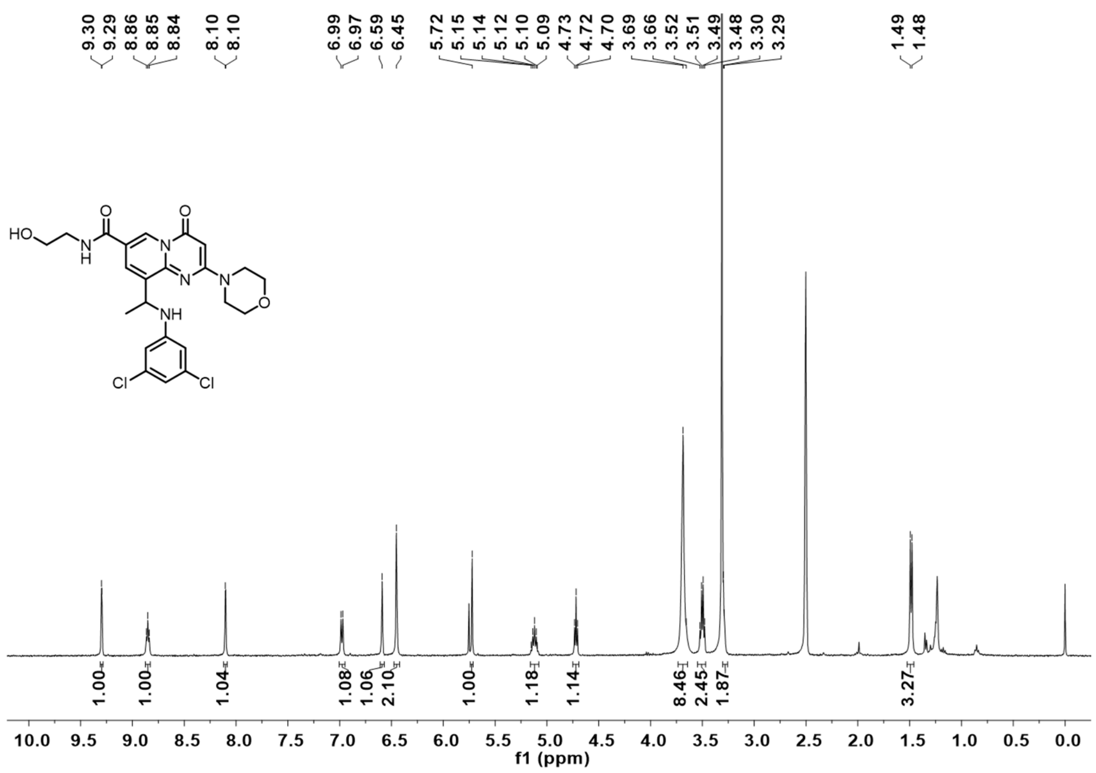

^{1}H NMR spectra of **MRS-12**

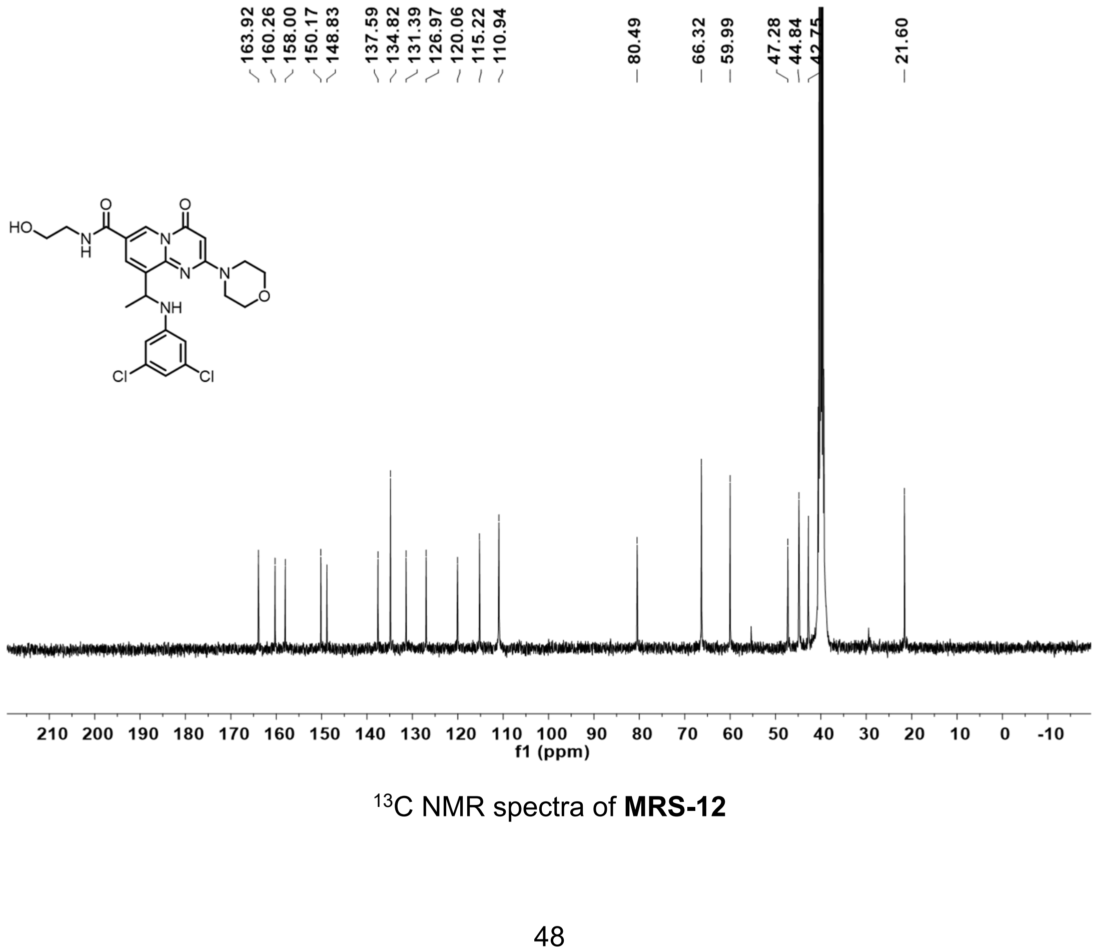

^{13}C NMR spectra of **MRS-12**

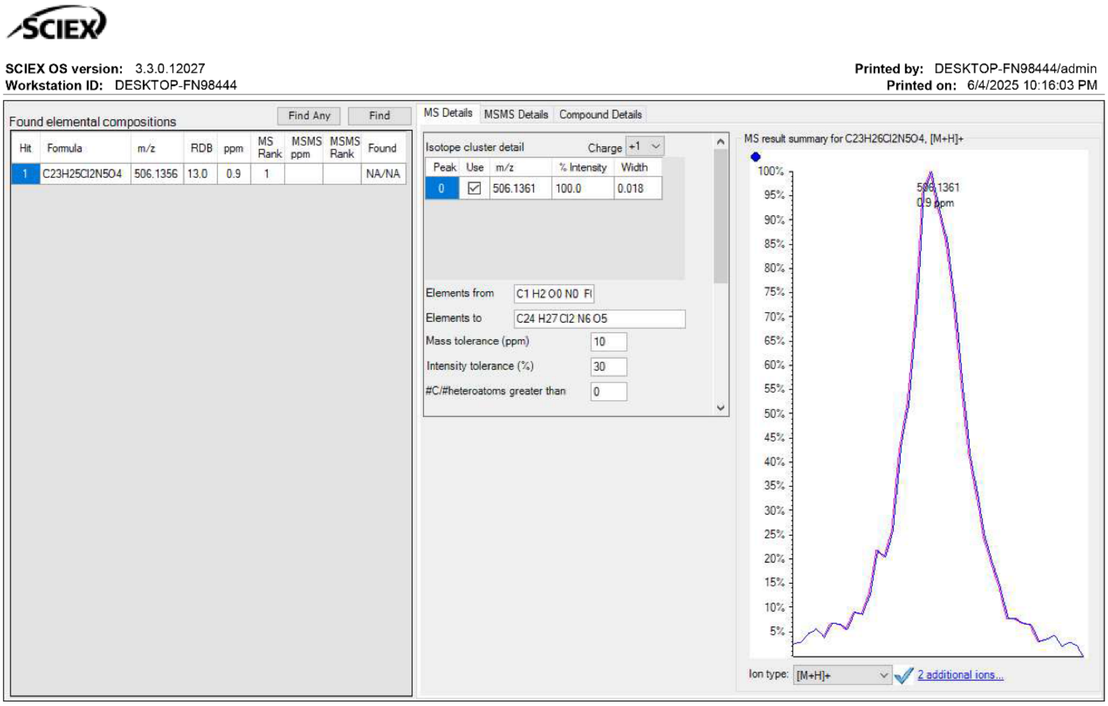

HRMS data of **MRS-12**

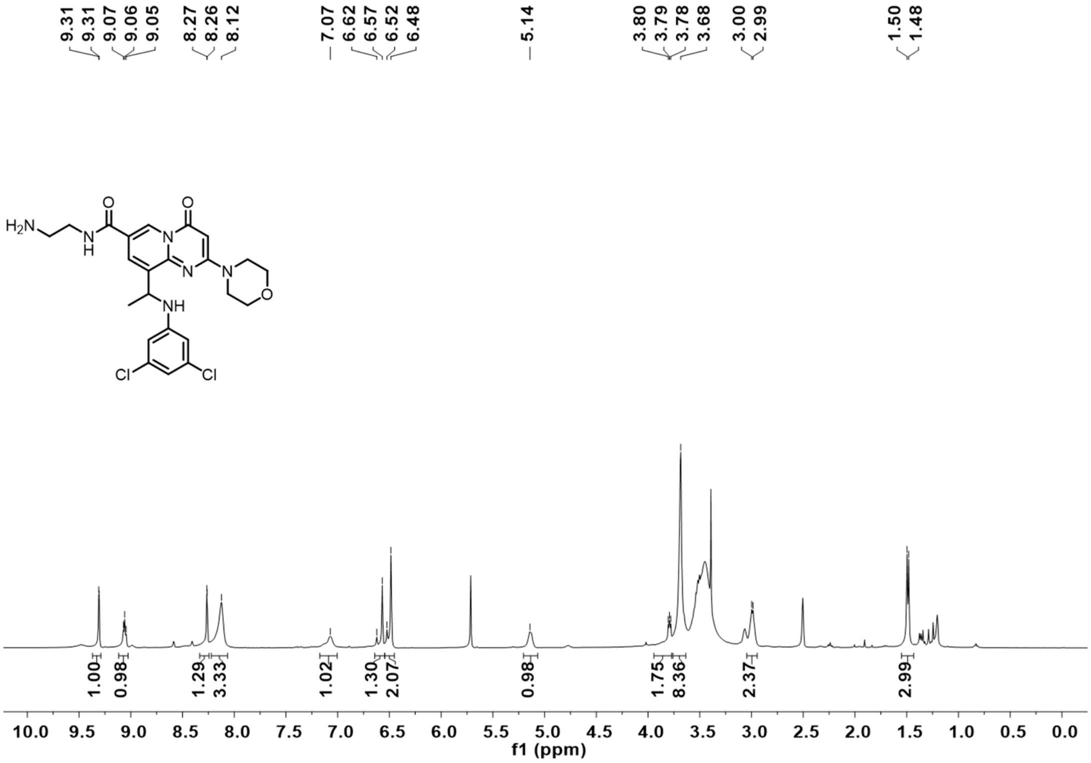

^{1}H NMR spectra of **MRS-13**

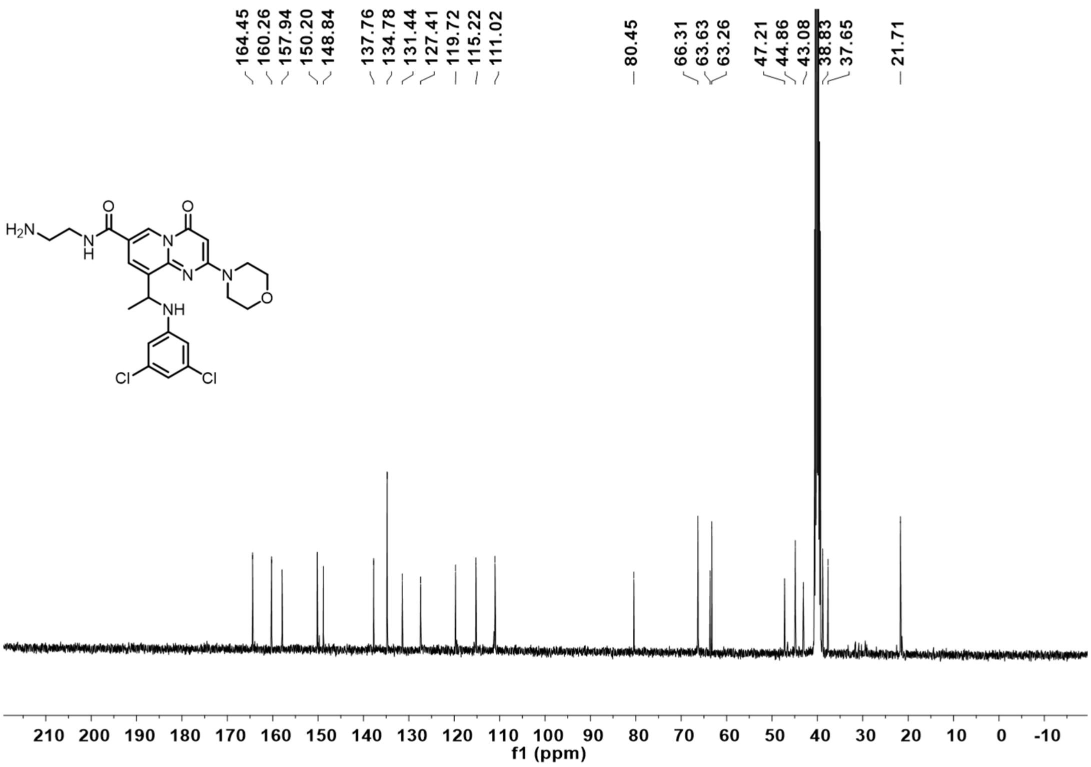

^{13}C NMR spectra of **MRS-13**

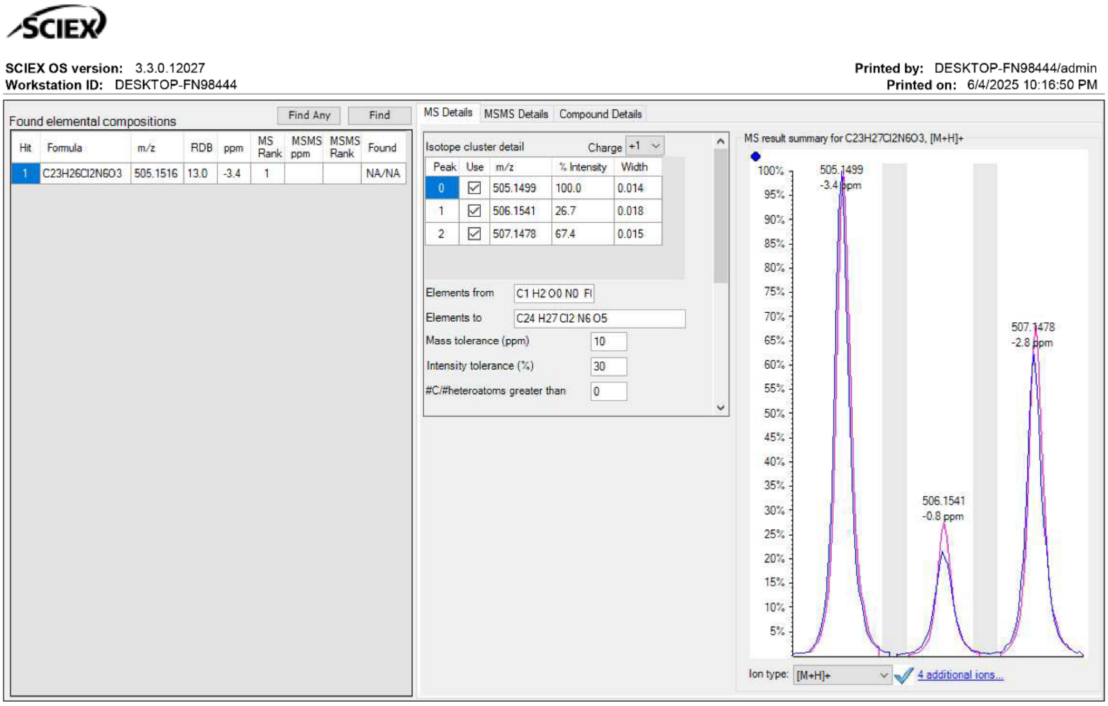

HRMS data of **MRS-13**

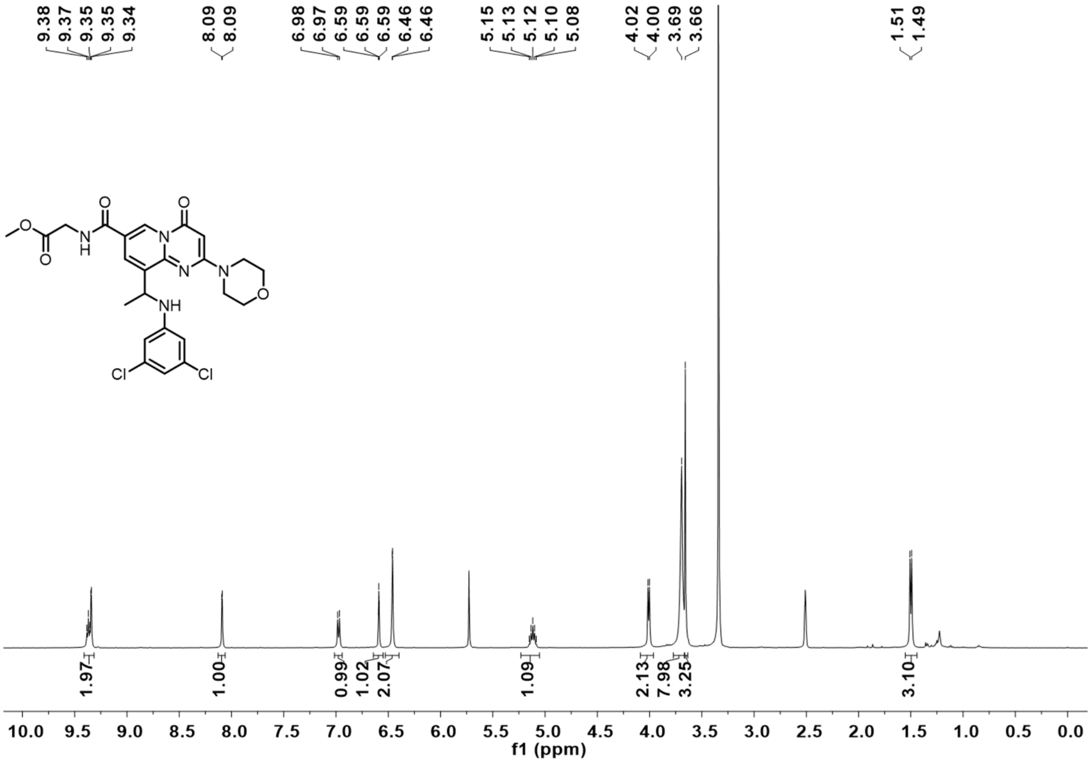

^{1}H NMR spectra of **MRS-14**

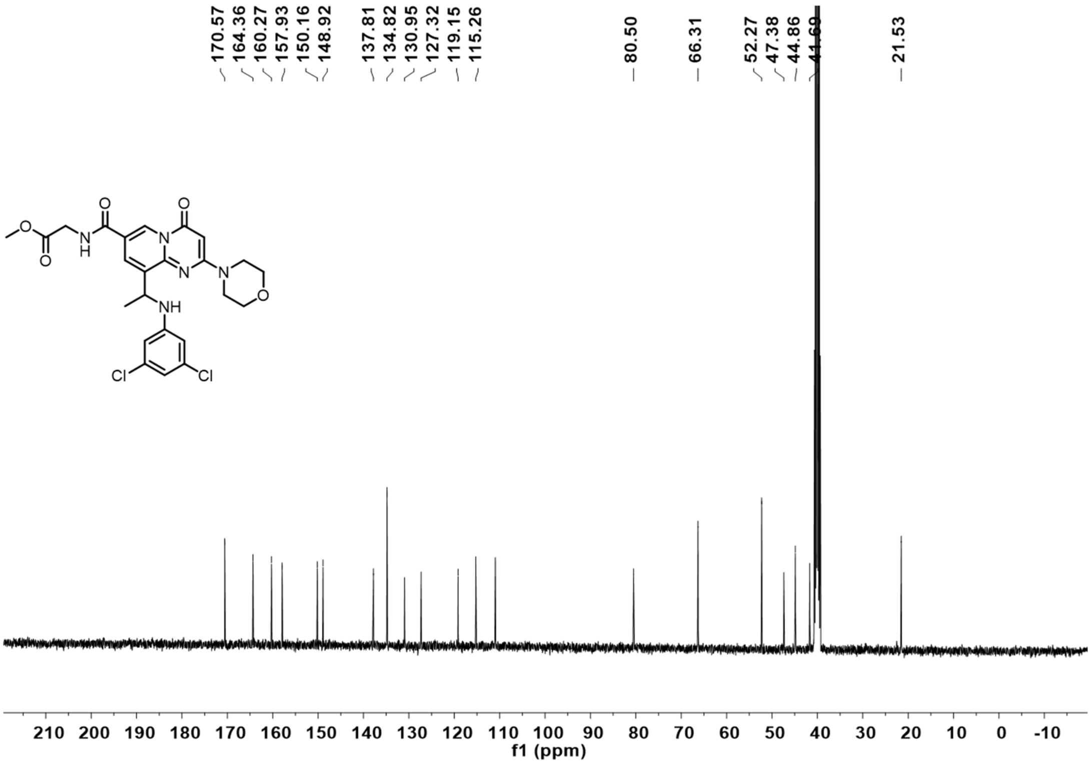

^{13}C NMR spectra of **MRS-14**

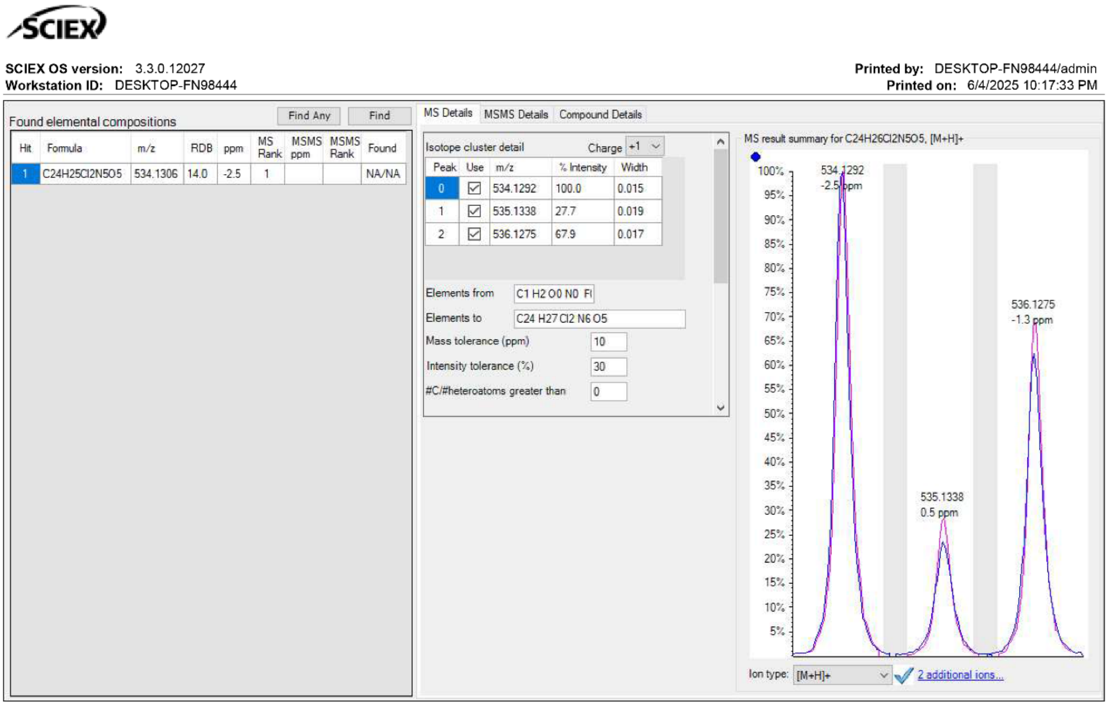

HRMS data of **MRS-14**

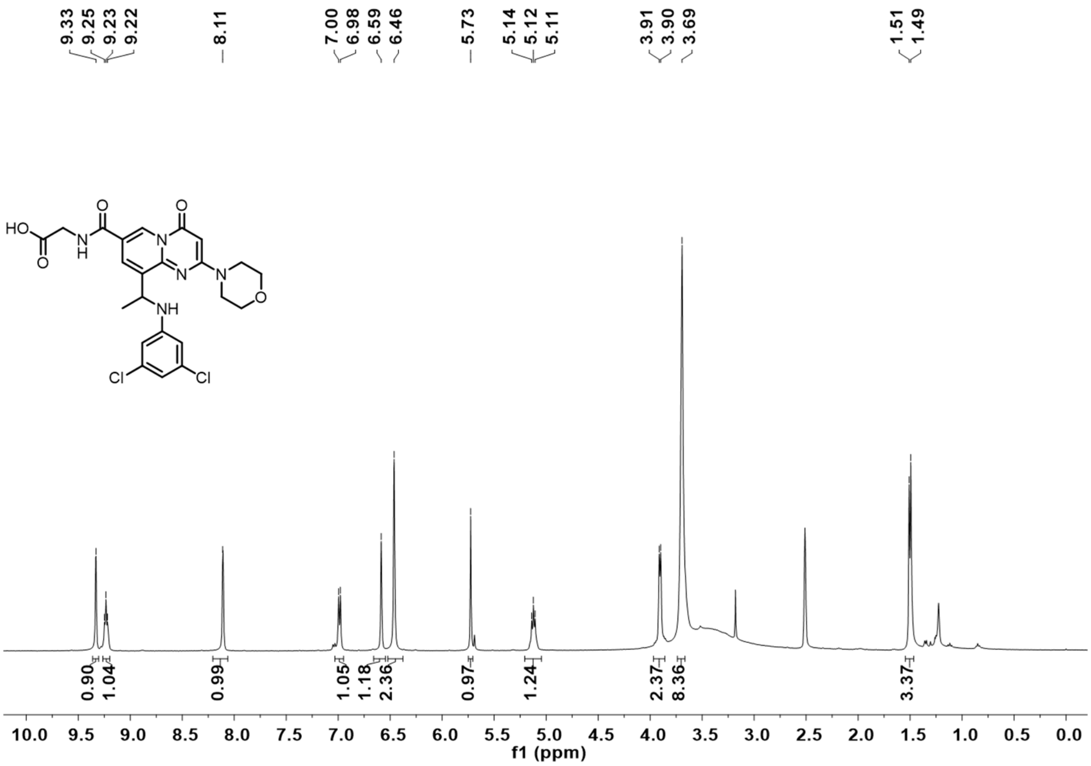

^{1}H NMR spectra of **MRS-15**

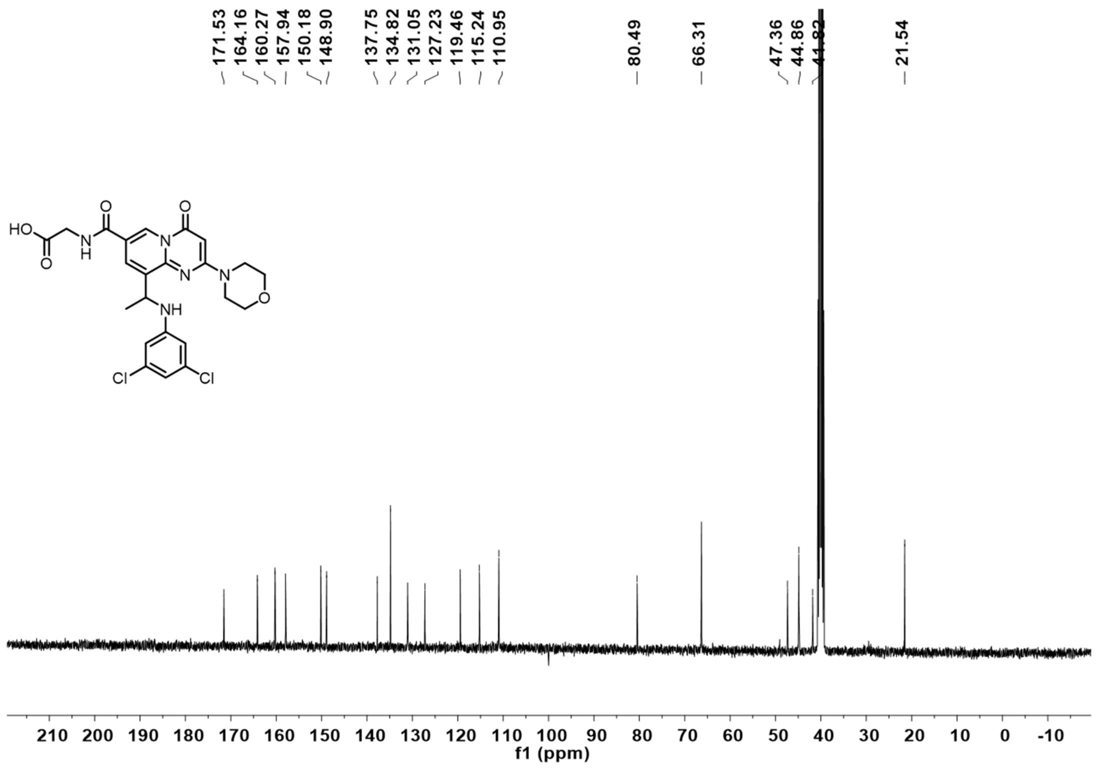

^{13}C NMR spectra of **MRS-15**

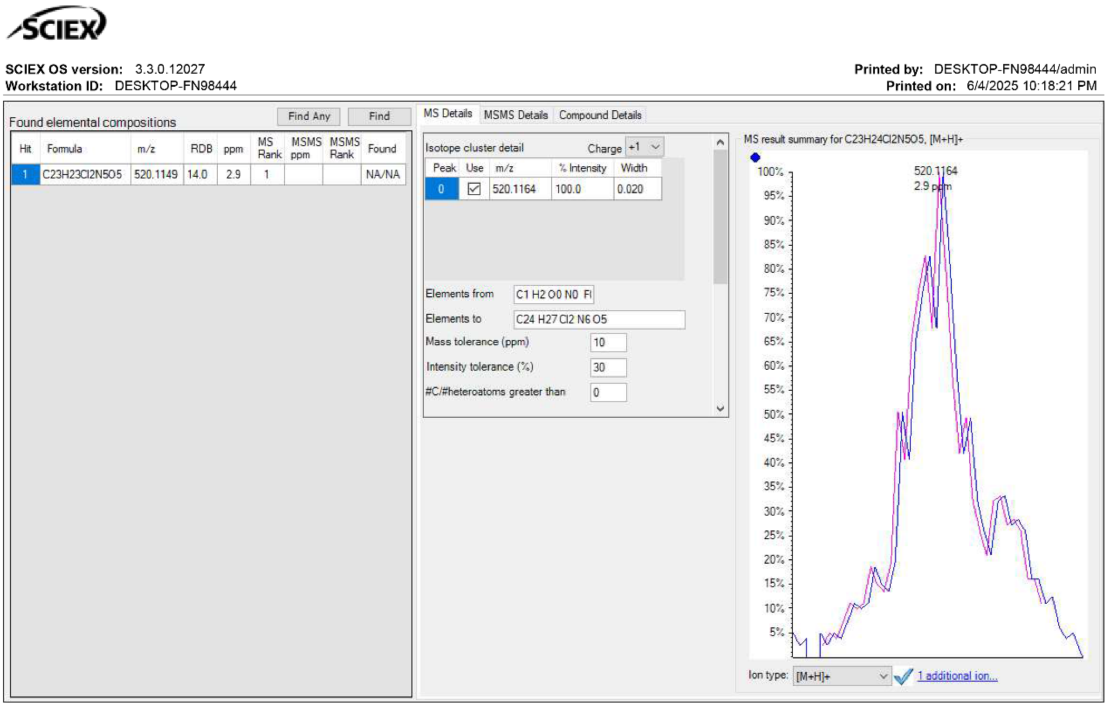

HRMS data of **MRS-15**

Supplementary References

1. Francoeur PG, Masuda T, Sunseri J, *et al.* Three-dimensional convolutional neural networks and a cross-docked data set for structure-based drug design. *J Chem Inf Model* 2020;**60**:4200–15.
2. Xie J, Chen S, Lei J, *et al.* DiffDec: structure-aware scaffold decoration with an end-to-end diffusion model. *J Chem Inf Model* 2024;**64**:2554–64.
3. Luo S, Guan J, Ma J, *et al.* A 3D generative model for structure-based drug design. *NeurIPS* 2021. https://openreview.net/forum?id=yDwfVD_odRo
4. Fialková V, Zhao J, Papadopoulos K, *et al.* LibINVENT: reaction-based generative scaffold decoration for in silico library design. *J Chem Inf Model* 2022;**62**:2046–63.
5. UniProt Consortium, Bateman A, Martin MJ, *et al.* UniProt: the universal protein knowledgebase in 2023. *Nucleic Acids Res* 2023;**51**:D523–D531.
6. Mirdita M, von den Driesch L, Galiez C, *et al.* Uniclust databases of clustered and deeply annotated protein sequences and alignments. *Nucleic Acids Res* 2017;**45**:D170–D176.
7. Remmert M, Biegert A, Hauser A, *et al.* HHblits: lightning-fast iterative protein sequence searching by HMM-HMM alignment. *Nat. Methods* 2012;**9**:173–5.
8. Huang Z, Yang L, Zhou X, *et al.* Protein–ligand interaction prior for binding-aware 3D molecule diffusion models. *ICLR* 2024. https://openreview.net/forum?id=qH9nrMNTIW
9. Schneuing A, Du Y, Harris C, *et al.* Structure-based drug design with equivariant diffusion models. *Nat Comput Sci* 2024;**4**:899–909.
10. Borgeaud S, Mensch A, Hoffmann J, *et al.* Improving language models by retrieving from trillions of tokens. *Proc 39th Int Conf Mach Learn* 2022;**162**:2206–40.
11. Sohl-Dickstein J, Weiss EA, Maheswaranathan N, *et al.* Deep unsupervised learning using nonequilibrium thermodynamics. *Proc 32nd Int Conf Mach Learn* 2015;**37**:2256–65.
12. Ho J, Jain A, Abbeel P. Denoising diffusion probabilistic models. *Adv Neural Inf Process Syst* 2020;**33**:6840–51.
13. Kingma DP, Salimans T, Poole B, *et al.* Variational diffusion models. *arXiv* 1 July 2021. https://arxiv.org/abs/2107.00630 (2021).
14. Waterhouse A, Bertoni M, Bienert S, *et al.* SWISS-MODEL: homology modelling of protein structures and complexes. *Nucleic Acids Res* 2018;**46**:W296–W303.
